# The Occurrence of 275 Rare Diseases and 47 Rare Disease Groups in Italy. Results from the National Registry of Rare Diseases

**DOI:** 10.3390/ijerph15071470

**Published:** 2018-07-12

**Authors:** Domenica Taruscio, Luciano Vittozzi, Adele Rocchetti, Paola Torreri, Luca Ferrari

**Affiliations:** Centro Nazionale Malattie Rare, Istituto Superiore di Sanità, 00161 Rome, Italy; cnmr.eu@iss.it (L.V.); adele.rocchetti@iss.it (A.R.); paola.torreri@iss.it (P.T.); webcnmr@iss.it (L.F.)

**Keywords:** rare diseases, national registry, data quality, epidemiology, Italy

## Abstract

Knowledge of rare diseases (RD) is often scattered among many data collections and registries of patient cohorts. Therefore, assessing the burden of RD in the general population, developing appropriate policies and planning services for the care of RD patients is difficult. This study aimed at providing a systematic picture of RD occurrence in a population as big as 60 million. Data of diagnoses were certified and collected by a network of 247 specialized centres covering the whole Italian territory. Data received (about 200,000 records) were validated according to formal criteria and, where necessary, corrected by the data sources. Data of age at onset and sex distribution are given for about 400 diseases. Incidence and/or birth prevalence are given for 275 diseases and 47 disease groups, which, altogether, comprise a substantial part of the known rare diseases. Data quality, internal consistency, and external validity of the database have also been assessed and ways to limit the impact of some discrepancies were devised. The information provided by RNMR, cutting across such a wide range of RD, represents a unique coherent basis allowing the prioritization of relevant public health measures and research activities.

## 1. Introduction

Rare diseases (RD) are complex diseases with a very low prevalence (in the EU, a prevalence of less than 5/10,000 in the general population is considered). These conditions are currently estimated to be as many as 6000–8000 and the International Classification of Diseases codes a very limited number of them. Consequently, hospital health records are often not the appropriate sources to get information on RD. Therefore, knowledge on RD is often based on data collections and registries resulting from academic and commercial interests. ORPHANET censused a total of more than 700 registries and databases on RD [1] involving European scientists. They address a variety of aims and differ in their organization, quality and database structure, usually monitoring one disease or a group of related diseases [2]. Moreover, many studies are based on hospital data in regions with a higher prevalence [3]. This situation results in the difficulty of assessing the burden of RD in the general population and developing appropriate priority policies for the care of RD patients and the planning of health and social care services. To overcome these difficulties and support the development of knowledge on RD, the USA NIH-NCATS launched the Global RD Patient Registry and Data Repository—GRDR [4], and the EU Council issued a Recommendation urging Member States to support specific disease information networks, registries, and databases [5]. The EU also supported several initiatives addressing the better exploitation of RD patient data [6,7,8,9,10,11]. All these efforts envisage a system addressed to the registration of many, not to say all, RD and to the improvement of data interoperability. The need for efficient and extensive registries for RDs is also made urgent by international cooperations, such as IRDiRC [12] and the European Reference Networks [13], as well as by the establishment of National Registries [14].

The specificities of RD and the special needs of RD patients were recognized in Italy in 2001 when the National Network of Accredited Centres for the assistance and care of RD patients was established by the Ministerial Decree (MD) 279/2001 [15]. This Decree mandated the notification of new cases to the National Registry of Rare Diseases (RNMR), through Regional Registries (RR). The RNMR central database was placed in the National Institute of Health and has been managed by the National Centre for Rare Diseases since its establishment. This registry can be of interest to the current efforts of promoting data comparability and data retrieval across registries for many reasons. RNMR is organized as a devolved system of 17 regional registries, two registries of Autonomous Provinces, and one interregional registry (Piemonte and Valle d’Aosta), which cover populations spanning from about 10.0 to 0.3 million residents. They are controlled by regional or provincial authorities, which make independent decisions and have their own peculiar needs and resources regarding the delivery of care. Therefore, RNMR may represent a pilot on the feasibility and use of the cooperation among national registries, which will seemingly be developed by mainly following the needs of different countries. Moreover, the set of data collected in the central repository very closely resembles the mandatory Common Data Set proposed by EPIRARE [16], which aimed at building synergies among public health authorities, researchers, patients, and the industry for fulfilling their information needs. To this aim, the EPIRARE Common Data Set was devised to support the production of indicators useful for decision-making in public health and research on RD [6,16,17], as well as to fulfill some patients’ information needs. A recent publication by a regional registry which contributes to RNMR, provided some indicator data relevant to RD and their care [18].

After achieving the full coverage of the national territory in 2011 monitoring, individually, 284 RD and, collectively, many more RD distinguished in 47 groups, it is now possible to use the data contained in RNMR. In this paper, we aim to provide a detailed and systematic picture of the epidemiology of RD in Italy, with particular reference to patients requiring RD dedicated assistance and care. The results have been obtained from 247 accredited hospitals, which cover the whole national territory and serve a population of 60.4 million residents. This population is characterized by a sex ratio of 0.94 (males:females), while the youth (0–14 year) and 65+ people make up 13.9% and 21.4%, respectively. In 2017, non-European foreigners were mostly from Western and Northern Africa (1.70% residents), Eastern and Central-Southern Asia (1.66%), and Latin America (0.58), with all other origins accounting for 0.22%. Since this is the first time that an analysis of RNMR data has been published, special attention has been devoted to assessing and describing the validity of the data collected.

## 2. Materials and Methods

### 2.1. Set of Data Communicated to RNMR and Collection Procedures

The data communicated to RNMR by the RR and used for further elaborations were:

- Personal identifiers of the patient: name, surname, place and date of birth, sex, and national ID code (represented by the Fiscal Code and by an Encrypted Univocal Patient Code derived from it)
- Patient place of residence
- Disease diagnosed (defined according to the exemption codes listed in MD 279/2001 [15])
- Center making the diagnosis, with geographical location (coded according to the National Health Service Information System [19])
- Date of disease onset
- Date of diagnosis

Data, which referred to the time point when the RD diagnosis is certified by an Accredited Centre, was communicated immediately to its RR by the Accredited Centre itself. This procedure was formally agreed with the regional authorities in 2007 [20] and ensures that diagnoses are notified based on the most reliable diagnostic protocols and best expert advice. Following this agreement, two slightly different record structures were developed by the centers to better serve their regional health service organization. In some regions, the record reported the centre which ascertained the RD diagnosis for the first time and the Accredited Centre notifying the case, while, in other regions, the record indicated the Accredited Centre entitled to formally certify the diagnosis and notify the case. Correspondingly, the date of diagnosis was reported as the date of the first ascertainment of the diagnosis or as the date on which the diagnosis was formally certified and notified. Piemonte, Valle d’Aosta, and Lombardia communicated both the dates of diagnosis ascertainment and certification, while Sardinia switched from the former to the latter record structure in 2012–2013. The date of diagnosis is a main reference for data selection and elaboration. Therefore, data were kept distinct: records characterized by the diagnosis ascertainment date are indicated as “AD notifications” and the regions using this record structure are referred to as “AD regions”. The records characterized by the diagnosis certification date are indicated as “CD notifications” and the related regions as “CD regions”. The coexistence of two notification practices had an important bearing on the epidemiological data. Indeed, as it will be shown in more detail in the results section, frequency data of a disease in a regional population were usually a mix of data notified by AD and CD regions, due to the migration of patients from the residence region to another region, which could confirm the RD diagnosis and notified the case. Finally, Friuli-Venezia Giulia (FVG) communicated no personal identifiers in compliance with local personal data protection regulations. Procedures of data collection and communication have been described elsewhere [21].

The collected data set and the data flow were established and made mandatory by the supporting MD 279/2001 [15]. Therefore, no patient consent nor ethical approval was required. Data were processed according to the EU and Italian data protection regulations in all participating structures. Data processing procedures were communicated to the National Authority for Personal Data Protection and their approval was obtained.

### 2.2. Aims, Procedures and Limitations of Data Curation

Data curation aimed at spotting erratic or inconsistent data, at discarding records that referred to cases diagnosed after the end of the reference period (31 December 2014) and at detecting and managing duplicate records. Data communicated by RR were checked upon their reception by the National Centre of Rare Diseases. Regional duplicates (i.e., records sent by the same region, referred to the same national ID code) and records showing data not passing the validation criteria (detailed in Supplementary File 1), were fed back to the experts responsible for pertaining the RR, for them to perform the necessary checks, even with the primary data sources, and to confirm or correct the data. After receiving the data sets resulting from these controls, the residual mistakes and discrepancies were recorded; then, the name, surname, place of birth, and Fiscal Code of the patient were removed by all records and the place of residence of the patient was substituted by her/his region of residence; finally, the regional data sets were merged into a single file. In this file, national duplicates (i.e., records with the same Encrypted Univocal Patient Code) were detected, classified, and managed to obtain the subsets to be used according to the scope of the analyses, as reported in Supplementary File 1 (Table S1. Control of duplicate records and curation of demographic data from Friuli-Venezia Giulia (FVG) could not be carried out. Data curation was accomplished by means of an Excel model.

### 2.3. Symptoms Onset Age and Distribution between Sexes

These features have been calculated from the data of Subset 1 for diseases with at least four records with valid data. For these analyses, data of individual diseases identified with their specific denomination were used, including those coded with group codes.

### 2.4. Birth Prevalence and Incidence of Rare Diseases

The data used for the calculation of the incidence rates refer to cases with date of diagnosis ascertainment or certification included in the period from 1 January 2012 to 31 December 2014. The data selected for the assessment of birth prevalence (BP) were limited to cases born between 1 January 2012 and 31 December 2013 and with diagnoses ascertained or certified during the first year of life of the case. In these calculations, the dates of diagnosis ascertainment were only considered in the records from Piemonte, Val d’Aosta, and Lombardia. Records were analysed by codes, so that diseases coded with group codes were not analysed individually.

The calculations of regional incidence and BP rates were carried out as follows. It was assumed that differences in the frequency of each pathology, as resulting from either AD or CD notifications, were mainly due to the different local sensitivities of two notification practices rather than to changes of RD frequencies over time. Therefore, for each pathology and each region, the fraction of AD or CD notifications in their respective reference periods were expressed in percent with respect to the total AD plus CD notifications. The notification practice, which represented at least 90% of total notifications of a region over its reference period, was used to calculate the disease incidence and BP in that region. For those diseases and regions where neither notification system reached 90% total regional notifications, the calculation was not carried out. The assumption was applied considering that the datasets from Piemonte, Valle d’Aosta, and Lombardia, which recorded both dates, showed differences between dates of ascertainment and dates of certification of 4.9 years (95% Confidence Interval: 4.4–5.4 years), 8.5 years (95% CI: 6.1–10.9), and 5.3 years (95% CI: 4.9–5.7), respectively (data not shown).

Mean national incidence and BP rates for the three-year period of 2012–2014 were estimated with the same assumption as above, that the frequency of new cases in the period defined by the diagnosis certification date did not differ significantly from the frequency of new cases in the period defined by the diagnosis ascertainment date. Therefore, AD and CD notifications were merged and their sum was used for national frequency estimates. Regional populations on 31 December 2012, 2013, and 2014 [22] and regional live births in the two-year period of 2012–2013 [23] were used to calculate the denominators for regional and national data.

### 2.5. Internal Consistency of the Incidence and Birth Prevalence Data and Comparison with Literature Data

The internal consistency of the RNMR database has been checked by comparing the medians of the regional incidence and BP rates obtained from AD and CD notifications. The comparison with literature data was limited to the diseases which were monitored by RNMR and could be traced with certainty to an ORPHA code. In addition, comparisons of BP data were limited to diseases which showed a median onset age lower than six months. Results from the RNMR database were firstly compared with literature data reported by ORPHANET [3,24]. In cases where differences of more than one order of magnitude were observed, a further, dedicated, literature search of incidence and BP estimates in the general population was carried out.

### 2.6. Statistical Methods

Data were reduced by means of contingency tables using RNMR individual disease and disease group codes for frequency calculations. Specific disease names were used to determine the sex ratio and the age at disease onset. Therefore, these features were determined separately for diseases coded with the same group code. The dispersion of regional notifications was described by means of medians and quartiles to minimize the effects of specific situations of selected regions, such as: unusual low sensitivity or unusually high frequencies due to the notification of cases diagnosed in previous years, which were especially likely in regions which activated a registry or adopted a CD record structure shortly before the observation period. The dispersion of the age at symptoms onset was described by means of median and extreme values due to the skewness of the dispersion of the values. Data of frequency for each disease were reported as the yearly average calculated from the total notifications in the three-year observation period. The use of yearly notifications was not considered due to the enhancement of stochastic effects for most diseases.

## 3. Results and Discussion

### 3.1. General Description of the National Database

The National Database was made up of records associated with 195,492 notifications. Its Subset 1, which refers to disease occurrences, was made up of 190,622 records, after excluding 4870 Type 2 duplicate records from the National Database. Subset 1 records regarded 275 individually-coded diseases and 47 disease groups. Records with dates of diagnosis ascertainment and certification in the period 2012–2014 were 26,870 and 36,292, respectively. Both the National Database and its Subset 1 contain 6607 Type 3 duplicate records related to cases with multiple diagnoses (3.5% total records of Subset 1). These duplicates may represent cases with multiple diseases, second opinions, and revised or refined diagnoses. Since it was not possible to distinguish among these different possibilities, no selection was applied at this time. Most of these records referred to group-coded pathologies and the differing diagnoses pertained to the same code. In 1855 records, referring to 925 cases, the diagnoses pointed at diseases belonging to different chapters of the ICD9-CM.

### 3.2. Data Quality: Missing, Inaccurate or Inconsistent Values

The disease onset date was missing or inaccurate in 26% of records of the National Database. The limited completeness of the onset date prevented the use of this variable to study the incidence of the monitored RD in the whole database, regardless of the notification practice. Due to the importance of this variable, a dedicated analysis was carried out to characterize the incomplete records. The study considered both the notifying region and the disease. Figure S1 shows the distribution of the notified diseases in completeness classes of the onset date per notifying region. Data presented also includes the conventional dates for unknown dates and asymptomatic cases. It can be observed that the completeness of this date can be very different, even in regions notifying comparable and large fractions of the monitored diseases. Completeness (including conventional values) for each disease or disease group code was, on average, 81.9% (SD: 17.5%; *n* = 317 codes) for AD regions, while it was 54.2 (SD: 23.6; *n* = 303 codes) for CD regions, with a statistically significant difference among the two groups (*p* < 0.001). Therefore, it appears that the completeness of this variable depends, to a considerable extent, on the rules for data access and communication associated with CD or AD notification practices. Since it is not expected that these rules introduce a biased selection of the data for a disease, available data were used for the determination of the disease onset age.

The completeness of the variable indicating the centre making the first ascertainment of the diagnosis was only checked in AD records: this indication was missing in 5% of records. The Fiscal Code and other identifiers were missing in all FVG records, which make up about 1.1% of total records, and in an additional 0.2% of records from other regions. Remarks on the other variables affected less than 1% of records. Table S2 (Supplementary File 1) shows detailed remarks regarding the variables of the National Database.

### 3.3. Data Quality: Selection of the Regions and Reference Period for Frequency Rates Assessment

Table S3 (Supplementary File 1) shows the overall number of RD occurrences per region of residence of the case. The table provides summary evidence that epidemiological analyses of the available data could not rely on a single AD or CD record structure. Consequently, the analyses were carried out for individual regional populations, using the data from the record structure which dominated for each disease.

**Figure 1:**
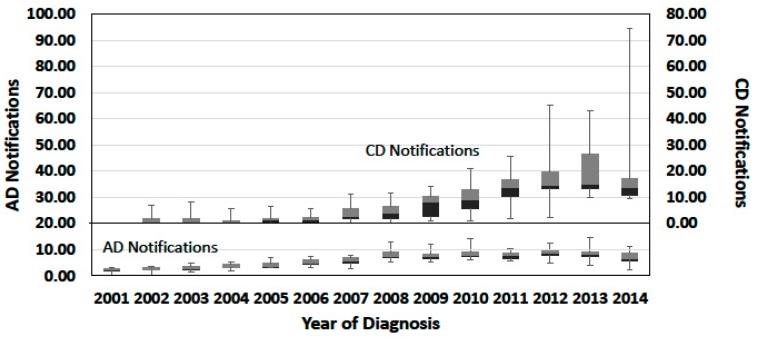
Time course of notifications, by year of diagnosis ascertainment or certification. Dataset: Subset 1; Origin of notifications: Regions using the record structure with the Date of diagnosis Ascertainment (AD) and regions using the record structure with the Date of diagnosis Certification (CD); Selection of records: Records with valid diagnosis ascertainment or certification date; Total records: 115,575 (AD records) and 121,854 (CD records, including those from Lombardia, Piemonte, Sardegna, and Val d’Aosta). Data are expressed as percentage of the total regional notifications (by region of residence) to better compare the progress of AD or CD notifications from regional populations with different dimensions. Absolute numbers of yearly notifications per region of residence are reported in Table S4.

The progress of notifications was studied distinguishing AD from CD notifications, to detect possible differences related to the data collection practice, and the results are summarised in Figure 1. Yearly AD notifications increased from 2001 to 2008 and then remained approximately constant afterwards. The time course of CD notifications showed an increasing trend from 2006 to 2013, when the median of notifications attained the maximum value. The high variance of CD regional notifications is explained by the adoption of the CD record structure by the CD regions at various times till 2011: before 2006, this record structure was only used by Veneto. Moreover, in some regions, the establishment of registries was followed by a peak in the yearly notifications. The variability of the absolute frequencies of regional notifications over time in the periods 2008–2014 and 2012–2014 is presented in Table S4 (Supplementary File 1). Umbria, accounting for about 1.5% of the national population and adopting the CD record structure in 2011, showed by far the highest variability of yearly regional notifications in the period 2012–2014. This period showed the lower variability of both AD and CD notifications with respect to the period 2008–2014. Therefore, it was selected as the reference period for the assessment of RD frequency rates.

### 3.4. Symptoms Onset Age and Sex Distribution

Data of age at disease symptoms onset are reported in Table 1 for selected diseases. Table S5 reports data for all 414 diseases in which it was determined. In total, 108 diseases set on in all cases notified before 18 years of age and 15 showed all occurrences setting on after 18. The comparison with literature data could be carried out for almost three hundred diseases using data available in the ORPHANET portal, which mostly referred to the main life stages where the first symptoms set on (Table S5). There was a general agreement between RNMR and literature data, but for about 12% of compared diseases.

The sex distribution of diseases unevenly frequent between sexes is presented in Table 2. Eleven diseases occurred with a female:male ratio higher than 9:1. Twelve diseases prevailed in males with a female:male ratio lower than 1:9. Data of sex distribution could be compared with data of 65 diseases reported in the ORPHANET data sheets and further literature as having a predominance in either sex. RNMR results showed a good agreement with literature data, except for three diseases (5% comparisons). Table S6 shows the sex distribution of 441 RD reported to RNMR and the comparison with literature data, where available.

### 3.5. Incidence in the General Population and Prevalence in Live Births

Table 3 and Table 4 report, respectively, the rates of diseases (and disease groups) most incident in the general population and most prevalent at birth. National and regional incidence and BP rates of all diseases and disease groups notified to RNMR in Italy are reported in Tables S7 and S8. The collection of data of such a wide range of diseases has been made possible thanks to the sustainability of the registration and, since 2011, full coverage of the population residing in the whole Italian territory. Full population coverage relies on the national network for the diagnosis and care of RD, made up of 247 accredited centres belonging to the public health service [21]. Completeness of case ascertainment relies on mandatory case notification and the possibility for the patients to access RD-specific assistance when their condition is certified by these centres. However, completeness of case ascertainment may depend on the actual need for RD-specific assistance and care. Moreover, the occurrence of diseases and malformations in still births and at termination of pregnancy for foetal anomalies is not recorded. This implies that RNMR data are actually representative of patients requiring care in specialized RD centres.

Median regional incidence and BP rates calculated from CD and from AD collection practices are compared in Tables S9 and S10. Incidence data from the two collection practices showed a Pearson correlation coefficient of 0.78, while the Pearson’s correlation coefficient of BP rates was 0.44. The low correlation of BP rates may be related to the limited population monitored and record selection criteria. For both incidence and BP, the rates calculated from the CD collection practice were, on average, higher than those from the AD collection practice. The reason for such a difference may be related to the different time courses shown by CD and AD notifications (Figure 1), which may involve the certification of cases diagnosed before the adoption of the CD practice.

Incidence data for 34 diseases and BP data for 56 diseases could be compared with data retrieved in the ORPHANET compilations (Tables S11 and S12). Diseases with differences of more than one order of magnitude were subject to a dedicated survey of the literature, which showed that information and data justifying our results were published, except for three and four diseases, respectively, for incidence and BP rates.

## 4. Conclusions

This study determined basic features to assess the reliability and internal consistency of RNMR data. Data of the three-year period 2012–2014 provided information on 275 diseases and 47 disease groups. Moreover, the onset age and sex distribution have been determined for more than 400 individual diseases. This data is mostly representative of patients requiring care in specialized RD centres and is especially suitable to assess the burden of assistance and care by RD-dedicated resources of the public health service.

The external validity of the data collected in the RNMR database was studied by comparing the RD national estimates derived from it with the estimates of frequency in the general population available in the literature. Differences bigger than an order of magnitude were found for 7–8% diseases for which comparisons were possible regarding national incidence and BP. A similar level of agreement with literature data was found regarding the age at disease onset and sex distribution. Therefore, RNMR data is rather consistent with information available in the literature. The remarkable consistency of RNMR data with data of some diseases, which can be traced reliably from hospital records, was also shown in a recent publication [25].

In conclusion, it appears that RNMR results provide basic epidemiological information, which was still lacking for many RD, and overcome the current typical fragmentation of data resulting from the observation of one or a few related diseases in different, selected populations with varied methodologies. In fact, RNMR results compose a unique systematic picture of the occurrence of a wide range of RD in a population as big as about 60 million residents. Although further efforts are still required to achieve the full exploitation of such a complex and extended collection system, this picture represents a novel and sound information basis to improve the assessment of the rare disease burden, and to inform public health policy planning and research prioritization on rare diseases in Italy and beyond.

## Figures and Tables

**Table 1:** Age at symptoms onset of selected rare diseases monitored by RNMR. Dataset: Subset 1; Origin of notifications: all Regions; Record selection: records with diagnosis indicating a specific disease (i.e., excluding disease groups) and at least four records with valid onset date per disease; Total records: 84,859.

RNMR Code	ORPHA Code	Disease	Records (*N*)	Median Onset Age (Years)	Life Stages Reported by ORPHANET [24]
RNG040	1791	Frontofacionasal dysplasia	7	−0.28	Neonatal
RN0490	3447	Weaver syndrome	7	−0.23	Neonatal, Antenatal
RNG030	87	Apert syndrome	13	−0.18	Antenatal, Neonatal
RNG040	2108	Hallermann-Streiff syndrome	9	−0.17	Neonatal, Infancy
RP0040	1915	Fetal alcohol syndrome	56	−0.14	Antenatal, Neonatal
RNG070	313	Lamellar ichthyosis	60	−0.11	Neonatal
RN0400	1540	Jackson-Weiss syndrome	12	−0.10	Neonatal
RNG040	207	Crouzon disease	43	−0.09	Infancy, Neonatal
RN1150	1340	Cardiofaciocutaneous syndrome	54	−0.06	Antenatal, Neonatal
RN0640	1114	Aplasia cutis congenita	14	−0.06	Antenatal, Neonatal
RFG010	141	Canavan disease	6	−0.06	Neonatal, infancy (severe form); childhood (mild form)
RNG040	1452	Cleidocranial dysostosis	7	−0.05	Neonatal
RCG060	352	Galactosemia	58	−0.05	Infancy, Neonatal, Childhood
RN0130	35,737	Morning glory syndrome	10	−0.05	Childhood
RNG050	429	Hypochondroplasia	7	−0.05	Childhood
RP0010	290	Congenital rubella syndrome	31	−0.05	Antenatal, Neonatal
RN0390	380	Greig cephalopolysyndactyly syndrome	15	−0.04	Neonatal
RDG020	326	Factor V deficiency	24	−0.04	All ages
RN0100	708	Peters anomaly	12	−0.04	Infancy, Neonatal
RCG040	238,583	Hyperphenylalaninemia	673	−0.04	Neonatal, Infancy
RN1040	710	Pfeiffer syndrome	10	−0.03	Antenatal, Neonatal
RN1530	500	LEOPARD syndrome	37	−0.03	Childhood
RCG040	407	Non-ketotic hyperglycinemia	6	−0.03	Infancy, Neonatal
RCG060	348	Fructose-1,6-bisphosphatase deficiency	4	−0.03	All ages
RN0510	464	Incontinentia pigmenti	74	−0.03	Neonatal
RN1640	1466	COFS syndrome	6	−0.03	Neonatal, Antenatal
RN1760	912	Zellweger syndrome	13	−0.02	Neonatal
RNG070	461	Recessive X-linked ichthyosis	60	−0.02	Neonatal
RP0060	415,286	Bilirubin encephalopathy	7	−0.02	Neonatal
RCG050	23	Argininosuccinatelyase deficiency	12	−0.02	Neonatal, All ages
RN0910	374	Goldenhar syndrome	187	−0.02	Neonatal, Antenatal
RN0600	312	Epidermolytic ichthyosis	32	−0.02	Neonatal
RDG020	327	Factor VII deficiency	98	−0.02	All ages
RN0990	570	Moebius syndrome	52	−0.02	Neonatal
RN0320	2368	Gastroschisis	57	−0.02	Neonatal, Antenatal
RDG020	328	Factor X deficiency	9	−0.02	All ages
RN0700	280	Wolf-Hirschhorn syndrome	64	−0.02	Neonatal, Antenatal
RN1080	813	Silver-Russell syndrome	85	−0.02	Neonatal, Antenatal
RN0180	1203	Duodenal atresia	75	−0.02	Antenatal, Neonatal, Infancy, Childhood
RN0540	1556	Cutis marmorata telangiectatica congenita	21	−0.02	Neonatal
RCG040	511	Maple syrup urine disease	26	−0.02	Infancy, Neonatal, Childhood
RNG070	634	Netherton syndrome	17	−0.01	Infancy, Neonatal
RN1100	808	Seckel syndrome	11	−0.01	Neonatal, Antenatal
RN1310	739	Prader-Willi syndrome	388	−0.01	Neonatal, Antenatal
RCG040	35	Propionic acidemia	4	−0.01	Infancy, Neonatal
RN0080	1764	Familial dysautonomia	6	−0.01	Neonatal, Antenatal
RDG020	331	Factor XIII deficiency	5	−0.01	All ages
RN0850	138	CHARGE syndrome	116	−0.01	Neonatal
RCG040	293,355	Methylmalonic acidemia	11	−0.01	All ages
RN1170	744	Proteus syndrome	19	−0.01	Infancy
RN0170	1201	Atresia of small intestine	77	−0.01	Neonatal
RN1140	107	Branchio-oto-renal syndrome	35	−0.01	All ages
RN1250	887	VACTERL/VATER association	80	−0.01	Neonatal
RN1410	199	Cornelia de Lange syndrome	74	−0.01	Neonatal, Antenatal
RN0930	392	Holt-Oram syndrome	23	−0.01	Neonatal
RDG020	98,878	Hemophilia A	1209	−0.01	Infancy, Neonatal
RDG020	98,879	Hemophilia B	202	0.00	Infancy, Neonatal
RN1740	899	Walker-Warburg syndrome	6	0.00	Infancy, Neonatal
RN1010	648	Noonan syndrome	459	0.00	Neonatal
RN0430	2911	Poland syndrome	299	0.00	Infancy, Neonatal
RDG020	745	Protein C deficiency	233	0.00	Childhood
RN0200	388	Hirschsprung disease	244	0.00	Infancy, Neonatal
RN0060	2162	Holoprosencephaly	46	0.00	Neonatal, Antenatal
RN1240	857	Townes-Brocks syndrome	8	0.00	All ages
RN1130	1297	Branchio-oculo-facial syndrome	7	0.00	Neonatal
RNG060	1522	Craniometaphyseal dysplasia	11	0.00	Childhood
RN1210	819	Smith-Magenis syndrome	45	0.00	Infancy, Neonatal
RN0890	2053	Freeman-Sheldon syndrome	9	0.00	Neonatal
RCG050	187	Citrullinemia	10	0.00	Neonatal, Adult
RN0360	1465	Coffin-Siris syndrome	13	0.00	Neonatal
RN0410	2311	Jarcho-Levin syndrome	11	0.00	Neonatal, Antenatal
RN0280	950	Acrodysostosis	12	0.00	Neonatal, Antenatal
RN1660	35,125	Epidermal nevus syndrome	15	0.00	Childhood, Adolescent, Adult
RNG040	861	Treacher-Collins syndrome	16	0.00	Neonatal
RN1450	94,068	Congenital spondyloepiphyseal dysplasia	14	0.00	Neonatal
RNG060	289	Ellis-Van Creveld syndrome	8	0.00	Neonatal, Antenatal
RN1590	884	Pallister-Killian syndrome	16	0.00	Neonatal, Antenatal
RN0670	281	Cri du chat syndrome	46	0.00	Neonatal
RN0790	915	Aarskog-Scott syndrome	28	0.00	Childhood
RN0940	2322	Kabuki make-up syndrome	112	0.00	Infancy, Neonatal
RN0040	475	Joubert syndrome	96	0.00	Neonatal, Antenatal
RN1270	904	Williams syndrome	316	0.00	Neonatal, Antenatal
RCG160	567	Di George syndrome	359	0.00	Neonatal
RN1620	783	Rubinstein-Taybi syndrome	63	0.00	All ages
RN1380	110	Bardet-Biedl syndrome	51	0.00	Infancy, Neonatal, Antenatal
RN0660	870	Down syndrome	2283	0.00	Antenatal, Neonatal
RN0680	881	Turner syndrome	947	0.00	Infancy, Neonatal, Antenatal, Childhood
RN0770	3205	Sturge-Weber syndrome	134	0.00	Infancy, Neonatal, Childhood, Adolescent
RN1330	908	Fragile X syndrome	288	0.00	Neonatal, Infancy, Childhood
RN1510	90,308	Klippel-Trénaunay syndrome	170	0.00	Infancy, Childhood, Adolescent
RN0110	77	Aniridia	69	0.00	Infancy, Neonatal
RFG050	83,330	Werdnig-Hoffman disease	13	0.00	Infancy, Neonatal
RCG040	26	Methylmalonic acidemia with homocystinuria	16	0.00	All ages
RN0860	3157	De Morsier syndrome	46	0.00	Infancy, Neonatal
RN1400	191	Cockayne syndrome	6	0.01	All ages
RN1430	220	Denys-Drash syndrome	5	0.02	Infancy, Neonatal
RN0240	2138	True hermaphroditism	17	0.02	Infancy, Neonatal
RN1190	2614	Nail-patella syndrome	33	0.02	Neonatal, Infancy, Childhood
RN0340	974	Adams-Oliver syndrome	11	0.02	Neonatal
RDG020	903	Von Willebrand disease	800	0.02	All ages
RC0180	205	Crigler-Najjar syndrome	27	0.04	Neonatal
RN1730	893	WAGR syndrome	8	0.07	Neonatal
RN0950	98,861	Primary ciliary dyskinesia, Kartagener type	345	0.08	Neonatal, infancy

Note: This table reports pathologies ordered by increasing median age at onset. The number of records is indicated with the only aim of allowing a better assessment of the statistical data presented and cannot be used as an indication of the disease or exemption code frequency.

**Table 2:** Sex distribution of rare diseases monitored by RNMR with the highest relative frequencies in males or females. Dataset: Subset 1; Origin of notifications: all regions; Record selection: records with diagnosis indicating a specific disease (i.e., excluding disease groups) and at least four records per disease; Total records: 119,762.

ORPHA Code	Disease	Records (*N*)	Females (%)	Males (%)	F:M Ratio and Other Literature Information Reported in ORPHANET Data Sheets [24]
1791	Frontofacionasal dysplasia	7	100	0	-
209,981	Iron refractory iron deficiency anemia	4	100	0	-
881	Turner syndrome	1754	99	1	Almost exclusively in F
778	Rett syndrome	603	97	3	Predominant in F
538	Lymphangioleiomyomatosis	180	97	3	Almost exclusively in F
464	Incontinentia pigmenti	95	97	3	20:1
37,202	Interstitial cystitis	1192	95	5	9:1
-	Storage pool deficiency	11	91	9	-
816	Sjögren-Larsson syndrome	93	90	9	-
-	Thrombocytopenic thrombotic purpura-hemolytic-uremic syndrome	10	90	10	-
559	Marinesco-Sjögren syndrome	30	90	10	-
169,615	Idiopathic central precocious puberty	3686	89	11	10:1
98,973	Posterior polymorphous corneal dystrophy	8	88	13	-
79,473	Porphyria variegata	8	88	13	Predominant in F
1297	Branchio-oculo-facial syndrome	8	88	13	-
3143	Schmidt syndrome	72	86	14	-
1452	Cleidocranial dysostosis	7	86	14	-
429	Hypochondroplasia	7	86	14	-
809	Mixed connective tissue disease	1092	86	14	10:1
3287	Takayasu arteritis	346	85	14	Predominant in F
2483	Melkersson-Rosenthal syndrome	25	84	16	-
317	Erythrokeratodermia variabilis	6	83	17	-
98,958	Droplet cornea	17	82	18	-
436	Hypophosphatasia	11	82	18	-
-	Secretion deficiency thrombocytopathy	11	82	18	-
125	Bloom syndrome	5	80	20	-
79,273	Hereditary coproporphyria	5	80	20	Predominant in F
98,974	Fuchs endothelial dystrophy	30	80	20	3–4:1
581	Mucopolysaccharidosis type 3	5	80	20	-
2059	Fryns syndrome	5	80	20	-
65,684	Hirayama disease	5	20	80	1:20
3447	Weaver syndrome	10	20	80	-
98,895	Becker muscular dystrophy	221	19	81	Predominant in M
379	Chronic granulomatous disease	87	18	82	-
221,117	Gerstmann syndrome	6	17	83	-
97,360	Robinow syndrome	6	17	83	-
478	Kallmann syndrome	509	16	84	1:5
101,330	Porphyria cutanea tarda	53	15	85	Predominant in M
-	Opitz syndrome	20	15	85	-
1466	COFS syndrome	7	14	86	-
-	Simpson-Golabi-Behmel syndrome	7	14	86	-
2796	Pachydermoperiostosis	15	13	87	1:7
481	Kennedy disease	51	12	88	-
98,879	Hemophilia B	271	11	89	Predominant in M
223	Nephrogenic diabetes insipidus	66	11	89	-
915	Aarskog-Scott syndrome	38	8	92	Predominant in M
98,878	Hemophilia A	1577	7	93	Predominant in M
98,896	Duchenne muscular dystrophy	258	5	95	Predominant in M
461	Recessive X-linked ichthyosis	65	3	97	Almost exclusively in M
-	Klinefelter syndrome	2071	0	100	-
330	Factor XII deficiency	4	0	100	-
425	Familial hypoalphalipoproteinemia	4	0	100	-
510	Lesch-Nyhan disease	12	0	100	Severe form predominant in M
534	Lowe syndrome	5	0	100	Almost exclusively in M
649	Norrie disease	5	0	100	Almost exclusively in M
580	Mucopolysaccharidosis type 2	27	0	100	Almost exclusively in M
245	Nager syndrome	4	0	100	-

Note: The number of records is indicated with the only aim of allowing a better assessment of the statistical data presented and cannot be used as an indication of the disease or exemption code frequency. Where the percentages in males and females sum to less than 100, the difference represents records with missing sex data.

**Table 3:** National Incidence rates of most incident rare diseases and rare disease groups monitored by RNMR in the Italian population. Yearly average in the three-year period 2012–2014.

ORPHA Code	Disease Name	National Incidence (/Million)
156,071	Keratoconus	25.53
803	Amyotrophic lateral sclerosis	19.15
870	Down syndrome	7.76
169,615	Idiopathic central precocious puberty	7.33
-	Lichen sclerosus	5.92
-	Bullous pemphigoid	4.88
930	Idiopathic achalasia	4.63
117	Behçet disease	4.57
-	Arnold-Chiari malformation	4.19
2932	Chronic inflammatory demyelinating polyneuropathy	3.77
-	Pemphigus vulgaris	3.75
-	Klinefelter syndrome	3.75
397	Giant cell arteritis	3.73
399	Huntington disease	3.38
37,202	Interstitial cystitis	3.19
881	Turner syndrome	2.53
98,249	Ehlers-Danlos syndrome	2.43
774	Hereditary hemorrhagic telangiectasia	2.36
2331	Kawasaki disease	2.08
732	Polymyositis	1.95
733	Familial adenomatous polyposis	1.90
183	Churg-Strauss syndrome	1.88
221	Dermatomyositis	1.87
809	Mixed connective tissue disease	1.80
558	Marfan syndrome	1.79
900	Wegener granulomatosis	1.77
-	Mixed cryoglobulinemia	1.75
-	Idiopathic torsion dystonia	1.46
-	Rheumatic endocarditis	1.36
761	Henoch-Schönlein purpura	1.35
648	Noonan syndrome	1.30
805	Tuberous sclerosis	1.18
908	Fragile X syndrome	1.15
2911	Poland syndrome	1.14
727	Microscopic polyangiitis	1.14
171	Primary sclerosing cholangitis	1.03
904	Williams syndrome	0.98
2073	Narcolepsy	0.97
478	Kallmann syndrome	0.94
96,346	Anorectal malformation	0.93
388	Hirschsprung disease	0.88
790	Retinoblastoma	0.83
91,378	Hereditary angioedema	0.81
104	Leber hereditary optic neuropathy	0.79
739	Prader-Willi syndrome	0.78
70,590	Infantile apnea	0.78
116	Beckwith-Wiedemann syndrome	0.76
550	MELAS	0.73
49,041	Retroperitoneal fibrosis	0.72
3451	West syndrome	0.70
	**Disease Group Name**	
-	Hereditary coagulation defects	25.16
-	Undifferentiated connective tissue syndromes	17.75
-	Hereditary retinic dystrophies	17.60
-	Hereditary anemias	15.04
-	Neurofibromatoses	10.08
-	Muscular dystrophies	8.12
-	Congenital alterations of iron metabolism	8.04
-	Disturbances of aminoacid transport and metabolism	7.92
-	Chromosomal duplication/deficiency syndromes	7.82
-	Hereditary neuropathies	6.61

**Table 4:** National BP Rates of most birth-prevalent rare diseases and rare disease groups monitored by RNMR in the Italian population. Rates in live births of the two-year period 2012–2013.

ORPHA Code	Disease Name	Birth Prevalence (/100,000)
870	Down syndrome	35.00
-	Esophageal atresia and/or Isolated tracheo-esophageal fistula	6.67
96,346	Anorectal malformation	6.37
3451	West syndrome	5.69
388	Hirschsprung disease	4.22
739	Prader-Willi syndrome	3.63
-	Biliary atresia	3.14
805	Tuberous sclerosis	2.84
116	Beckwith-Wiedemann syndrome	2.65
648	Noonan syndrome	2.45
881	Turner syndrome	1.96
1203	Duodenal atresia	1.86
374	Goldenhar syndrome	1.67
1201	Atresia of small intestine	1.67
290	Congenital rubella syndrome	1.47
904	Williams syndrome	1.47
-	Epidermolysis bullosa	1.37
2911	Poland syndrome	1.27
2368	Gastroschisis	1.18
138	CHARGE syndrome	1.18
-	Lissencephaly	1.08
98,861	Primary ciliary dyskinesia, Kartagener type	1.08
464	Incontinentia pigmenti	0.78
887	VACTERL/VATER association	0.78
-	Congenital colobomatous optic disc	0.69
3205	Sturge-Weber syndrome	0.69
-	Alagille syndrome	0.69
2162	Holoprosencephaly	0.59
1896	EEC syndrome	0.59
205	Crigler-Najjar syndrome	0.59
77	Aniridia	0.49
70,590	Infantile apnea	0.49
1915	Fetal alcohol syndrome	0.39
199	Cornelia de Lange syndrome	0.39
908	Fragile X syndrome	0.39
90,308	Klippel-Trénaunay syndrome	0.39
912	Zellweger syndrome	0.29
570	Moebius syndrome	0.29
199,642	Microcephaly	0.29
281	Cri du chat syndrome	0.29
783	Rubinstein-Taybi syndrome	0.29
380	Greig cephalopolysyndactyly syndrome	0.20
280	Wolf-Hirschhorn syndrome	0.20
813	Silver-Russell syndrome	0.20
475	Joubert syndrome	0.20
884	Pallister-Killian syndrome	0.20
3157	De Morsier syndrome	0.20
974	Adams-Oliver syndrome	0.20
726	Alpers syndrome	0.20
435	Ito hypomelanosis	0.20
	**Disease Group Name**	
-	Disturbances of aminoacid transport and metabolism	30.10
-	Congenital craniofacial anomalies	21.57
-	Chromosomal duplication/deficiency syndromes	8.73
-	Disturbances of carbohydrate transport and metabolism, excluded diabetes mellitus	5.39
-	Neurofibromatoses	3.92
-	Congenital chondrodystrophies	3.53
-	Congenital ichthyoses	2.94
-	Other congenital anomalies with intellectual disability	2.84
-	Chromosomal aneuploidy syndromes	2.55
-	Urea cycle disturbances	1.18

## Supplementary Materials

The following are available online at http://www.mdpi.com/1660-4601/15/7/1470/s1: Supplementary File 1: Criteria for data validation; Table S1. Type of national duplicate records and their management; Figure S1. Completeness of disease onset date by notifying region; Table S2. Results of the quality control of data communicated to RNMR; Table S3. Distribution of records by region of residence of the case and validity of diagnosis ascertainment and certification dates; Table S4. Yearly notifications, by region of residence, in different periods of diagnosis ascertainment or confirmation. Table S5: Age at onset of rare diseases reported to RNMR and comparison with literature data. Table S6: Sex distribution of the rare diseases reported to RNMR and comparison with literature data. Table S7: National and regional incidence of rare diseases notified to RNMR during 2012–2014. Table S8: National and regional birth prevalence of rare diseases notified to RNMR (2012–2013). Table S9: Comparison of regional incidence data from AD and CD notifications. Table S10: Comparison of regional birth prevalence data from AD and CD notifications. Table S11: Comparison of national incidence data with literature data. Table S12: Comparison of national birth prevalence data with literature data. References [26,27,28,29,30,31,32,33,34,35,36,37,38,39,40,41,42,43,44,45,46,47] are cited in Supplementary Materials.
