# Supplementary material for: The Occurrence of 275 Rare Diseases and 47 Rare Disease Groups in Italy. Results from the National Registry of Rare Diseases
Source: Int J Environ Res Public Health. 2018 Jul 12;15(7):1470. doi: 10.3390/ijerph15071470 (PMC6068991; doi:10.3390/ijerph15071470)
Supplement: Supplementary file 1 [file ijerph-15-01470-s001.zip › Supplementary file 1.pdf]

Article submitted to International Journal of Environmental Research and Public Health

The Occurrence of 275 Rare Diseases and 47 Rare Disease Groups in Italy. Results from the National Registry of Rare Diseases.

Domenica Taruscio*, Luciano Vittozzi, Adele Rocchetti, Paola Torreri, Luca Ferrari

SUPPLEMENTARY FILE 1

CRITERIA FOR DATA VALIDATION

1. All variables were checked to convert all values indicating missing data or unknown data in the Regional Registries data sets to the value "missing" or "unknown".
2. *National ID Code.* National Fiscal Code is used in Italy to identify the patient including for health service delivery. Patient identifiers communicated to RNMR from RR include the national Fiscal Code. Since several personal details (some letters of the name and surname, sex at birth, birth date with the last two digits of the year, and birthplace) could be inferred from the national Fiscal Code, this was replaced by an automatically generated Encrypted Univocal Patient Code (EUPC) in daily data processing, when the Fiscal Code itself was not strictly necessary. EUPC was generated by CNMR on receiving data from the RR. Due to the algorithm used, the lack of generation of the EUPC indicated an inaccurate or absent Fiscal Code. When EUPC was not generated, the RR were requested to check the data. When the EUPC was not generated nor other personal identifiers were validated, the record was discarded.
3. *Patient Birth Date.* Some RR entered manually the whole birth date (format: gg/mm/aaaa); some used the format mm/gg/aa, others the format gg/mm/aa. In these cases, the birth year in four digits was entered manually as a separate variable. Data were converted to the format gg/mm/aaaa, where necessary using the birth year. Invalid conversion results and dates outside the period 01/01/1915-31/12/2014 were communicated to the RR for a check. Records with confirmed dates subsequent to this period were discarded. Consistency of the birth date with the birth date resulting from the national Fiscal Code was also checked and inconsistencies were signalled to the origin RR.
4. *Patient Birth Place.* Consistency of this data with the birth place resulting from the national Fiscal Code was checked and inconsistencies were fed back to the origin RR. Where this data was missing, the birth region was attributed based on the analysis of the national Fiscal Code.
5. *Patient Place of Residence.* Any data communicated (town, province or region) is converted to the corresponding region. The lack of all these information pieces is communicated to the RR for them to complete data entry.
6. *Disease diagnosed (defined according to exemption codes of MD 279/2001).* At present, RNMR monitors the conditions listed in Annex I of MD 279/2001. This Annex comprises 576 terms: 284 individual diseases, 47 disease groups, 165 examples of diseases or subgroups being included in the disease groups and 80 synonyms, for a total of 331 codes (exemption codes), which give patients the right to exemption from diagnosis and care costs. Synonyms are converted to reference names and codes and the examples of diseases and subgroups are traced to the pertinent disease groups and codes. Some RR have communicated data with disease synonyms and codes other than those listed in MD 279/2001: this data has been collected, checked and converted to the reference names and codes, or traced to the pertinent disease groups, with the collaboration of the involved regional experts. Data of diseases, which were not listed in MD 279/2001, were excluded from the analysis.
7. *Centre notifying the case, with its geographical location and including the centre code*. Values entered for this variable could be obsolete and could have been changed during time due to organizational

reasons. Values were at first updated by means of a correspondence table where historical data collected by RNMR were traced to the updated denominations of Accredited Centres, which were communicated by each Region and Autonomous Province. The updating process considered also the data of the geographic location of the centre to avoid errors due to homonyms. The use of this data was made necessary because the implementation of the national coding system was in progress during most period of data collection and this data was not collected in the usual registration practice. The correspondence table allowed also the attribution of ID Codes, which are now used by the National Health Information System to identify the structures of care of the Public Health System [19]. Historical values which could not be updated with certainty taking into consideration the data available to RNMR, were sent to the RR for them to provide the correct updated denomination and ID Code of the Centre.

8. *Centre ascertaining the diagnosis for the first time, with its geographical location and including the centre code.* The control procedure was the same as for the "Centre notifying the case" except that the correspondence table was built not only with the updated denominations of Accredited Centres, but also with all other structures of care of the Public Health System, as resulting in the documents of the Ministry of Health updated at June 2014 [19]. In case of discrepancy in the updated denominations, the one communicated by the Regions and Autonomous Provinces was preferred.
9. *Geographic location of the Centre*. This data is subject to the same checks indicated under list point 5.
10. *Date of Diagnosis (either first ascertainment or certification of the diagnosis)*. Values which were fed back to RR for them to check were the following: values, which cannot be converted to a valid date expressed in one of the formats gg/mm/aaaa, gg/mm/aa, g/m/aaaa, mm/gg/aa o m/g/aa; values, which are outside the period 01/01/1915-31/12/2014. Confirmed dates following this period cause the record to be discarded from the analysis. Further checks on this date address its consistency with the birth date and the passing date (if present), giving allowance for pre-natal and post-mortem diagnoses. Diagnosis dates are considered not consistent if they precede the birth date by more than 180 days or follow the passing date by more than 60 days.
11. *Disease Onset Date*. Values which are fed back to RR are: missing values; values, which cannot be converted to a valid date expressed in one of the formats gg/mm/aaaa, gg/mm/aa, g/m/aaaa, mm/gg/aa o m/g/aa; values, which are outside the period 01/01/1915-31/12/2014. In case that the year is given with the last two digits only, the year is considered before 2000. Values preceding the birth date by less than 180 days were considered valid. Two conventional date values are used to mark cases which are asymptomatic and cases for which it was not possible to determine the onset date with some degree of certainty.

Table S1. Type of national duplicate records and their management

	**Type 1**	**Type 2**	**Type 3**
**Definition of duplicate type**	Records with the same national ID code, same diagnosis and same notifying Centre	Records with the same national ID code, same diagnosis and different notifying Centre	Records with the same national ID code and different diagnosis (the notifying Centre may be the same or different)
**Presumed reason for duplication**	Same case notified repeatedly by the same Centre[a].	Same case notified by more Centres.	Case with more than one disease or with refined or revised diagnosis.
**Aims of the analysis (record subsets)**	**Record management**		
**Activity of the Centres of the RD National Network (National Database)**	Only the record with the less recent diagnosis ascertainment or certification date is considered[b]	All records are considered	All records are considered
**Epidemiological features of notified RD (Subset 1)**	Only the record with the less recent diagnosis ascertainment or certification date is considered[b]	Only the record with the less recent diagnosis ascertainment or certification date is considered[b]	All records are considered[c]

a: They may be residual records after curation at regional level.
b: The other duplicate records of this type are definitely discarded from the file obtained merging the regional data sets, to obtain the National Database or its Subset 1.
c: Since it was not possible to distinguish the basis of the duplication, no selection was applied at this time.

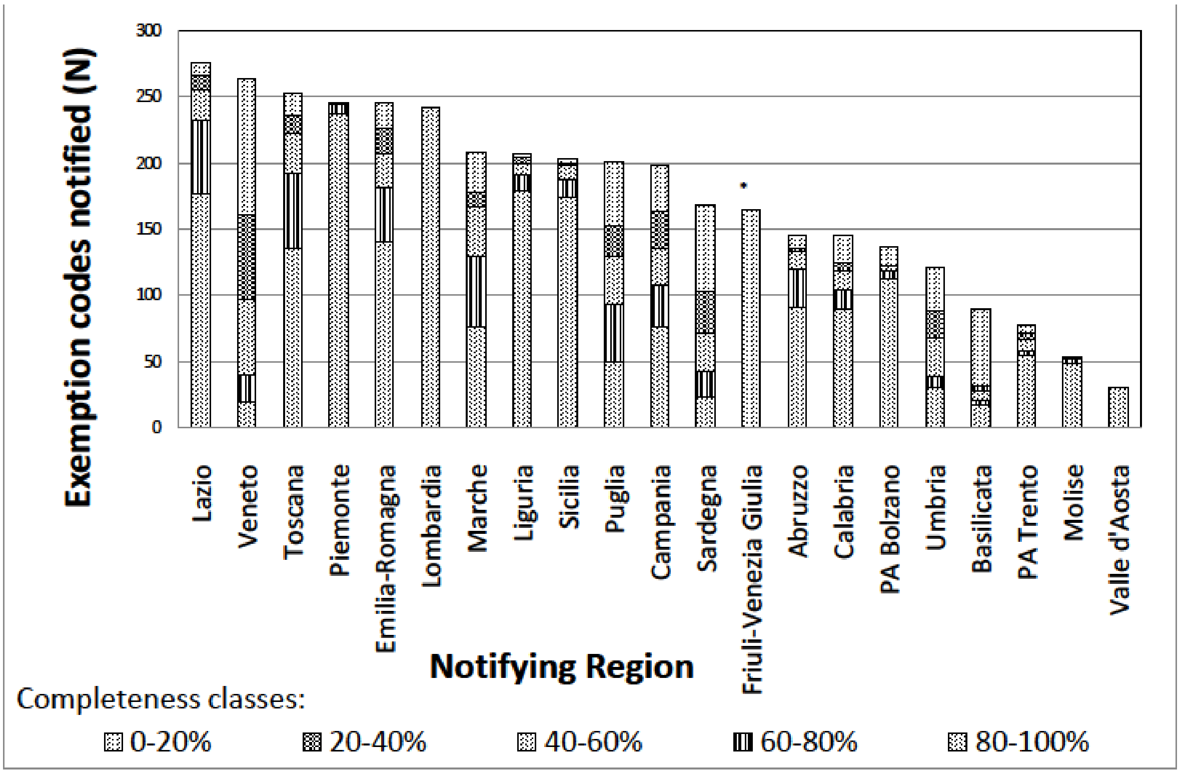

Figure S1. Completeness of disease onset date by notifying region.

The plot indicates the number of disease (and disease group) codes within each completeness class.

Table S2. Results of the quality control of data communicated to RNMR. Dataset: National Database; Origin of notifications: All regions; Selection of records: All records; Total records: 195492

Notifying region / Controls carried out	Abruzzo	Basilicata	Calabria	Campania	Emilia Romagna	Friuli-Venezia Giulia	Lazio	Liguria	Lombardia	Marche	Molise	AP Bolzano	AP Trento	Piemonte e Valle d'Aosta	Puglia	Sardegna	Sicilia	Toscana	Umbria	Veneto	Total
Total records:	1305	614	2830	10382	17761	2283	26663	4909	26282	5212	531	2596	670	18180	7539	4252	7797	25695	1708	28283	**195492**
**Birthdate**																					
**Date before 1915:**	0	1	1	0	8	0	3	0	6	0	0	0	0	6	0	0	0	133	0	18	176
**Inaccurate date:**	0	0	0	0	0	0	0	0	0	146	0	0	0	0	11	0	1	2	0	1	161
**Missing date:**	0	0	0	0	4	0	6	0	0	0	0	0	0	2	0	0	0	0	0	0	12
**Onset date**																					
**Date before 1915:**	0	0	0	0	0	0	0	0	0	0	0	0	0	1	0	0	0	0	0	0	1
**Inaccurate date:**	0	0	0	148	0	0	8410	0	0	319	0	0	0	280	0	0	0	0	0	0	9157
**Date unknown:**	220	285	453	0	0	0	0	0	0	1027	30	0	0	0	0	1900	829	0	0	0	4744
**Asymptomatic cases:**	42	67	161	0	0	0	0	0	0	330	17	0	0	0	0	62	303	2843	0	0	3825
**Missing date:**	0	0	36	2959	4880	2283	0	783	0	0	0	933	119	0	4221	477	0	3693	1049	20130	41563
**Diagnosis ascertainment/ certification date**																					
**Date before 1915:**	0	0	0	0	0	0	1	0	0	0	0	0	0	0	0	0	0	0	0	0	1
**Inaccurate date:**	0	0	0	0	0	0	1	0	0	252	0	0	0	98	0	1	0	6	0	0	358
**Missing date:**	0	0	39	9	10	0	3	0	0	0	0	0	0	125	3	0	3	168	0	0	360
**Personal details**																					
**Missing National ID Code:**	0	0	0	81	270	2283	5	0	0	0	0	0	0	0	0	0	0	0	0	0	2639
**Inaccurate National ID Code:**	0	O	0	4	0	0	36	1	0	0	0	0	0	14	10	5	2	24	2	0	98
**Insufficient residence data:**	0	0	0	8	16	23	130	0	0	0	0	1	0	205	0	0	0	1312	0	0	1695
**Diagnosis**																					
**Missing exemption codes:**	0	0	0	0	0	0	0	0	0	0	0	0	0	0	0	0	0	0	0	0	0

**Missing disease name:**	0	0	0	0	0	0	0	0	0	0	0	0	0	0	0	0	0	0	0	0	0
**Centre ascertaining the diagnosis for the first time**																					
**Centre unknown:**	10	67	28	n.a.	n.a.	n.a.	2527	n.a.	5577	34	18	n.a.	n.a.	690	n.a.	484	14	960	n.a.	n.a.	10409

Birthdate: inaccurate date includes dates with inaccurate or missing birth year and dates expressed in formats other than Excel date format or text formats other than types g/m/aa, g/m/aaaa, m/g/aa, m/g/aaaa.

Date of diagnosis ascertainment/certification: for the regions Piemonte, Valle d'Aosta, Lombardia e Sardegna, which belong to the group of AD regions but communicate both the diagnosis ascertanment and certification dates, the results refer to the control on the diagnosis ascertainment date only.

Personal details: residence data are considered insufficient if neither the commune nor the province nor the region of residence is given.

Centre ascertaining the diagnosis for the first time: the data is considered unknown if it is not given. N.a. indicates that the control is not applicable since the data is not included in the CD record type adopted by the region.

Table S3. Distribution of records by region of residence of the case and validity of diagnosis ascertainment and certification dates. Dataset: Subset 1; Origin of notifications: All regions; Selection of records: All records; Total records: 190622

Residence region	Total records (N)	Records with valid diagnosis ascertainment date (%)	Records with valid diagnosis certification date (%)
Abruzzo	2629	88.3	14.8
Basilicata	1317	72.2	31.1
Calabria	5071	86.9	16.6
Campania	13174	26.9	74.8
Emilia-Romagna	16441	8.2	95.2
Friuli-Venezia Giulia	2876	10.0	93.7
Lazio	20684	96.6	4.4
Liguria	4140	18.7	88.1
Lombardia	23837	93.8	96.4
Marche	5934	89.4	8.4
Molise	761	81.5	20.8
AP Bolzano	2672	2.6	98.4
AP Trento	1667	9.7	95.9
Piemonte	18422	97.2	97.6
Puglia	9629	19.4	84.2
Sardegna	4847	96.1	15.6
Sicilia	10057	89.6	14.6
Toscana	16920	96.0	4.7
Umbria	2935	38.9	62.9
Valle d'Aosta	259	91.1	95.4
Veneto	24760	3.7	98.2
Abroad	61	23.0	98.4
Insufficient residence data	1529	96.3	16.3

Note: Records notified by Piemonte, Val d'Aosta, and Lombardia, which report both ascertainment and certification dates, are counted in both record types.

Table S4. Yearly notifications, by region of residence, in different periods of diagnosis ascertainment or confirmation. Dataset: Subset 1; Origin of notifications: AD Regions (columns 2 and 3) and CD Regions (columns 4 and 5); Selection of records: Records with valid diagnosis ascertainment or certification date; Total records: 115575 (AD notifications) or 121854 (CD Notifications).

	**AD Notifications**		**CD Notifications**	
**Residence region**	**Period 2008-2014**	**Period 2012-2014**	**Period 2008-2014**	**Period 2012-2014**
Abruzzo	213.1 (33.2)	281.3 (16.9)	47.9 (31)	54.7 (31.2)
Basilicata	90.9 (26.8)	101 (33.5)	53.6 (74.1)	90.3 (25.3)
Calabria	274.4 (20.9)	220.7 (5.7)	104.3 (46)	144 (19)
Campania	283.4 (12.9)	297 (10.8)	1383 (118.8)	2797.3 (56.1)
Emilia-Romagna	104.7 (13)	102.7 (19.5)	2045.4 (11.1)	2201.7 (2.6)
Friuli-Venezia Giulia	22 (17)	23.7 (24)	347 (90.6)	560 (66.7)
Lazio	1667.9 (10.8)	1792 (7.5)	116.7 (54.4)	154.3 (37.9)
Liguria	60.4 (17.9)	61.7 (19.3)	516.9 (79.9)	805 (13.9)
Lombardia	1818 (23.3)	1629.3 (33.7)	3191.4 (35.5)	2964 (29.6)
Marche	376.7 (12.4)	397.3 (13.4)	58.9 (26.5)	65.3 (18.4)
Molise	50.4 (19.6)	57 (15.6)	20.6 (76.4)	36.3 (24.8)
AP Bolzano	6.4 (39)	6 (44.1)	370.3 (31.2)	331.3 (15)
AP Trento	15.6 (28.4)	14 (37.8)	204.3 (36.5)	240.7 (9.2)
Piemonte	1491.1 (12.1)	1437.3 (16.5)	2180 (9.9)	2152 (10.5)
Puglia	149.4 (11.4)	155 (17.3)	1127.9 (116.8)	2332 (49.8)
Sardegna	262.6 (36.6)	168.3 (36.7)	97.4 (105.2)	185.7 (55)
Sicilia	740.4 (33.5)	927.7 (22.3)	179.7 (48.7)	216 (26.5)
Toscana	1045.9 (12.9)	1100 (19.4)	102.6 (40.5)	124.3 (22.1)
Umbria	87.6 (12.7)	93 (8.4)	258.4 (195.3)	574.3 (123.4)
Valle d'Aosta	17.9 (36.6)	13.3 (41.3)	30.3 (73.6)	44 (68.8)
Veneto	79.9 (18.7)	78.3 (26.8)	2110 (16.1)	2435 (2.6)

Data are expressed as average of yearly notifications (N). The relative SD, expressed in percent, is reported in brackets. Records notified by Piemonte, Val d’Aosta and Lombardia, which report both dates of certification and ascertainment, are counted in both notification types.
