# Supplementary material for: The Occurrence of 275 Rare Diseases and 47 Rare Disease Groups in Italy. Results from the National Registry of Rare Diseases
Source: Int J Environ Res Public Health. 2018 Jul 12;15(7):1470. doi: 10.3390/ijerph15071470 (PMC6068991; doi:10.3390/ijerph15071470)
Supplement: Supplementary file 1 [file ijerph-15-01470-s001.zip › Table S10.pdf]

Article submitted to International Journal of Environmental Research and Public Health
The Occurrence of 275 Rare Diseases and 47 Rare Disease Groups in Italy. Results from the National Registry of Rare Diseases.
Domenica Taruscio*, Luciano Vittozzi, Adele Rocchetti, Paola Torreri, Luca Ferrari

Table S10. Regional Birth Prevalence data

Note: This table compares the medians of the distributions of regional birth prevalence obtained by two notification practices active in the RNMR. The number of regions making up the distributions is also indicated.

RNMR Code	ORPHA Code	Disease or Disease group	Number of Regions with BP based on CD notifications	Median of regional BP based on CD notifications	Number of Regions with BP based on AD notifications	Median of regional BP based on AD notifications
RB0010	654	Wilms tumor	1	1.2	5	2.0
RB0050	733	Familial adenomatous polyposis	1	1.5	1	1.0
RD0050	379	Chronic granulomatous disease	1	1.5	2	1.8
RF0100	803	Amyotrophic lateral sclerosis	1	1.5	1	1.0
RF0140	3451	West syndrome	6	5.6	7	12.8
RG0040	2331	Kawasaki disease	4	5.0	8	4.1
RN0010		Arnold-Chiari malformation	3	1.3	10	4.7
RN0020	199642	Microcephaly	1	1.3	2	2.0
RN0040	475	Joubert syndrome	1	1.2	1	1.2
RN0050		Lissencephaly	3	1.3	5	1.2
RN0060	2162	Holoprosencephaly	1	1.2	3	1.7
RN0110	77	Aniridia	2	1.8	2	1.1
RN0120		Congenital colobomatous optic disc	3	9.4	1	4.0
RN0160		Esophageal atresia and/or Isolated tracheo-esophageal fistula	7	4.5	8	15.5
RN0170	1201	Atresia of small intestine	4	4.2	4	4.5
RN0180	1203	Duodenal atresia	3	2.6	3	2.3
RN0190	96346	Anorectal malformation	8	10.2	7	8.0
RN0200	388	Hirschsprung disease	6	9.2	3	4.9
RN0210		Biliary atresia	2	7.2	8	3.3
RN0320	2368	Gastroschisis	3	2.6	2	2.2
RN0340	974	Adams-Oliver syndrome	1	1.2	1	1.0
RN0430	2911	Poland syndrome	4	1.3	4	1.4
RN0510	464	Incontinentia pigmenti	1	3.9	4	1.4
RN0570		Epidermolysis bullosa	4	1.4	5	3.0
RN0660	870	Down syndrome	11	19.5	6	60.5
RN0670	281	Cri du chat syndrome	1	1.0	2	0.8
RN0680	881	Turner syndrome	4	3.4	6	3.6
RN0690		Klinefelter syndrome	6	5.6	4	4.5
RN0700	280	Wolf-Hirschhorn syndrome	1	1.5	1	1.0
RN0750	805	Tuberous sclerosis	3	9.0	7	4.3
RN0770	3205	Sturge-Weber syndrome	2	1.8	4	1.3
RN0820	116?	Beckwith-Wiedemann syndrome	3	4.7	5	5.0
RN0850	138	CHARGE syndrome	4	1.2	5	1.7
RN0880	1896	EEC syndrome	1	5.1	4	2.2
RN0910	374	Goldenhar syndrome	6	3.7	4	3.2

RN0950	98861	Primary ciliary dyskinesia, Kartagener type	2	3.4	5	1.4
RN0990	570	Moebius syndrome	2	1.4	1	1.0
RN1010	648	Noonan syndrome	3	2.3	7	3.5
RN1080	813	Silver-Russell syndrome	1	1.3	1	1.0
RN1250	887	VACTERL/VATER association	1	1.5	2	1.3
RN1270	904	Williams syndrome	2	10.0	7	3.0
RN1310	739	Prader-Willi syndrome	3	3.1	9	5.1
RN1330	908	Fragile X syndrome	2	3.1	2	1.5
RN1350		Alagille syndrome	2	5.3	3	3.5
RN1410	199	Cornelia de Lange syndrome	1	1.2	2	3.1
RN1510	90308	Klippel-Trénaunay syndrome	1	3.9	1	0.6
RN1760	912	Zellweger syndrome	1	1.0	2	1.1
RP0040	1915	Fetal alcohol syndrome	1	1.3	1	3.0
RP0050	70590	Infantile apnea	1	4.5	1	1.0
RBG010		Neurofibromatoses	5	4.5	7	3.4
RCG010		Primary hyperaldosteronisms	1	4.5	1	4.0
RCG020		Congenital adrenogenital syndromes	4	3.6	7	3.5
RCG040		Disturbances of aminoacid transport and metabolism	6	34.3	7	26.9
RCG050		Urea cycle disturbances	1	3.5	5	3.9
RCG060		Disturbances of carbohydrate transport and metabolism, excluded diabetes mellitus	2	4.2	7	5.1
RCG070		Congenital alterations of lipoprotein metabolism, excluded: heterozygous familial hypercholesterolhaemia type IIa and IIb; polygenic primary hypercholesterolhaemia; combined familial hypercholesterolhaemia; Hyperlipoproteinhaemia type III	4	7.3	7	3.0
RCG080		Lipid storage disturbances	1	5.3	7	1.4
RCG140		Mucopolysaccharidoses	1	1.2	1	1.5
RCG150		Chronic histiocytoses	1	13.4	2	2.4
RCG160		Primary immunodeficiencies	5	4.3	8	4.1
RDG010		Hereditary anemias	8	26.9	8	5.5
RDG020		Hereditary coagulation defects	5	9.4	6	4.9
RDG040		Primary hereditary thrombocytopenias	1	0.6	1	1.4
RFG010		Leucodystrophies	2	2.7	2	1.1
RFG030		Gangliosidoses	4	1.2	3	2.0
RFG050		Spinal muscular atrophies	5	3.1	6	4.6
RFG060		Hereditary neuropathies	2	1.4	3	1.4
RFG070		Hereditary congenital miopathies	1	1.2	4	1.3
RFG080		Muscular dystrophies	2	4.9	7	3.0
RFG090		Miotonic dystrophies	5	1.3	2	2.4
RFG110		Hereditary retinic dystrophies	1	2.3	3	3.3
RNG010		Pseudohermaphroditisms	2	1.2	3	4.2
RNG020		Multiple congenital arthrogryposes	2	1.4	3	4.0
RNG040		Congenital craniofacial anomalies	4	57.1	8	7.8
RNG050		Congenital chondrodystrophies	6	3.0	5	4.0
RNG060		Congenital osteodystrophies	3	3.9	6	7.3
RNG070		Congenital ichthyoses	4	2.5	8	6.6
RNG080		Chromosomal aneuploidy syndromes	4	4.2	6	3.2
RNG090		Chromosomal duplication/deficiency syndromes	6	16.0	9	8.4

RNG100	Other congenital anomalies with intellectual disability	1	1.2	9	4.3
	**Mean**		5.17		4.30
	**SD**		7.99		7.29
	**Pearson correlation coefficient**		0.44		0
