# Supplementary material for: The Occurrence of 275 Rare Diseases and 47 Rare Disease Groups in Italy. Results from the National Registry of Rare Diseases
Source: Int J Environ Res Public Health. 2018 Jul 12;15(7):1470. doi: 10.3390/ijerph15071470 (PMC6068991; doi:10.3390/ijerph15071470)
Supplement: Supplementary file 1 [file ijerph-15-01470-s001.zip › Table S11.pdf]

Article submitted to International Journal of Environmental Research and Public Health
The Occurrence of 275 Rare Diseases and 47 Rare Disease Groups in Italy. Results from the National Registry of Rare Diseases.
Domenica Taruscio*, Luciano Vittozzi, Adele Rocchetti, Paola Torreri, Luca Ferrari

Table S11. Comparison of Incidence data with literature data

Note: This table lists the diseases showing a difference higher than an order of magnitude in the comparison with ORPHANET reported data and the results of an *ad hoc* literature survey.

RNMR Code	ORPHA Code	Disease	Estimate of national incidence (/million)	ORPHANET reported incidence (/million)	Source	Comment
RA0010	548	Hansen disease (Leprosy)	0.03	37	[3]	(1)
RA0020	3452	Whipple disease	0.09	1	[24]	(2)
RA0030	91546	Lyme disease	0.33	219	[3]	(3)
RB0010	654	Wilms tumor	0.66	1.4	[3]	
RB0020	790	Retinoblastoma	0.83	0.50	[3]	
RB0060	538	Lymphangioleiomyomatosis	0.34	0.14	[3]	
RD0020	447	Paroxysmal nocturnal hemoglobinuria	0.24	1.4	[24]	
RD0030	761	Henoch-Schönlein purpura	1.35	1	[24]	
RF0080	399	Huntington disease	3.38	3.8	[3]	
RF0100	803	Amyotrophic lateral sclerosis	19.15	13.5	[3]	
RF0130	2382	Lennox-Gastaut syndrome	0.59	1	[3]	
RG0020	727	Microscopic polyangiitis	1.14	10	[3]	
RG0030	767	Polyarteritis nodosa	0.41	1	[24]	
RG0040	2331	Kawasaki disease	2.08	80	[24]	(4)
RG0050	183	Churg-Strauss syndrome	1.88	1.8	[3]	
RG0060	375	Goodpasture syndrome	0.04	0.8	[3]	
RG0070	900	Wegener granulomatosis	1.77	8.5	[3]	
RG0080	397	Giant cell arteritis	3.73	40	[24]	(5)
RG0090	3287	Takayasu arteritis	0.63	1.9	[3]	
RI0010	930	Idiopathic achalasia	4.63	7.7	[3]	
RI0050	171	Primary sclerosing cholangitis	1.03	6.5	[3]	
RJ0020	49041	Retroperitoneal fibrosis	0.72	3.5	[3]	
RM0010	221	Dermatomyositis	1.87	5.5	[3]	
RM0020	732	Polymyositis	1.95	5.85	[3]	
RM0060	728	Relapsing polychondritis	0.18	3.5	[3]	(6)
RN0110	77	Aniridia	0.11	13	[3]	
RN0220	53035	Caroli disease	0.13	1	[3]	
RN1040	710	Pfeiffer syndrome	0.03	10	[3]	(7)
RN1080	813	Silver-Russell syndrome	0.29	155	[3]	(8)
RN1320	558	Marfan syndrome	1.79	250	[3]	(9)
RN1400	191	Cockayne syndrome	0.00	5	[3]	(10)
RN1480	435	Ito hypomelanosis	0.10	108.5	[3]	(11)
RN1720	3437	Vogt-Koyanagi-Harada disease	0.20	2.5	[24]	(12)
RP0010	290	Congenital rubella syndrome	0.17	0.3	[3]	

Comments

(1) This disease is mainly localized in India and Brasil [24]. The value reported by ORPHANET refers to non-European Countries and very likely it is not a valid reference for the Italian population and RNMR results.

(2) Whipple disease is associated with the presence of *Tropheryma whippelii* and environmental factors may influence the occurrence of the disease. Its incidence has been estimated equal to 0.6/million in the Swiss population [26], a value close to RNMR results.

(3) This disease is a tick-borne bacterial infection caused by *Borrelia burgdorferi* . It is very likely that the sanitary conditions of the non-European populations, to which the published incidence of this disease refers, are not comparable with those of the Italian population.

(4) This value refers to the age bracket 0-5y [24], which in Italy accounts for 4.6% of the population [33]. When this value is referred to the whole Italian population, the resulting incidence would be 3.68, in line with our estimate.

(5) This value refers to the age bracket >50y [24], which acconts for 40,9% of the Italian population [33]. When referred to the whole Italian population, the resulting incidence would be 16,4, differing less than 3 times from our value.

(6) A recent population-based study in UK [34] suggested that previous estimates could be too high and provided an incidence of 0,71 per million, which is rather comparable to ours.

(7) The clinical features of Pfeiffer syndrome, characterized by a prenatal or neonatal onset and poor prognosis [24], suggest that the value reported as incidence is not referred to the general population and could even represent a birth prevalence.

(8) Silver-Russel syndrome is characterized by a clear intrauterine growth retardation accompanied by postnatal growth deficiency [24]. These features make it very unlikely that the data reported here [3] represent the incidence in the general population. Moreover, this data is the mean of the range of values (1-30/100thousands) reported in the ORPHANET data sheets [24]. If these values would represent the birth prevalence, the lower limit would be in line with the birth prevalence reported in the Orphanet compilation as well as with our BP estimate.

(9) The prevalence of Marfan syndrome has been recently determined in the Danish population where possible cases were re-evaluated according to diagnostic criteria revised in 2010 [35]. This study reported a median yearly incidence of 1.9/million, very close to our estimate.

(10) Cockayne syndrome has been the subject of epidemiological studies in Western Europe [36] and Japan [37] and its incidence in births (birth prevalence in our paper) has been estimated at 2.7-2.8/million. A birth prevalence of 2/million is also reported in the same ORPHANET publication [3]. From these birth prevalence data, 2-3 cases would be expected in the Italian newborn population during the 2-year observation period, corresponding to an incidence of less than 0.03 in the general population. A prevalence of 0.4/million in the general Japan population has also been estimated [37]. Applying an average lifetime of 12 years [24], an incidence of 0.03/million would be expected. These comparisons make the lack of observation of Cockayne cases by RNMR rather compatible with literature data.

(11) An incidence corresponding to the value reported by the ORPHANET compilation was calculated from the disease frequency among unselected patients in a children hospital [38]. Very likely the reported value is not representative of the general population.

(12) The Vogt-Koyanagi-Harada disease affects individuals with darker pigmentation and those of Asian, Hispanic, or Native American origin [24]. This preferential distribution may explain the lower incidence of the disease found in the Italian population, with respect to the ORPHANET data attributed to non-European populations.

References

[3] ORPHANET. Prevalence and incidence of rare diseases: Bibliographic data Prevalence, incidence or number of published cases listed by diseases (in alphabetical order). ORPHANET Report Series N.1, June 2017. Available online: http://www.orpha.net/orphacom/cahiers/docs/GB/Prevalence_of_rare_diseases_by_alphabetical_list.pdf (Accessed on 15 July 2017).

[24] ORPHANET. Disease data sheets. Available online: http://www.orpha.net/consor/cgi-bin/Disease_Search_Simple.php?lng=EN (Accessed on 3 April 2017).

[26] Dutly F., Altwegg M. Whipple’s Disease and “Tropheryma whippelii”. Clin Microbiol Rev 2001, 14, 561–583.

[33] ISTAT. Popolazione per età, sesso e stato civile 2013. Available online: http://www.tuttitalia.it/statistiche/popolazione-eta-sesso-stato-civile-2013/ (Accessed on 10 November 2017).

[34] Hazra N., Dregan A., Charlton J., Gulliford M.C., D'Cruz D.P. Incidence and mortality of relapsing polychondritis in the UK: a population-based cohort study. Rheumatology (Oxford). 2015, 54, 2181-7. doi: 10.1093/rheumatology/kev240. Epub 2015 Jul 17.

[35] Groth K.A., Hove H., Kyhl K., Folkestad L., Gaustadnes M., Vejlstrup N., Stochholm K., Østergaard J.R., Andersen N.H., Gravholt C.H. Prevalence, incidence, and age at diagnosis in Marfan Syndrome. Orphanet J Rare Dis 2015, 10, 153. doi: 10.1186/s13023-015-0369-8.

[36] Kleijer W.J., Laugel V., Berneburg M., Nardo T., Fawcett H., Gratchev A., Jaspers N.G., Sarasin A., Stefanini M., Lehmann A.R. Incidence of DNA repair deficiency disorders in western Europe: Xeroderma pigmentosum, Cockayne syndrome and trichothiodystrophy. DNA

[37] Kubota M., Ohta S., Ando A., Koyama A., Terashima H., Kashii H., Hoshino H., Sugita K., Hayashi M. Nationwide survey of Cockayne syndrome in Japan: Incidence, clinical course and prognosis. Pediatr Int 2015, 57, 339-347. doi: 10.1111/ped.12635.

[38] Pascual-Castroviejo I., Lopez-Rodriguez L., de la Cruz Medina M., Salamanca-Maesso C., Roche Herrero C. Hypomelanosis of Ito: neurological complications in 34 cases. Canad J Neurol Sci 1988, 15, 124-129.
