# Supplementary material for: The Occurrence of 275 Rare Diseases and 47 Rare Disease Groups in Italy. Results from the National Registry of Rare Diseases
Source: Int J Environ Res Public Health. 2018 Jul 12;15(7):1470. doi: 10.3390/ijerph15071470 (PMC6068991; doi:10.3390/ijerph15071470)
Supplement: Supplementary file 1 [file ijerph-15-01470-s001.zip › Table S12.pdf]

Article submitted to International Journal of Environmental Research and Public Health
The Occurrence of 275 Rare Diseases and 47 Rare Disease Groups in Italy. Results from the National Registry of Rare Diseases.
Domenica Taruscio*, Luciano Vittozzi, Adele Rocchetti, Paola Torreri, Luca Ferrari

Table S12. Comparison of birth prevalence data with literature data

Note: This table lists the diseases showing a difference higher than an order of magnitude in the comparison with ORPHANET reported data and the results of an ad hoc literature survey. Diseases with a median onset age higher than 6 months have been excluded from the comparison with literature data, since the number of cases diagnosed in the first year of life, condition required for the selection of records, will become less and less exhaustive.

RNMR Code	ORPHA Code	Disease	Estimated National Birth Prevalence (/100 000)	Orphanet reported Birth Prevalence (/100 000)	Source	Comment
RC0050	508	Leprechaunism	0.1	0.10	[24]	
RC0180	205	Crigler-Najjar syndrome	0.6	0.10	[3]	
RF0010	726	Alpers syndrome	0.2	0.07	[3]	
RF0140	3451	West syndrome	5.7	3.70	[3]	
RN0040	475	Joubert syndrome	0.2	1.13	[3]	
RN0060	2162	Holoprosencephaly	0.6	13.40	[3]	(1)
RN0170	1201	Atresia of small intestine	1.7	16.00	[3]	
RN0180	1203	Duodenal atresia	1.9	9.00	[3]	
RN0200	388	Hirschsprung disease	4.2	10.90	[3]	
RN0240	2138	True hermaphroditism	0.0	2.50	[3]	(2)
RN0260	294975	Phocomelia	0.0	0.62	[3]	(3)
RN0300	3027	Caudal regression sequence	0.0	1.00	[24]	(4)
RN0320	2368	Gastroschisis	1.2	23.70	[3]	(5)
RN0350	192	Coffin-Lowry syndrome	0.0	1.00	[24]	
RN0390	380	Greig cephalopolysyndactyly syndrome	0.2	0.10	[24]	
RN0430	2911	Poland syndrome	1.3	1.50	[3]	
RN0440	3169	Sirenomelia	0.0	0.98	[3]	(6)
RN0510	464	Incontinentia pigmenti	0.8	1.20	[3]	
RN0640	1114	Aplasia cutis congenita	0.1	10.00	[3]	
RN0660	870	Down syndrome	35.0	95.00	[3]	
RN0670	281	Cri du chat syndrome	0.3	2.00	[24]	
RN0680	881	Turner syndrome	2.0	5.50	[3]	
RN0700	280	Wolf-Hirschhorn syndrome	0.2	2.00	[3]	(7)
RN0750	805	Tuberous sclerosis	2.8	10.00	[3]	
RN0770	3205	Sturge-Weber syndrome	0.7	3.50	[3]	
RN0790	915	Aarskog-Scott syndrome	0.1	0.50	[3]	
RN0850	138	CHARGE syndrome	1.2	6.50	[3]	
RN0860	3157	De Morsier syndrome	0.2	10.00	[3]	(8)
RN0870	235	Dubowitz syndrome	0.0	0.20	[3]	(9)
RN0880	1896	EEC syndrome	0.6	1.11	[3]	
RN0900	2059	Fryns syndrome	0.1	7.00	[3]	(10)

RN0910	374	Goldenhar syndrome	1.7	2.90	[3]	
RN0930	392	Holt-Oram syndrome	0.0	0.70	[3]	(11)
RN0950	98861	Primary ciliary dyskinesia, Kartagener type	1.1	3.00	[24]	
RN1010	648	Noonan syndrome	2.5	40.00	[24]	(12)
RN1080	813	Silver-Russell syndrome	0.2	0.70	[3]	
RN1100	808	Seckel syndrome	0.0	0.20	[3]	(9)
RN1170	744	Proteus syndrome	0.1	0.10	[24]	
RN1190	2614	Nail-patella syndrome	0.0	0.20	[3]	(9)
RN1250	887	VACTERL/VATER association	0.8	6.25	[3]	
RN1270	904	Williams syndrome	1.5	10.80	[3]	
RN1300	72	Angelman syndrome	0.2	1.30	[3]	
RN1310	739	Prader-Willi syndrome	3.6	3.10	[3]	
RN1330	908	Fragile X syndrome	0.4	2.40	[3]	
RN1380	110	Bardet-Biedl syndrome	0.0	0.50	[3]	
RN1400	191	Cockayne syndrome	0.0	0.20	[3]	(9)
RN1410	199	Cornelia de Lange syndrome	0.4	1.30	[3]	
RN1450	94068	Congenital spondyloepiphyseal dysplasia	0.0	1.00	[3]	(13)
RN1460	2052	Fraser syndrome	0.1	0.20	[3]	
RN1590	884	Pallister-Killian syndrome	0.2	4.00	[3]	(14)
RN1620	783	Rubinstein-Taybi syndrome	0.3	0.70	[3]	
RN1730	893	WAGR syndrome	0.0	0.20	[3]	(9)
RN1740	899	Walker-Warburg syndrome	0.0	1.65	[3]	
RN1760	912	Zellweger syndrome	0.3	2.00	[24]	
RP0010	290	Congenital rubella syndrome	1.5	0.35	[3]	
RP0040	1915	Fetal alcohol syndrome	0.4	1.60	[3]	

Comments

(1) The average BP calculated from all EUROCAT full member registries in the period 2010-2014 is 0.17-0.23/100000 live births [39], in line with our estimate.

(2) About 15% individuals are observed at birth with ambiguous genitalia [40]. They may be the only one reported to RNMR in the notification conditions selected for our estimates. Correcting the Orphanet reported value taking into consideration the fraction of cases symptomatic at birth, the resulting value (0.37) would justify the absence of findings in the observed newborn population.

(3) Severe skeletal dysplasias are identified prenatally by ultrasound. Affected pregnancies are often terminated by choice and prevalence in live births is much lower than in total pregnancies. The ORPHANET reported value might have been referred to total pregnancies and not to livebirths.

(4) Orphanet reported value is referred to pregnancies and not to live births. BP values in live births are lower than in pregnancies when the disease cause prenatal death or if termination of pregnancy by choice is possible.

(5) The average 2010-2014 calculated from all EUROCAT full member registries is 2.26-2.27/100000 live births [39], in line with our estimate.

(6) The Orphanet portal states that most cases are stillborn, or die during, or shortly after, birth [24]. It is possible that prenatal diagnosis results in termination of pregnancy by choice, so that prevalence in live births is much lower than in total pregnancies. The Orphanet reported value might have been referred to total pregnancies and not to livebirths.

(7) A UK population-based study (1989-1998) found a BP of 1/95896, i.e. 1.04/100thousand [41].

(8) A study in South-Eastern Hungary found a BP equal to 1.1/100thousand [42].

(9) Due to the limited newborn population monitored in the whole observation period (about 1 million live new-borns all over Italy), the lack of observations can be considered justified by the published BP of 0.2/100000

(10) At least some major malformations associated with the disease are noted during pregnancies. Survival beyond the neonatal period is uncommon [24]. It is possible that the ORPHANET reported value refers to total pregnancies and not to live births.

(11) This syndrome showed regional variation in Europe in the range 0.2-2.4/100thousand births. Moreover, the 1990-2011 study showed a significant mean BP decrease from 1.1 to 0.4 between the first and second half observation period [43]. The lower values (range and mean) justify the lack of findings in the observed new-born population.

(12) Noonan syndrome is often difficult to recognize in the newborn [44]. Therefore it is likely that the cases are not diagnosed in the notification conditions selected in our study.

(13) A French study found a BP of 0,94/100thousands pregnancies [45]. Severe skeletal dysplasias are identified prenatally by ultrasound. Affected pregnancies are often terminated by choice [46] and prevalence in live births is much lower than in total pregnancies.

(14) A UK population-based study found a BP of 0.51/100thousand in live births [47].

References

[3] ORPHANET. Prevalence and incidence of rare diseases: Bibliographic data Prevalence, incidence or number of published cases listed by diseases (in alphabetical order). ORPHANET Report Series N.1, June 2017. Available online: http://www.orpha.net/orphacom/cahiers/docs/GB/Prevalence_of_rare_diseases_by_alphabetical_list.pdf (Accessed on 15 July 2017).

[24] ORPHANET. Disease data sheets. Available online: http://www.orpha.net/consor/cgi-bin/Disease_Search_Simple.php?lng=EN (Accessed on 3 April 2017).

[39] EUROCAT. Prevalence Tables. Available on-line at: http://www.eurocat-network.eu/accessprevalencedata/prevalencetables (Accessed on 10 November 2017).

[40] Zenteno-Ruiz J.C., Kofman-Alfaro S., Méndez J.P. 46,XX sex reversal. Arch Med Res 2001, 32, 559–566.

[41] Shannon N.L., Maltby E.L., Rigby A.S., Quarrell O.W.J. An epidemiological study of Wolf-Hirschhorn syndrome: life expectancy and cause of mortality. J Med Genet 2001, 38, 674–679.

[42] Szabó N.Z. Epidemiology of central nervous system malformations in South-Eastern Hungary. A Thesis for the Degree of Doctor of Philosophy, Department of Paediatrics, Faculty of Medicine, University of Szeged (2012) Available online at: http://doktori.bibl.u-szeged.hu/1485/1/SZAB%C3%93_N%C3%93RA_PhD_ertekezes.pdf (Accessed 15 November 2017).

[43] Barisic I., Boban L., Greenlees R., Garne E., Wellesley D., Calzolari E., Addor M.-C., Arriola L., Bergman J.E.H., Braz P., Budd J.L.S., Gatt M., Haeusler M., Khoshnood B., Klungsoyr K., McDonnell B., Nelen V., Pierini ., Queisser-Wahrendorf A., Rankin J., Rissmann A., Rounding C., Tucker D., Verellen-Dumoulin C., Dolk H. Holt Oram syndrome: a registry-based study in Europe. Orphanet J Rare Dis 2014, 9, 156 Available on-line at: https://ojrd.biomedcentral.com/track/pdf/10.1186/s13023-014-0156-y?site=ojrd.biomedcentral.com (Accessed November 2017)

[44] Noonan J.A. Noonan syndrome revisited. J Pediatr 1999, 135, 667-668

[45] Stoll, C., Dott, B., Roth, M.-P. and Alembik, Y. Birth prevalence rates of skeletal dysplasias. Clin Genet, 1989, 35, 88–92. doi:10.1111/j.1399-0004.1989.tb02912.x

[46] Holmes L.B. Common Malformations. Oxford University Press (UK) 2012, p. 277-283.

[47] Blyth, M., Maloney, V., Beal, S., Collinson, M., Huang, S., Crolla, J., Temple, I. K., Baralle, D. Pallister-Killian syndrome: a study of 22 British patients. J Med Genet 2015, 52, 454-464,
