# Supplementary material for: The Occurrence of 275 Rare Diseases and 47 Rare Disease Groups in Italy. Results from the National Registry of Rare Diseases
Source: Int J Environ Res Public Health. 2018 Jul 12;15(7):1470. doi: 10.3390/ijerph15071470 (PMC6068991; doi:10.3390/ijerph15071470)
Supplement: Supplementary file 1 [file ijerph-15-01470-s001.zip › Table S5.pdf]

Article submitted to International Journal of Environmental Research and Public Health
The Occurrence of 275 Rare Diseases and 47 Rare Disease Groups in Italy. Results from the National Registry of Rare Diseases.
Domenica Taruscio*, Luciano Vittozzi, Adele Rocchetti, Paola Torreri, Luca Ferrari

Table S5. Age at onset of rare diseases reported to RNMR

Record source: Subset 1; **Notifying regions**: all; **Record selection**: records with diagnosis indicating a specific disease (i.e.: excluding disease groups) and at least 4 records with valid onset date per disease; **Total records**: 84859.
Note: This table reports pathologies ordered by increasing median age at onset. The number of records is indicated with the only aim of allowing a better assessment of the statistical data presented and cannot be used as an indication of the disease or exemption code frequency.

RNMR Code	ORPHA Code	Disease	Records (N)	Median age (years)	Minimum age (years)	Maximum age (years)	Life stages or age ranges of usual disease onset reported by ORPHANET data sheets [24], if not otherwise referenced
RNG040	1791	Frontofacionasal dysplasia	7	-0.28	-0.39	0.00	Neonatal
RN0490	3447	Weaver syndrome	7	-0.23	-0.39	-0.04	Neonatal, Antenatal
RNG030	87	Apert syndrome	13	-0.18	-0.48	0.75	Antenatal, Neonatal
RNG040	2108	Hallermann-Streiff syndrome	9	-0.17	-0.48	1.40	Neonatal, Infancy
RP0040	1915	Fetal alcohol syndrome	56	-0.14	-0.47	13.96	Antenatal, Neonatal
RNG040		Oculomaxillofacial dysostosis	20	-0.11	-0.47	4.85	
RI0070	2290	Microvillus inclusion disease (MVII	6	-0.11	-0.35	0.04	
RNG070	313	Lamellar ichthyosis	60	-0.11	-0.49	5.73	Neonatal
RN0400	1540	Jackson-Weiss syndrome	12	-0.10	-0.46	0.13	Neonatal
RNG040	207	Crouzon disease	43	-0.09	-0.49	43.43	Infancy, Neonatal
RN0530		Keratosis follicularis spinulosa dec	6	-0.07	-0.39	2.70	
RNG040		Pierre Robin syndrome	50	-0.07	-0.49	1.16	
RN1150	1340	Cardiofaciocutaneous syndrome	54	-0.06	-0.44	3.63	Antenatal, Neonatal
RN0640	1114	Aplasia cutis congenita	14	-0.06	-0.35	23.46	Antenatal, Neonatal
RFG010	141	Canavan disease	6	-0.06	-0.14	0.41	neonatal, infancy (severe form); childhood (mild form)
RNG040	1452	Cleidocranial dysostosis	7	-0.05	-0.08	9.93	Neonatal
RCG060	352	Galactosemia	58	-0.05	-0.48	1.89	Infancy, Neonatal, Childhood
RN0130	35737	Morning glory syndrome	10	-0.05	-0.08	0.65	Childhood

RN1670		Multiple pterygium syndrome	6	-0.05	-0.43	8.79	
RNG050	429	Hypochondroplasia	7	-0.05	-0.05	0.03	Childhood
RP0010	290	Congenital rubella syndrome	31	-0.05	-0.08	0.00	Antenatal, Neonatal
RN1020		Opitz syndrome	17	-0.05	-0.47	12.30	
RN0390	380	Greig cephalopolysyndactyly synd	15	-0.04	-0.48	1.92	Neonatal
RNG070		Autosomal recessive congenital icl	17	-0.04	-0.08	0.10	
RDG020	326	Factor V deficiency	24	-0.04	-0.06	38.84	All ages
RN0500		Cutis laxa	10	-0.04	-0.25	63.35	
RN0090		Axenfeld-Rieger anomaly	14	-0.04	-0.38	3.57	
RN1200		Smith-Lemli-Opitz syndrome type	29	-0.04	-0.47	25.20	
RN0100	708	Peters anomaly	12	-0.04	-0.47	0.05	Infancy, Neonatal
RCG040	238583	Hyperphenylalaninemia	673	-0.04	-0.08	0.04	Neonatal, Infancy
RN1040	710	Pfeiffer syndrome	10	-0.03	-0.49	0.13	Antenatal, Neonatal
RN1530	500	LEOPARD syndrome	37	-0.03	-0.48	24.41	Childhood
RCG040		Oculocutaneous albinism	131	-0.03	-0.49	36.64	
RCG040	407	Non-ketotic hyperglycinemia	6	-0.03	-0.06	-0.01	Infancy, Neonatal
RCG060	348	Fructose-1,6-bisphosphatase defic	4	-0.03	-0.05	2.68	All ages
RN0510	464	Incontinentia pigmenti	74	-0.03	-0.45	34.94	Neonatal
RN1640	1466	COFS syndrome	6	-0.03	-0.17	1.48	Neonatal, Antenatal
RC0030		Reifenstein syndrome	28	-0.03	-0.42	54.33	
RN1760	912	Zellweger syndrome	13	-0.02	-0.47	0.30	Neonatal
RCG040		Tyrosinemia	12	-0.02	-0.06	2.88	
RNG070	461	Recessive X-linked ichthyosis	60	-0.02	-0.48	9.98	Neonatal
RN0570		Epidermolysis bullosa	302	-0.02	-0.49	80.16	
RP0060	415286	Bilirubin encephalopathy	7	-0.02	-0.34	0.28	Neonatal
RCG050	23	Argininosuccinatelyase deficiency	12	-0.02	-0.07	3.98	Neonatal, All ages
RN0910	374	Goldenhar syndrome	187	-0.02	-0.49	32.29	Neonatal, Antenatal
RN0600	312	Epidermolytic ichthyosis	32	-0.02	-0.38	21.40	Neonatal
RN0190	96346	Anorectal malformation	361	-0.02	-0.49	29.01	
RDG020	327	Factor VII deficiency	98	-0.02	-0.08	82.63	All ages
RN0990	570	Moebius syndrome	52	-0.02	-0.48	0.08	Neonatal
RN0320	2368	Gastroschisis	57	-0.02	-0.45	3.55	Neonatal, Antenatal
RDG020	328	Factor X deficiency	9	-0.02	-0.07	14.14	All ages

RN0120		Congenital colobomatous optic dis	78	-0.02	-0.46	27.49	
RN0700	280	Wolf-Hirschhorn syndrome	64	-0.02	-0.46	0.77	Neonatal, Antenatal
RN0870	235	Dubowitz syndrome	8	-0.02	-0.20	1.75	
RN1080	813	Silver-Russell syndrome	85	-0.02	-0.39	10.00	Neonatal, Antenatal
RN0180	1203	Duodenal atresia	75	-0.02	-0.41	15.45	Antenatal, Neonatal, Infancy, Childhood
RN1050		Rieger syndrome	9	-0.02	-0.18	8.68	
RN0540	1556	Cutis marmorata telangiectatica co	21	-0.02	-0.44	26.77	Neonatal
RCG040	511	Maple syrup urine disease	26	-0.02	-0.48	6.93	Infancy, Neonatal, Childhood
RNG070	634	Netherton syndrome	17	-0.01	-0.38	0.05	Infancy, Neonatal
RN1100	808	Seckel syndrome	11	-0.01	-0.30	1.41	Neonatal, Antenatal
RN1310	739	Prader-Willi syndrome	388	-0.01	-0.48	50.46	Neonatal, Antenatal
RCG040	35	Propionic acidemia	4	-0.01	-0.02	0.00	Infancy, Neonatal
RN0160		Esophageal atresia and/or Isolatec	242	-0.01	-0.49	8.98	
RN0080	1764	Familial dysautonomia	6	-0.01	-0.24	65.98	Neonatal, Antenatal
RN0740		Ivemark syndrome	4	-0.01	-0.03	0.00	
RDG020	331	Factor XIII deficiency	5	-0.01	-0.07	3.85	All ages
RN0850	138	CHARGE syndrome	116	-0.01	-0.49	40.33	Neonatal
RCG040	293355	Methylmalonic acidemia	11	-0.01	-0.07	2.09	All ages
RN1170	744	Proteus syndrome	19	-0.01	-0.19	18.46	Infancy
RN0170	1201	Atresia of small intestine	77	-0.01	-0.41	41.23	Neonatal
RNG040		Primary craniosynostosis	432	-0.01	-0.49	28.09	
RN1140	107	Branchio-oto-renal syndrome	35	-0.01	-0.45	38.75	All ages
RN0820		Beckwith-Wiedemann syndrome	256	-0.01	-0.47	44.29	
RN1250	887	VACTERL/VATER association	80	-0.01	-0.49	2.83	Neonatal
RN0030		Cerebellar agenesis	20	-0.01	-0.13	1.63	
RN1410	199	Cornelia de Lange syndrome	74	-0.01	-0.39	6.84	Neonatal, Antenatal
RN0930	392	Holt-Oram syndrome	23	-0.01	-0.31	36.99	Neonatal
RDG020	98878	Hemophilia A	1209	-0.01	-0.49	83.63	Infancy, Neonatal
RDG020	98879	Hemophilia B	202	0.00	-0.23	57.46	Infancy, Neonatal
RN1740	899	Walker-Warburg syndrome	6	0.00	-0.02	0.19	Infancy, Neonatal
RN0260	294975	Phocomelia	43	0.00	-0.30	48.78	
RN1010	648	Noonan syndrome	459	0.00	-0.48	51.32	Neonatal
RN0430	2911	Poland syndrome	299	0.00	-0.49	61.65	Infancy, Neonatal

RDG020	745	Protein C deficiency	233	0.00	-0.08	74.99	Childhood
RN0200	388	Hirschsprung disease	244	0.00	-0.48	27.03	Infancy, Neonatal
RN0060	2162	Holoprosencephaly	46	0.00	-0.45	31.20	Neonatal, Antenatal
RN1240	857	Townes-Brocks syndrome	8	0.00	-0.02	0.33	All ages
RN0210		Biliary atresia	259	0.00	-0.49	1.00	
RN1130	1297	Branchio-oculo-facial syndrome	7	0.00	-0.07	1.10	Neonatal
RNG060	1522	Craniometaphyseal dysplasia	11	0.00	-0.18	2.83	Childhood
RN1210	819	Smith-Magenis syndrome	45	0.00	-0.35	5.26	Infancy, Neonatal
RN0890	2053	Freeman-Sheldon syndrome	9	0.00	-0.28	9.90	Neonatal
RNG050	15?	Achondroplasia	99	0.00	-0.47	10.69	
RCG050	187	Citrullinemia	10	0.00	-0.46	11.06	Neonatal, Adult
RN0360	1465	Coffin-Siris syndrome	13	0.00	-0.31	11.17	Neonatal
RN0410	2311	Jarcho-Levin syndrome	11	0.00	-0.44	11.68	Neonatal, Antenatal
RN0280	950	Acrodysostosis	12	0.00	-0.48	13.03	Neonatal, Antenatal
RN1660	35125	Epidermal nevus syndrome	15	0.00	-0.07	13.54	Childhood, Adolescent, Adult
RFG070		Nemaline myopathy	9	0.00	-0.37	16.12	
RNG040	861	Treacher-Collins syndrome	16	0.00	-0.46	0.06	Neonatal
RN1450	94068	Congenital spondyloepiphyseal dy	14	0.00	-0.47	0.98	Neonatal
RNG060	289	Ellis-Van Creveld syndrome	8	0.00	-0.26	1.96	Neonatal, Antenatal
RN1590	884	Pallister-Killian syndrome	16	0.00	-0.06	2.15	Neonatal, Antenatal
RN0670	281	Cri du chat syndrome	46	0.00	-0.48	3.93	Neonatal
RN0020	199642	Microcephaly	362	0.00	-0.48	19.23	
RN0790	915	Aarskog-Scott syndrome	28	0.00	-0.36	6.00	Childhood
RN0940	2322	Kabuki make-up syndrome	112	0.00	-0.47	21.17	Infancy, Neonatal
RN0040	475	Joubert syndrome	96	0.00	-0.49	27.25	Neonatal, Antenatal
RN1270	904	Williams syndrome	316	0.00	-0.49	30.76	Neonatal, Antenatal
RCG160	567	Di George syndrome	359	0.00	-0.49	31.73	Neonatal
RN1620	783	Rubinstein-Taybi syndrome	63	0.00	-0.48	38.69	All ages
RN1380	110	Bardet-Biedl syndrome	51	0.00	-0.43	39.31	Infancy, Neonatal, Antenatal
RN0660	870	Down syndrome	2283	0.00	-0.47	39.46	Antenatal, Neonatal
RN0680	881	Turner syndrome	947	0.00	-0.48	41.74	Infancy, Neonatal, Antenatal, Childhood
RN0880	1896	EEC syndrome	114	0.00	-0.46	63.11	
RN0770	3205	Sturge-Weber syndrome	134	0.00	-0.43	64.52	Infancy, Neonatal, Childhood, Adolescent

RN1330	908	Fragile X syndrome	288	0.00	-0.37	73.51	Neonatal, Infancy, Childhood
RN1510	90308	Klippel-Trénaunay syndrome	170	0.00	-0.48	34.86	Infancy, Childhood, Adolescent
RN0110	77	Aniridia	69	0.00	-0.48	38.58	Infancy, Neonatal
RCG070		Disorders of fatty acid oxidation a	98	0.00	-0.48	71.76	
RFG050	83330	Werdnig-Hoffman disease	13	0.00	-0.36	0.39	Infancy, Neonatal
RN0290		Camptodactyly	8	0.00	-0.02	8.07	
RCG040	26	Methylmalonic acidemia with hon	16	0.00	-0.08	26.64	All ages
RN0860	3157	De Morsier syndrome	46	0.00	-0.44	15.51	Infancy, Neonatal
RN1350		Alagille syndrome	65	0.01	-0.42	37.60	
RN1400	191	Cockayne syndrome	6	0.01	-0.02	16.85	All ages
RN1430	220	Denys-Drash syndrome	5	0.02	-0.05	16.33	Infancy, Neonatal
RN0240	2138	True hermaphroditism	17	0.02	-0.44	17.08	Infancy, Neonatal
RCG040		Organic acidemias and primary lac	141	0.02	-0.46	53.34	
RN1190	2614	Nail-patella syndrome	33	0.02	-0.07	63.78	Neonatal, Infancy, Childhood
RN0340	974	Adams-Oliver syndrome	11	0.02	-0.06	10.93	Neonatal
RDG020	903	Von Willebrand disease	800	0.02	-0.32	80.98	All ages
RC0180	205	Crigler-Najjar syndrome	27	0.04	-0.04	40.56	Neonatal
RP0050	70590	Infantile apnea	20	0.04	-0.44	15.20	
RN1180	324764	Trichorhinophalangeal syndrome	25	0.05	-0.16	13.51	
RN1730	893	WAGR syndrome	8	0.07	-0.03	0.84	Neonatal
RN0050		Lissencephaly	120	0.07	-0.39	13.85	
RN0950	98861	Primary ciliary dyskinesia, Kartage	345	0.08	-0.49	60.80	Neonatal, infancy
RCG040	534	Lowe syndrome	5	0.09	-0.04	5.36	Neonatal
RN0300	3027	Caudal regression sequence	25	0.09	-0.48	18.17	Infancy, Neonatal
RF0010	726	Alpers syndrome	11	0.09	-0.07	44.72	mostly 2-4, but up to 36
RFG080	97242	Congenital muscular dystrophy	14	0.09	-0.08	2.84	Infancy, Neonatal
RCG060		Congenital disorder of glycosylatic	6	0.10	-0.07	0.43	
RCG040		Glutaric aciduria	4	0.10	-0.08	35.41	
RN1480	435	Ito hypomelanosis	40	0.14	-0.41	29.44	Childhood
RN1370	64	Alström syndrome	6	0.15	-0.07	10.63	Infancy, Neonatal, Childhood, Adolescent, Adult
RCG050	247525	Argininosuccinate synthase deficie	14	0.16	-0.07	39.32	All ages
RFG010	51	Aicardi-Goutieres syndrome	20	0.17	-0.07	1.30	<1m

RN0350	192	Coffin-Lowry syndrome	8	0.18	-0.06	37.60	Neonatal
RDG010		Pyruvate kinase deficiency	19	0.19	-0.08	65.37	
RDG010	124	Blackfan-Diamond anemia	51	0.20	-0.43	34.65	Infancy, Neonatal, Childhood
RC0070	309845	Disorder of zinc metabolism	5	0.22	0.08	0.79	
RN1300	72	Angelman syndrome	201	0.26	-0.49	24.40	Infancy
RNG040	1308	C syndrome	6	0.27	-0.02	3.52	Neonatal, Antenatal
RF0140	3451	West syndrome	506	0.31	-0.49	16.90	3-7 m
RFG010	702	Pelizaeus-Merzbacher disease	16	0.33	-0.08	35.35	All ages
RN0750	805	Tuberous sclerosis	793	0.38	-0.49	70.85	All ages
RN0370	239	Dyggve-Melchior-Clausen disease	4	0.39	-0.03	5.94	Infancy
RCG040		Cystinosis	33	0.41	-0.27	25.16	
RN0560	1775	Dyskeratosis congenita	12	0.43	-0.02	10.80	All ages, usually during childhood
RJ0010	223	Nephrogenic diabetes insipidus	52	0.44	-0.31	65.22	Infancy, Neonatal
RCG120	510	Lesch-Nyhan disease	11	0.50	-0.01	2.99	Infancy
RF0040	778	Rett syndrome	518	0.52	-0.49	20.00	Infancy, Neonatal
RNG060	240	Dyschondrosteosis	28	0.54	-0.01	36.83	Neonatal
RCG140	583	Mucopolysaccharidosis type 6	5	0.56	0.30	0.97	Childhood
RN0610	2092	Focal dermal hypoplasia	12	0.59	-0.45	41.09	Neonatal
RCG140	579	Mucopolysaccharidosis type 1	18	0.60	-0.04	7.00	All ages
RF0030	506	Leigh syndrome	145	0.61	-0.30	39.00	<1
RCG060	469	Hereditary fructose intolerance	51	0.65	-0.21	53.85	All ages
RN1120		Simpson-Golabi-Behmel syndrome	6	0.65	-0.07	8.44	
RB0020	790	Retinoblastoma	539	0.66	-0.47	37.10	<3
RCG010	112	Bartter syndrome	60	0.68	-0.36	64.31	Antenatal, Neonatal, Infancy, Childhood, Adolescent, Adult
RFG050	83418	Spinal muscular atrophy type 2	13	0.71	-0.03	1.49	
RN1600	699	Pearson syndrome	5	0.89	-0.12	3.82	Infancy, Neonatal
RCG160	331223	Iper-Ige syndrome	9	0.96	0.00	17.33	
RN0590	317	Erythrokeratodermia variabilis	6	1.06	-0.41	47.10	neonatal, infancy
RCG160	183669	Agammaglobulinemia	88	1.09	-0.36	40.55	
RDG010		Thalassemias	1848	1.09	-0.46	76.76	
RN0520	910	Xeroderma pigmentosum	20	1.09	-0.40	53.09	All ages
RC0170		Vitamin D-resistent hypophosphat	235	1.14	-0.25	70.78	

RF0130	2382	Lennox-Gastaut syndrome	311	1.24	-0.48	52.04	2-7
RN1220	828	Stickler syndrome	56	1.33	-0.29	53.74	Childhood
RCG070		Carnitine palmitoyltransferase def	10	1.38	-0.07	19.48	
RFG040	100	Ataxia-telangiectasia	35	1.45	-0.45	39.90	Infancy, Childhood
RCG060		Glycogen storage disease	303	1.46	-0.35	62.07	
RD0050	379	Chronic granulomatous disease	63	1.51	-0.42	81.84	Infancy, Childhood, Adolescent, Adult
RCG050	90	Arginase deficiency	4	1.68	-0.04	9.33	Infancy, Childhood
RCG070	14	Abetalipoproteinemia	8	1.69	-0.04	53.65	Infancy, Neonatal
RNG060	666	Osteogenesis imperfecta	388	1.70	-0.47	65.10	All ages
RCG140	581	Mucopolysaccharidosis type 3	12	1.74	-0.08	11.45	Childhood
RCG140	582	Mucopolysaccharidosis type 4	25	1.80	-0.07	9.89	Childhood
RFG040		Congenital ataxia	4	1.89	0.93	18.75	
RG0040	2331	Kawasaki disease	767	1.93	-0.19	51.02	median: 2
RCG070		Beta-oxydation deficiency	19	1.94	-0.07	29.48	
RCG140	580	Mucopolysaccharidosis type 2	23	1.98	-0.06	6.26	Childhood
RFG080	98896	Duchenne muscular dystrophy	215	2.19	-0.30	45.50	Childhood
RDG010	232	Sickle cell anemia	490	2.30	-0.39	60.43	All ages
RDG030	849	Glanzmann thrombasthenia	12	2.33	-0.45	43.26	Infancy, Neonatal
RCG110		Erythropoietic protoporphyria	30	2.45	0.00	37.08	
RFG010	512	Metachromatic leukodystrophy	19	2.56	-0.34	52.75	Childhood, Adolescent, Adult, Infancy
RN0960	163634	Maffucci syndrome	11	2.58	-0.07	51.27	0-5
RFG050	83419	Kugelberg-Welander disease	28	2.67	-0.07	54.72	Childhood, adolescence
RCG060	61	Mannosidosis	7	2.92	-0.06	8.34	Infancy, Neonatal, Childhood
RFG070		Congenital myopathy with fiber ty	7	2.95	-0.03	10.01	
RNG060	562	McCune-Albright syndrome	23	2.96	-0.43	57.33	Childhood
RB0010	654	Wilms tumor	349	2.97	-0.39	18.08	1-5
RP0070		Congenital hepatic fibrosis	36	2.99	-0.08	59.50	
RDG020	98880	Afibrinogenemia	7	3.33	-0.08	61.08	Infancy, Neonatal
RF0060	98261	Progressive myoclonic epilepsy	148	3.39	-0.08	62.70	Neonatal, Infancy, Childhood, Adolescent
RD0040	2686	Cyclic neutropenia	57	3.46	-0.08	56.73	All ages
RN1520	98818	Landau-Kleffner syndrome	17	3.62	0.00	6.65	Childhood
RD0010		Hemolytic-uremic syndrome	338	3.71	0.01	78.26	
RDG020		Dysfibrinogenemia	27	3.82	-0.08	76.65	

RFG040	98	Spastic ataxia of Charlevoix-Sague	14	3.90	1.10	33.63	Infancy, Childhood, Adolescent, Adult
RNG050	321	Multiple osteochondromas	65	4.07	-0.10	67.64	Childhood
RCG080		Niemann-Pick disease	34	4.11	-0.26	62.56	
RFG080	98895	Becker muscular dystrophy	186	4.17	-0.25	69.32	Childhood
RI0080	36204	Intestinal lymphangiectasia	35	4.37	-0.07	83.63	All ages
RDG010	85	Congenital dyserythropoietic aner	36	4.40	-0.06	40.79	Childhood
RNG060		Osteopetrosis	23	4.44	-0.34	49.20	
RN0650	1214	Parry-Romberg syndrome	30	4.44	-0.37	50.63	2-20
RCG070	309015	Familial lipoprotein lipase deficien	12	4.60	-0.17	47.50	Childhood
RF0250	215	Congenital stationary night blindn	10	4.90	0.33	53.24	
RN1360	63	Alport syndrome	479	5.03	-0.31	65.58	Childhood
RFG090		Becker Disease	10	5.05	0.93	39.68	
RDG010	822	Hereditary spherocytosis	653	5.07	-0.48	72.90	All ages
RN0150	1059	Blue rubber bleb nevus	5	5.30	-0.06	76.09	Childhood
RCG050	664	Ornithine transcarbamylase defici	28	5.32	-0.08	82.27	Neonatal, All ages
RNG060		Fairbank disease	5	5.36	-0.01	18.28	
RF0200	891	Familial exudative vitreoretinopat	82	5.51	-0.25	58.33	Infancy, Neonatal
RN0330	98249	Ehlers-Danlos syndrome	766	5.57	-0.48	67.29	Infancy, Neonatal
RFG010	487	Krabbe disease	23	5.79	0.00	50.78	Neonatal, Infancy, Childhood, Adolescent, Adult
RFG110		Vitreoretinal degeneration	21	5.81	-0.07	53.84	
RDG010	84	Fanconi anemia	38	5.90	-0.05	22.20	Childhood
RF0070	36899	Myoclonus-dystonia syndrome	16	5.91	-0.44	53.21	<20
RDG020	330	Factor XII deficiency	4	5.97	-0.04	39.54	All ages
RDG020		Congenital coagulation factors def	864	6.02	-0.41	78.08	
RN0690		Klinefelter syndrome	1445	6.10	-0.48	70.43	
RCG020	418	Congenital adrenal hyperplasia	233	6.49	-0.49	57.14	All ages
RFG070		Centronuclear myopathy	20	6.71	-0.38	46.83	
RFG110	65	Leber congenital amaurosis	70	6.73	-0.08	60.25	Infancy, Neonatal
RFG140		Lattice corneal dystrophy	5	6.85	0.16	42.50	
RD0030	761	Henoch-Schönlein purpura	569	7.23	-0.41	78.99	Childhood
RC0040	169615	Idiopathic central precocious pube	2606	7.37	-0.37	53.78	<8-9
RG0010		Rheumatic endocarditis	560	8.26	-0.17	57.86	

RFG010		Hypomyelinating leukodystrophy t	6	8.53	0.04	31.77	
RN0310	2345	Isolated Klippel-Feil syndrome	39	9.00	-0.37	63.67	Infancy, Neonatal
RCG160	183660	Severe combined immunodeficien	7	9.88	0.17	68.82	Infancy, Neonatal
RFG010	135	CACH (childhood ataxia with centr	12	10.21	-0.07	52.85	Childhood
RCG100	163	Hereditary hyperferritinemia-cata	33	10.36	-0.37	62.70	All ages
RFG090		Thomsen disease	25	10.93	-0.24	51.52	
RCG080	75234	Cholesterol ester storage disease	5	10.98	8.45	27.59	Childhood
RC0080		Total lipodystrophy	23	10.98	0.00	62.33	
RDG010		Favism	578	11.00	-0.39	84.86	
RF0050	101	Dentatorubral pallidoluysian atrop	5	11.44	3.45	67.36	1-60 (average 29)
RC0010	199296	Congenital isolated ACTH deficien	94	11.87	-0.08	73.42	neonatal
RN1290	3463	Wolfram syndrome	22	11.89	-0.08	18.54	<10
RC0150	905	Wilson disease	427	12.42	-0.41	71.77	Childhood
RFG040	95	Friedreich ataxia	267	12.43	-0.23	74.88	Childhood, Adolescent
RF0290	97231	Ligneous conjunctivitis	4	12.44	0.04	63.75	Childhood
RFG090	684	Von Eulenburg disease	10	12.67	1.07	58.00	Adolescent, Adult, Childhood
RCG150		Histiocytosis X	268	13.29	-0.44	71.11	
RN1320	558	Marfan syndrome	838	13.34	-0.46	76.83	All ages
RCG040	470	Lysinuric protein intolerance	4	13.46	1.48	40.57	Infancy, Neonatal
RC0190	91378	Hereditary angioedema	344	13.82	-0.22	86.40	All ages
RN0760	2869	Peutz-Jeghers syndrome	52	14.40	-0.08	72.02	Adolescent, Adult, Childhood
RC0020	478	Kallmann syndrome	356	14.60	-0.42	59.40	Childhood
RFG110	52427	Retinitis punctata albescens	7	14.67	4.00	53.22	Childhood
RFG040	1168	Ataxia with oculomotor apraxia	9	14.96	4.42	18.05	Childhood
RFG060		Hereditary sensory neuropathy	11	15.16	-0.20	54.73	
RCG080	355	Gaucher disease	140	15.39	-0.32	87.22	All ages
RN0620	2796	Pachydermoperiostosis	12	15.79	-0.05	38.54	childhood, adolescence
RG0100	774	Hereditary hemorrhagic telangiect	1102	15.79	-0.42	86.16	Childhood
RDG030		Storage pool deficiency	9	15.89	6.51	54.23	
RFG050	65684	Hirayama disease	5	16.18	15.67	18.67	10-30
RCG070		Homozygous familial hypercholest	27	16.45	0.00	52.13	
RFG080		Limb-girdle muscular dystrophy	33	16.49	0.09	49.18	
RDG030		Secretion deficiency thrombocyto	11	16.62	-0.08	59.75	

RFG110		Usher Syndrome	40	16.96	-0.07	45.98	
RFG110		Retinal hyaline distrophy	5	17.15	-0.25	46.23	
RN0010		Arnold-Chiari malformation	1483	17.38	-0.48	79.54	
RCG040	214	Cystinuria	77	17.69	-0.07	56.33	All ages; median 15y
RCG070	909	Cerebrotendinous xanthomatosis	64	17.98	0.01	64.00	Infancy, Neonatal
RFG080		Erb dystrophy	29	18.02	-0.30	63.75	
RN0580	316	Progressive symmetric erythroker	4	18.53	0.01	48.84	infancy
RCG040		Classic homocystinuria	79	18.66	-0.22	59.60	
RFG070	597	Central core disease	47	19.03	-0.33	63.46	Childhood
RN0630	758	Pseudoxanthoma elasticum	136	19.28	-0.47	75.50	All ages (mostly 10-20)
RCG160	1572	Common variable immunodeficier	155	19.30	-0.07	75.15	All ages
RFG060	166	Charcot-Marie-Tooth disease	559	19.47	-0.05	85.11	
RCG070		Familial hypertriglyceridemia	32	19.63	0.03	52.17	
RC0160	436	Hypophosphatasia	6	19.78	0.96	30.63	All ages
RF0150	2073	Narcolepsy	540	19.79	0.39	81.91	10-30
RN0550	218	Darier disease	294	20.08	-0.47	82.78	All ages, usually around puberty
RFG040	96	Friedreich-like ataxia	43	20.29	-0.43	61.54	5-20
RI0030	2070	Eosinophilic gastroenteritis	149	20.36	-0.10	75.68	30-50
RF0120		Adrenoleukodystrophy	131	20.64	-0.47	77.60	
RN1650	404560	Familial dysplastic nevus syndrom	101	20.74	-0.42	72.86	Childhood, Adolescent, Adult
RCG070	650	Lecithin-cholesterol acyltransferas	5	21.68	9.83	29.97	All ages
RC0060	902	Werner syndrome	14	22.05	0.00	39.52	20-30
RDG020		Antithrombin deficiency	67	22.18	-0.08	77.95	
RF0300	104	Leber hereditary optic neuropathy	212	23.35	-0.04	78.08	18-30
RFG010	58	Alexander disease	8	23.69	-0.03	65.62	All ages
RFG080	269	Landouzy-Dejerine dystrophy	183	25.15	0.00	75.95	3-60
RDG020		Homozygous Factor V Leiden	40	25.27	-0.08	78.86	
RCG040	56	Alkaptonuria	35	25.43	-0.16	74.65	Adult, Infancy
RF0280	156071	Keratoconus	3971	25.69	-0.02	84.46	
RFG090	273	Steinert myotonic dystrophy	354	25.87	-0.45	75.00	Neonatal, Adult
RN0780	892	Von Hippel-Lindau disease	89	26.00	-0.02	73.54	All ages (average 26)
RFG070	98909	Desmin-related myofibrillar myop	11	26.00	9.93	53.39	
RNG060		Fibrous dysplasia	35	26.22	-0.49	62.02	

RN0710	550	MELAS	466	26.27	-0.47	80.44	Adolescent, Adult, Childhood
RFG060	640	Tomaculous neuropathy	121	26.48	-0.03	78.20	10-30
RCG080	324	Fabry disease	270	26.51	-0.22	82.41	Childhood
RF0210	40923	Eales disease	13	26.69	15.25	56.35	20-30
RDG020		Hereditary thrombophilic disorder	1496	26.88	-0.49	85.50	
RI0040		Chronic intestinal pseudoobstruct	138	27.27	-0.31	80.02	
RCG110	79273	Hereditary coproporphyria	5	27.48	17.54	54.58	Adolescent, Adult
RCG070	31150	Tangier disease	7	27.63	0.00	39.65	Neonatal, Infancy, Childhood
RB0050	733	Familial adenomatous polyposis	577	27.63	-0.21	76.92	>10
RFG140		Granular corneal dystrophy	8	27.72	0.00	69.94	
RF0020	480	Kearns-Sayre syndrome	125	27.84	-0.05	80.28	Childhood, Adolescent, Adult
RFG110	1871	Progressive cone dystrophy	76	28.51	-0.05	74.24	
RCG070	31154	Familial hypobetalipoproteinemia	30	29.00	-0.36	69.32	
RB0040	79665	Gardner syndrome	5	29.95	12.95	41.78	
RFG110		Retinitis pigmentosa	1053	29.96	-0.07	85.94	
RFG050	83420	Spinal muscular atrophy type 4	4	30.55	4.25	46.48	10-30
RN0250	1309	Medullary sponge kidney	174	30.84	-0.32	64.57	
RFG110	827	Stargardt disease	270	30.96	-0.06	77.49	3-20
RC0210	117	Behçet disease	1663	31.11	-0.48	77.13	Adult, Adolescent, Childhood
RF0160	2483	Melkersson-Rosenthal syndrome	20	31.30	6.00	70.31	Childhood
RG0110	131	Budd-Chiari syndrome	43	31.65	3.65	60.59	All ages
RFG040		Hereditary spastic paraplegia	424	32.28	-0.37	83.21	
RF0230	263479	Fuchs heterochromic iridocyclitis	174	33.11	7.87	67.16	
RN1570	263440	Neuroacanthocytosis	16	33.52	17.59	51.44	
RDG020		Homozygous G20210A prothromb	14	33.72	-0.07	79.28	
RI0050	171	Primary sclerosing cholangitis	398	33.75	-0.19	84.53	All ages (average 40)
RFG040		Autosomal dominant cerebellar at	27	33.81	-0.39	62.29	
RFG110	1243	Best vitelliform macular dystrophy	101	34.50	0.00	81.77	Childhood, Adolescent
RD0020	447	Paroxysmal nocturnal hemoglobin	116	34.66	11.12	78.68	All ages, pref. adults
RN0720	551	MERRF	76	34.74	0.02	73.60	Childhood, Adult
RF0270	1467	Cogan syndrome	58	34.78	9.29	71.60	<30
RCG030	3143	Schmidt syndrome	56	35.55	0.52	74.93	adult
RN0230	2924	Polycystic liver disease	144	35.77	-0.08	82.40	>40

RC0120	48818	Aceruloplasminemia	7	35.92	3.92	53.07	adults
RFG040		Autosomal dominant spinocerebe	209	36.12	0.56	69.56	
RDG020		Combined Factor V Leiden and het	75	36.19	-0.07	77.40	
RGG010		Thrombocytopenic thrombotic pu	8	36.88	21.12	62.03	
RN1720	3437	Vogt-Koyanagi-Harada disease	78	37.18	9.49	61.71	10-52 (average 30)
RCG110	79276	Acute intermittent porphyria	19	37.77	2.97	57.57	Adolescent, Adult
RC0200	60	Congenital alpha-1-antitrypsin def	350	37.96	-0.08	75.39	All ages
RL0020	1656	Dermatitis herpetiformis	302	38.07	0.42	84.95	All ages
RCG150		Non-Langerhans cell histiocytosis	10	38.24	-0.07	69.42	
RA0010	548	Hansen disease (Leprosy)	13	38.27	18.05	55.79	All ages
RB0060	538	Lymphangioleiomyomatosis	160	38.42	0.02	72.02	Adults
RFG040		Periodic ataxia	12	38.89	0.00	78.74	
RGG010	54057	Thrombotic thrombocytopenic pu	207	38.93	9.35	77.18	All ages
RFG010	2478	Megalencephalic leukoencephalop	8	39.10	0.33	55.64	Infancy, Neonatal
RA0030	91546	Lyme disease	106	39.52	0.66	79.26	All ages
RC0090	36397	Dercum disease	25	39.79	0.02	66.10	45-60
RN1490		Isaacs syndrome	23	40.65	17.09	74.91	
RN0220	53035	Caroli disease	52	40.70	-0.26	72.79	All ages
RCG110	79473	Porphyria variegata	8	40.89	21.83	47.45	Adolescent, Adult
RCG070	425	Familial hypoalphalipoproteinemia	4	41.05	35.42	51.67	All ages
RG0090	3287	Takayasu arteritis	284	41.17	9.38	74.03	10-30
RM0030	809	Mixed connective tissue disease	774	41.67	-0.29	87.90	15-35
RCG100	209981	Iron refractory iron deficiency ane	4	41.74	30.83	51.28	Infancy, Neonatal
RFG140	98625	Superficial corneal dystrophy	7	42.63	32.95	72.26	3-20
RFG140	98973	Posterior polymorphous corneal d	4	43.64	25.03	65.17	Childhood
RJ0030	37202	Interstitial cystitis	930	43.97	0.01	84.01	All ages
RFG090	606	Proximal myotonic myopathy	26	44.76	0.52	65.36	Adult
RFG040	1173	Cerebellar ataxia-hypogonadism	14	44.82	0.00	75.09	Adolescent, Adult, Childhood
RCG100		Hereditary hemochromatosis	992	44.93	-0.45	87.72	
RG0030	767	Polyarteritis nodosa	161	45.36	0.50	80.51	40-60
RFG080	1876	Oculogastrointestinal muscular dy	13	45.52	-0.38	66.55	Childhood
RFG140	98627	Posterior corneal dystrophy	15	46.08	-0.01	60.37	All ages
RF0090		Idiopathic torsion dystonia	555	46.42	0.00	80.05	

RFG050	481	Kennedy disease	31	47.16	19.45	77.30	30-60
RI0010	930	Idiopathic achalasia	1060	47.17	-0.08	89.49	30-60
RF0080	399	Huntington disease	1188	47.70	-0.07	84.91	All ages (average 30-50)
RFG040		Idiopathic spinocerebellar ataxia	44	48.83	5.14	72.55	
RG0060	375	Goodpasture syndrome	24	48.92	17.19	76.07	Adolescent, Adult
RM0040	3165	Eosinophilic fasciitis	73	49.24	3.49	79.21	Adult
RM0010	221	Dermatomyositis	825	49.57	-0.09	85.79	All ages
RM0060	728	Relapsing polychondritis	83	50.66	5.61	80.06	40-55
RG0050	183	Churg-Strauss syndrome	748	50.77	0.00	85.26	15-70
RCG010		Conn syndrome	18	51.14	25.91	72.35	
RCG110	101330	Porphyria cutanea tarda	53	51.47	22.11	71.52	Adult
RA0020	3452	Whipple disease	54	51.87	7.50	80.11	51 (Mean); range: 4-77 [26]
RL0060		Lichen sclerosus	1534	52.25	0.00	89.10	
RN1610	2905	POEMS syndrome	31	52.27	34.02	77.80	Adult, Elderly
RNG060		Congenital osteodystrophy	45	52.48	-0.04	76.79	
RFG140	98974	Fuchs endothelial dystrophy	13	52.57	43.03	70.00	40-60
RG0070	900	Wegener granulomatosis	712	53.07	0.00	88.37	All ages (average 45)
RL0030		Pemphigus vulgaris	1736	53.20	4.51	89.94	
RM0050		Diffuse fasciitis	9	53.27	4.02	85.34	
RN1700	816	Sjögren-Larsson syndrome	53	53.37	-0.04	76.83	Infancy, Neonatal
RF0180	2932	Chronic inflammatory demyelinati	1414	54.11	1.64	87.10	40-60
RM0020	732	Polymyositis	708	54.75	-0.43	89.00	45-60
RFG140	98958	Droplet cornea	4	54.94	42.10	75.26	
RF0190	43393	Lambert-Eaton syndrome	50	55.31	-0.03	75.57	>40
RFG040	102	Multisystem atrophy	48	55.36	39.62	71.81	>30; average 55-60
RFG140		Cogan dystrophy	8	55.45	0.00	83.03	
RJ0020	49041	Retroperitoneal fibrosis	299	57.46	5.38	85.59	Adult
RF0110	35689	Primary lateral sclerosis	225	57.85	0.52	82.02	Elderly, Adult
RFG040	559	Marinesco-Sjögren syndrome	27	58.17	0.33	85.13	Infancy, Childhood
RC0110		Mixed cryoglobulinemia	1227	58.95	0.00	88.42	
RG0020	727	Microscopic polyangiitis	438	61.66	1.45	89.50	>10; average 50-60
RL0050	46486	Mucous membrane pemphigoid	252	62.17	7.32	85.78	60-70
RF0100	803	Amyotrophic lateral sclerosis	7047	65.12	-0.16	89.31	about 60

RF0170	240071	Steele-Richardson-Olszewski disea	306	68.24	43.49	85.80	50-70
RG0080	397	Giant cell arteritis	1424	71.57	10.38	89.59	>50
RL0040		Bullous pemphigoid	1816	74.80	-0.36	89.92	

References

[24] ORPHANET. Disease data sheets. Available online: http://www.orpha.net/consor/cgi-bin/Disease_Search_Simple.php?lng=EN (Accessed on 3 April 2017).

[26] Dutly F., Altwegg M. Whipple's Disease and "Tropheryma whippelii". Clin Microbiol Rev 2001, 14, 561–583.
