# Supplementary material for: The Occurrence of 275 Rare Diseases and 47 Rare Disease Groups in Italy. Results from the National Registry of Rare Diseases
Source: Int J Environ Res Public Health. 2018 Jul 12;15(7):1470. doi: 10.3390/ijerph15071470 (PMC6068991; doi:10.3390/ijerph15071470)
Supplement: Supplementary file 1 [file ijerph-15-01470-s001.zip › Table S6.pdf]

Article submitted to International Journal of Environmental Research and Public Health
The Occurrence of 275 Rare Diseases and 47 Rare Disease Groups in Italy. Results from the National Registry of Rare Diseases.
Domenica Taruscio*, Luciano Vittozzi, Adele Rocchetti, Paola Torreri, Luca Ferrari

Table S6. Sex distribution of the rare diseases reported to RNMR

Record source: Subset 1; **Notifying regions**: all; **Record selection**: records with diagnosis indicating a specific disease (i.e.: excluding disease groups) and at least 4 records per disease; **Total records**:119762.
Note: The number of records is indicated with the only aim of allowing a better assessment of the statistical data presented and cannot be used as an indication of the disease or exemption code frequency. Where the percentages in males and females sum to less than 100, the difference represents records with missing sex data.

RNMR Code	ORPHA Code	Disease	Records (N)	Females (%)	Males (%)	F:M ratio and other literature information reported by ORPHANET data sheets [24]	Comment
RNG040	1791	Frontofacionasal dysplasia	7	100	0		
RCG100	209981	Iron refractory iron deficiency anemia	4	100	0		
RN0680	881	Turner syndrome	1754	99	1	Almost exclusively in F	
RF0040	778	Rett syndrome	603	97	3	Predominant in F	
RB0060	538	Lymphangioleiomyomatosis	180	97	3	Almost exclusively in F	
RN0510	464	Incontinentia pigmenti	95	97	3	20:1	
RJ0030	37202	Interstitial cystitis	1192	95	5	9:1	
RDG030		Storage pool deficiency	11	91	9		
RN1700	816	Sjögren-Larsson syndrome	93	90	9		
RGG010		Thrombocytopenic thrombotic purpura	10	90	10		
RFG040	559	Marinesco-Sjögren syndrome	30	90	10		
RC0040	169615	Idiopathic central precocious puberty	3686	89	11	10:1	
RFG140	98973	Posterior polymorphous corneal dystrop	8	88	13		
RCG110	79473	Porphyria variegata	8	88	13	Predominant in F	
RN1130	1297	Branchio-oculo-facial syndrome	8	88	13		
RCG030	3143	Schmidt syndrome	72	86	14		
RNG040	1452	Cleidocranial dysostosis	7	86	14		
RNG050	429	Hypochondroplasia	7	86	14		
RM0030	809	Mixed connective tissue disease	1092	86	14	10:1	
RG0090	3287	Takayasu arteritis	346	85	14	Predominant in F	
RF0160	2483	Melkersson-Rosenthal syndrome	25	84	16		
RN0590	317	Erythrokeratodermia variabilis	6	83	17		
RFG140	98958	Droplet cornea	17	82	18		
RC0160	436	Hypophosphatasia	11	82	18		
RDG030		Secretion deficiency thrombocytopathy	11	82	18		
RN0830	125	Bloom syndrome	5	80	20		
RCG110	79273	Hereditary coproporphyria	5	80	20	Predominant in F	
RFG140	98974	Fuchs endothelial dystrophy	30	80	20	3-4:1	
RCG140	581	Mucopolysaccharidosis type 3	5	80	20		
RN0900	2059	Fryns syndrome	5	80	20		
RFG040		Sub-acute cerebellar degeneration	14	79	21		
RC0080		Total lipodystrophy	35	77	23		
RGG010	54057	Thrombotic thrombocytopenic purpura	223	76	24		
RN0610	2092	Focal dermal hypoplasia	21	76	24	X-linked dominant	
RN0230	2924	Polycystic liver disease	171	76	24	Predominant in F	
RCG070	14	Abetalipoproteinemia	12	75	25		
RF0240	98981	Essential iris atrophy	4	75	25		
RCG060	348	Fructose-1,6-bisphosphatase deficiency	4	75	25		
RCG070		Homozygous familial hypercholesterolei	8	75	25		
RC0050	508	Leprechaunism	4	75	25		
RCG040	35	Propionic acidemia	4	75	25		
RN0370	239	Dyggve-Melchior-Clausen disease	4	75	25		
RN1680	3352	Tricho-dento-osseous syndrome	4	75	25		
RN0480	3377	Trismus-pseudocamptodactyly	4	75	25		
RN0630	758	Pseudoxanthoma elasticum	197	75	25	2:1	
RNG060	240	Dyschondrosteosis	43	74	26		
RCG020	418	Congenital adrenal hyperplasia	374	74	26		
RG0080	397	Giant cell arteritis	1733	74	26	2:1	
RI0040		Chronic intestinal pseudoobstruction	148	74	26		
RN0330	98249	Ehlers-Danlos syndrome	1040	73	27		

RNG060	562	McCune-Albright syndrome	29	72	28		
RN1720	3437	Vogt-Koyanagi-Harada disease	90	72	28	Predominant in F	
RCG160	183660	Severe combined immunodeficiency	7	71	29	One form X-linked	
RCG050		Hereditary hyperammonemia	7	71	29		
RFG110	52427	Retinitis punctata albescens	7	71	29		
RN1370	64	Alström syndrome	14	71	29		
RN1670		Multiple pterygium syndrome	7	71	29		
RFG040		Autosomal dominant cerebellar ataxia	31	71	29		
RC0110		Mixed cryoglobulinemia	1609	71	29		
RG0110	131	Budd-Chiari syndrome	64	70	30		
RN1290	3463	Wolfram syndrome	33	70	30		
RM0020	732	Polymyositis	974	69	30	2:1	
RN0340	974	Adams-Oliver syndrome	13	69	31		
RC0030		Reifenstein syndrome	45	69	31		
RM0010	221	Dermatomyositis	1084	69	31	2:1	
RC0090	36397	Dercum disease	31	68	32	Predominant in F	
RDG020		Hereditary thrombophilic disorders	3677	67	33		
RCG050	23	Argininosuccinatelyase deficiency	12	67	33		
RN0150	1059	Blue rubber bleb nevus	9	67	33		
RF0290	97231	Ligneous conjunctivitis	6	67	33		
RN0500		Cutis laxa	12	67	33		
RN1440	2710	Oculodentodigital dysplasia	6	67	33		
RFG140	98627	Posterior corneal dystrophy	15	67	33		
RFG110		Retinal hyaline distrophy	6	67	33		
RCG040	407	Non-ketotic hyperglycinemia	6	67	33		
RFG010		Hypomyelinating leukodystrophy type 2	6	67	33		
RFG090	684	Von Eulenburg disease	12	67	33		
RCG140	579	Mucopolysaccharidosis type 1	18	67	33		
RCG140	582	Mucopolysaccharidosis type 4	9	67	33		
RNG060		Congenital osteodystrophy	45	67	33		
RN0960	163634	Maffucci syndrome	24	67	33		
RL0050	46486	Mucous membrane pemphigoid	308	66	34	Predominant in F	
RN0250	1309	Medullary sponge kidney	184	66	34		
RF0090		Idiopathic torsion dystonia	948	65	35		
RDG020		Homozygous Factor V Leiden	40	65	35		
RNG070		Autosomal recessive congenital ichthyos	17	65	35		
RCG100	163	Hereditary hyperferritinemia-cataract sy	34	65	35		
RCG050	664	Ornithine transcarbamylase deficiency	28	64	36	The severe, neonatal-onset form almost exclusively in M	(1)
RDG020		Homozygous G20210A prothrombin	14	64	36		
RDG020		Antithrombin deficiency	67	64	36		
RFG070	597	Central core disease	63	63	37		
RDG020		Congenital coagulation factors deficienc	1151	63	37		
RCG110	79276	Acute intermittent porphyria	19	63	37		
RDG020		Dysfibrinogenemia	27	63	37		
RN1480	435	Ito hypomelanosis	51	63	37		
RM0060	728	Relapsing polychondritis	115	63	37		
RN0010		Arnold-Chiari malformation	1830	63	37		
RC0120	48818	Aceruloplasminemia	8	63	38		
RFG070		Nemaline myopathy	16	63	38		
RN0300	3027	Caudal regression sequence	40	63	38		
RN0360	1465	Coffin-Siris syndrome	16	63	38		
RFG040	96	Friedreich-like ataxia	45	62	38		
RNG040	861	Treacher-Collins syndrome	29	62	38		
RN1150	1340	Cardiofaciocutaneous syndrome	65	62	38		
RN0310	2345	Isolated Klippel-Feil syndrome	70	61	39		
RN0060	2162	Holoprosencephaly	62	61	39		
RA0030	91546	Lyme disease	170	61	38		
RD0040	2686	Cyclic neutropenia	82	61	39		
RNG070	313	Lamellar ichthyosis	64	61	39		
RN0650	1214	Parry-Romberg syndrome	64	61	39	Slightly predominant in F	
RFG080		Limb-girdle muscular dystrophy	33	61	39		
RFG040	102	Multisystem atrophy	48	60	40		
RN0700	280	Wolf-Hirschhorn syndrome	88	60	40	2:1	
RFG140		Lattice corneal dystrophy	10	60	40		

RN0580	316	Progressive symmetric erythrokeratoder	5	60	40		
RFG060	99951	Charcot-Marie-Tooth disease type 4E	5	60	40		
RN0350	192	Coffin-Lowry syndrome	10	60	40	X-linked dominant	
RN1600	699	Pearson syndrome	5	60	40		
RDG020	903	Von Willebrand disease	1047	60	40		
RC0170		Vitamin D-resistent hypophosphatemic r	317	60	40		
RCG060	469	Hereditary fructose intolerance	70	59	41		
RNG060		Fibrous dysplasia	68	59	41		
RDG020		Combined Factor V Leiden and heterozy	75	59	41		
RNG040		Pierre Robin syndrome	53	58	42		
RN1180	324764	Trichorhinophalangeal syndrome	53	58	42		
RN1510	90308	Klippel-Trénaunay syndrome	226	58	42		
RN0080	1764	Familial dysautonomia	12	58	42		
RFG140		Granular corneal dystrophy	12	58	42		
RCG140	581	Mucopolysaccharidosis type 3	12	58	42		
RNG060		Osteopetrosis	36	58	42		
RN0550	218	Darier disease	361	58	42		
RM0040	3165	Eosinophilic fasciitis	98	58	42		
RL0030		Pemphigus vulgaris	2070	58	42		
RN1660	35125	Epidermal nevus syndrome	26	58	42		
RN1410	199	Cornelia de Lange syndrome	111	58	42	One form X-linked recessive	
RN0130	35737	Morning glory syndrome	14	57	43		
RFG040	98	Spastic ataxia of Charlevoix-Saguenay	14	57	43		
RN0240	2138	True hermaphroditism	21	57	43		
RFG070		Congenital myopathy with fiber type dis	7	57	43		
RN0710	550	MELAS	618	57	43		
RN1190	2614	Nail-patella syndrome	51	57	43		
RD0010		Hemolytic-uremic syndrome	368	57	43		
RF0020	480	Kearns-Sayre syndrome	215	57	43		
RN1620	783	Rubinstein-Taybi syndrome	90	57	43		
RDG020	745	Protein C deficiency	233	57	43		
RCG080	324	Fabry disease	410	57	43	X-linked recessive	
RN0640	1114	Aplasia cutis congenita	16	56	44		
RG0050	183	Churg-Strauss syndrome	936	56	44		
RNG040		Oculomaxillofacial dysostosis	68	56	44		
RN0110	77	Aniridia	104	56	44		
RD0020	447	Paroxysmal nocturnal hemoglobinuria	155	55	45		
RF0110	35689	Primary lateral sclerosis	258	55	45		
RL0060		Lichen sclerosus	1971	55	45		
RN1450	94068	Congenital spondyloepiphyseal dysplasia	29	55	45		
RF0060	98261	Progressive myoclonic epilepsy	174	55	45		
RDG020	327	Factor VII deficiency	98	55	45		
RN1570	263440	Neuroacanthocytosis	20	55	45		
RB0010	654	Wilms tumor	491	55	44		
RN0020	199642	Microcephaly	421	55	45		
RF0270	1467	Cogan syndrome	73	55	45		
RN0770	3205	Sturge-Weber syndrome	170	55	45		
RN0290		Camptodactyly	11	55	45		
RP0010	290	Congenital rubella syndrome	33	55	45		
RI0080	36204	Intestinal lymphangiectasia	44	55	45		
RB0040	79665	Gardner syndrome	11	55	45		
RN0740		Ivemark syndrome	11	55	45		
RN1050		Rieger syndrome	11	55	45		
RN0780	892	Von Hippel-Lindau disease	191	54	46		
RNG050	321	Multiple osteochondromas	96	54	46	1:1.5	(2)
RDG020	326	Factor V deficiency	24	54	46		
RL0040		Bullous pemphigoid	2296	54	46		
RN0750	805	Tuberous sclerosis	1145	54	46		
RDG010		Thalassemias	2534	54	46		
RFG040	95	Friedreich ataxia	284	54	46		
RCG040	511	Maple syrup urine disease	26	54	46		
RNG040	207	Crouzon disease	52	54	46		
RC0210	117	Behçet disease	2307	53	46		
RG0100	774	Hereditary hemorrhagic telangiectasia	1231	53	47		
RCG080		Niemann-Pick disease	60	53	47		

RC0060	902	Werner syndrome	15	53	47		
RNG060	666	Osteogenesis imperfecta	479	53	47		
RN1530	500	LEOPARD syndrome	47	53	47		
RN0950	98861	Primary ciliary dyskinesia, Kartagener ty	463	53	47		
RN0180	1203	Duodenal atresia	83	53	47		
RN0120		Congenital colobomatous optic disc	187	53	47		
RN1200		Smith-Lemli-Opitz syndrome type 1	34	53	47		
RN0820		Beckwith-Wiedemann syndrome	333	53	47		
RDG010	85	Congenital dyserythropoietic anemia	36	53	47	One form X-linked recessive	
RN0540	1556	Cutis marmorata telangiectatica congen	36	53	47		
RN0600	312	Epidermolytic ichthyosis	36	53	47		
RG0070	900	Wegener granulomatosis	938	53	47		
RN0850	138	CHARGE syndrome	165	53	47		
RDG010		Pyruvate kinase deficiency	19	53	47		
RN0050		Lissencephaly	166	52	48		
RCG080	355	Gaucher disease	208	52	48		
RDG010	84	Fanconi anemia	42	52	48	One form X-linked recessive	
RFG010	512	Metachromatic leukodystrophy	23	52	48		
RN0990	570	Moebius syndrome	73	52	48		
RC0190	91378	Hereditary angioedema	515	52	48		
RCG040		Classic homocystinuria	106	52	48		
RD0030	761	Henoch-Schönlein purpura	644	52	48	1:1.5	
RCG160	1572	Common variable immunodeficiency	155	52	48		
RF0080	399	Huntington disease	1553	51	49		
RN1360	63	Alport syndrome	525	51	49	Severe, quickly progressing form predominant in M	
RFG040		Autosomal dominant spinocerebellar ata	209	51	49		
RN0210		Biliary atresia	336	51	49		
RG0020	727	Microscopic polyangiitis	495	51	49		
RN0720	551	MERRF	92	51	49		
RF0170	240071	Steele-Richardson-Olszewski disease	371	51	49		
RN0220	53035	Caroli disease	61	51	49	Slightly predominant in F	
RFG110	1871	Progressive cone dystrophy	124	51	49		
RN1220	828	Stickler syndrome	85	51	49		
RG0030	767	Polyarteritis nodosa	202	50	50		
RFG110	827	Stargardt disease	492	50	50		
RFG040		Periodic ataxia	16	50	44		
RFG040	100	Ataxia-telangiectasia	48	50	50		
RNG040	1308	C syndrome	6	50	50		
RFG010	135	CACH (childhood ataxia with central ner	12	50	50		
RCG050	187	Citrullinemia	10	50	50		
RN0270	3181	Sprengel deformity	6	50	50		
RFG130	137672	Pellucid marginal degeneration	6	50	50		
RFG040	1173	Cerebellar ataxia-hypogonadism	18	50	50		
RCG060		Congenital disorder of glycosylation	6	50	50		
RFG080		Erb dystrophy	46	50	50		
RFG090	606	Proximal myotonic myopathy	26	50	50		
RF0250	215	Congenital stationary night blindness	16	50	50		
RP0070		Congenital hepatic fibrosis	52	50	50		
RN0260	294975	Phocomelia	46	50	50		
RCG040	470	Lysinuric protein intolerance	4	50	50		
RCG150		Non-Langerhans cell histiocytosis	10	50	50		
RFG010	2478	Megalencephalic leukoencephalopathy	8	50	50		
RN0670	281	Cri du chat syndrome	60	50	50		
RF0010	726	Alpers syndrome	16	50	50		
RFG060	773	Refsum disease	4	50	50		
RFG090		Thomsen disease	68	50	50		
RFG050	83330	Werdnig-Hoffman disease	20	50	50	Slightly predominant in M	(3)
RCG040	26	Methylmalonic acidemia with homocyst	16	50	50		
RCG140	582	Mucopolysaccharidosis type 4	20	50	50		
RFG060		Hereditary sensory neuropathy	12	50	50		
RN0140		Pupillar membrane persistence	6	50	50		
RDG030	274	Bernard-Soulier syndrome	6	50	50		
RN0970	560	Marshall syndrome	4	50	50		
RN1710	453	Tay syndrome	6	50	50		

RN1750	3449	Weill-Marchesani syndrome	4	50	50		
RB0020	790	Retinoblastoma	675	50	50		
RFG060	166	Charcot-Marie-Tooth disease	706	50	50		
RN0170	1201	Atresia of small intestine	93	49	49		
RI0010	930	Idiopathic achalasia	1941	49	50		
RN1270	904	Williams syndrome	490	49	51		
RDG010	232	Sickle cell anemia	573	49	50		
RNG050	15?	Achondroplasia	124	49	51		
RC0200	60	Congenital alpha-1-antitrypsin deficienc	393	49	51		
RFG110		Retinitis pigmentosa	1633	49	51		
RN0910	374	Goldenhar syndrome	260	49	51		
RCG040		Cystinosis	43	49	51		
RCG150		Histiocytosis X	291	49	51		
RCG070	909	Cerebrotendinous xanthomatosis	68	49	51		
RDG010	124	Blackfan-Diamond anemia	66	48	52		
RCG160	567	Di George syndrome	427	48	52		
RN0040	475	Joubert syndrome	120	48	52		
RN0570		Epidermolysis bullosa	358	48	52		
RCG040	238583	Hyperphenylalaninemia	673	48	52		
RF0230	263479	Fuchs heterochromic iridocyclitis	267	48	52		
RCG070		Homozygous familial hypercholesteroler	48	48	52		
RN1310	739	Prader-Willi syndrome	591	48	52		
RCG070		Disorders of fatty acid oxidation and ket	117	48	52		
RL0020	1656	Dermatitis herpetiformis	379	48	52		
RN1350		Alagille syndrome	101	48	52		
RFG110		Usher Syndrome	40	48	53		
RCG070		Beta-oxydation deficiency	19	47	53		
RNG030	87	Apert syndrome	19	47	53		
RCG060		Glycogen storage disease	376	47	52		
RDG010	822	Hereditary spherocytosis	712	47	53		
RFG110	1243	Best vitelliform macular dystrophy	146	47	53	1:3	(4)
RN0090		Axenfeld-Rieger anomaly	34	47	53		
RNG070	634	Netherton syndrome	17	47	53		
RN1760	912	Zellweger syndrome	17	47	53		
RN1210	819	Smith-Magenis syndrome	66	47	53		
RN0760	2869	Peutz-Jeghers syndrome	81	47	53		
RB0050	733	Familial adenomatous polyposis	828	47	53		
RCG010		Conn syndrome	30	47	53		
RCG110		Erythropoietic protoporphyria	30	47	53		
RN1240	857	Townes-Brocks syndrome	15	47	53		
RN0520	910	Xeroderma pigmentosum	30	47	53		
RN1300	72	Angelman syndrome	271	46	54		
RN1490		Isaacs syndrome	28	46	54		
RF0150	2073	Narcolepsy	610	46	54		
RN0560	1775	Dyskeratosis congenita	13	46	54		
RF0030	506	Leigh syndrome	185	46	54		
RN0320	2368	Gastroschisis	72	46	53		
RN1320	558	Marfan syndrome	1524	46	54		
RFG140		Cogan dystrophy	11	45	55		
RCG040	293355	Methylmalonic acidemia	11	45	55		
RN1730	893	WAGR syndrome	11	45	55		
RN1080	813	Silver-Russell syndrome	130	45	55		
RF0140	3451	West syndrome	627	45	55	Predominant in M	
RCG040		Oculocutaneous albinism	148	45	55		
RFG090	273	Steinert myotonic dystrophy	526	45	55		
RC0180	205	Crigler-Najjar syndrome	51	45	55		
RFG010	51	Aicardi-Goutieres syndrome	20	45	55		
RN0860	3157	De Morsier syndrome	76	45	54		
RFG080	269	Landouzy-Dejerine dystrophy	293	45	55		
RN0660	870	Down syndrome	3213	45	55		
RCG060	352	Galactosemia	65	45	55		
RF0100	803	Amyotrophic lateral sclerosis	8184	45	55	1:1.5	
RCG160	331223	Iper-Ige syndrome	9	44	56		
RFG040	1168	Ataxia with oculomotor apraxia	9	44	56		
RNG060	289	Ellis-Van Creveld syndrome	9	44	56		

RNG040	2108	Hallermann-Streiff syndrome	9	44	56		
RN0470	669	Otopalatodigital syndrome	9	44	56	X-linked dominant	
RFG040		Hereditary spastic paraplegia	475	44	56		
RFG110	65	Leber congenital amaurosis	122	44	56		
RCG040	214	Cystinuria	77	44	56		
RN0100	708	Peters anomaly	25	44	56		
RN0890	2053	Freeman-Sheldon syndrome	16	44	56		
RN0930	392	Holt-Oram syndrome	32	44	56		
RCG040		Organic acidemias and primary lactic aci	197	44	56		
RC0150	905	Wilson disease	607	43	57		
RFG060	640	Tomaculous neuropathy	144	43	57	Autosomal dominant	(5)
RDG020	98880	Afibrinogenemia	7	43	57		
RFG140	98625	Superficial corneal dystrophy	7	43	57	One form X-linked recessive	
RFG010	141	Canavan disease	7	43	57		
RN1740	899	Walker-Warburg syndrome	7	43	57		
RP0040	1915	Fetal alcohol syndrome	87	43	57		
RF0130	2382	Lennox-Gastaut syndrome	422	42	58		
RG0010		Rheumatic endocarditis	600	42	58		
RCG040	56	Alkaptonuria	38	42	58		
RN0390	380	Greig cephalopolysyndactyly syndrome	19	42	58		
RN1010	648	Noonan syndrome	714	42	58		
RN1140	107	Branchio-oto-renal syndrome	43	42	58		
RN0880	1896	EEC syndrome	164	41	59		
RC0010	199296	Congenital isolated ACTH deficiency	138	41	59		
RFG050	83419	Kugelberg-Welander disease	34	41	59		
RF0190	43393	Lambert-Eaton syndrome	56	41	59		
RN0430	2911	Poland syndrome	475	41	59	Predominant in M	
RN0190	96346	Anorectal malformation	519	41	59		
RFG040		Idiopathic spinocerebellar ataxia	44	41	59		
RFG070		Centronuclear myopathy	27	41	56		
RFG080	1876	Oculogastrointestinal muscular dystroph	52	40	60		
RN1250	887	VACTERL/VATER association	102	40	60		
RN1650	404560	Familial dysplastic nevus syndrome	219	40	60		
RF0050	101	Dentatorubral pallidoluysian atrophy	5	40	60		
RCG070		Carnitine palmitoyltransferase deficienc	10	40	60		
RN0530		Keratosis follicularis spinulosa decalvans	10	40	60		
RC0070	309845	Disorder of zinc metabolism	5	40	60		
RCG080	75234	Cholesterol ester storage disease	5	40	60		
RM0050		Diffuse fasciitis	10	40	60		
RDG020	331	Factor XIII deficiency	5	40	60		
RCG070	650	Lecithin-cholesterol acyltransferase defi	5	40	60		
RI0070	2290	Microvillus inclusion disease (MVID)	10	40	60	1:1.5	
RF0210	40923	Eales disease	20	40	60	Predominant in M	
RCG140	583	Mucopolysaccharidosis type 6	5	40	60		
RN0160		Esophageal atresia and/or Isolated trach	326	40	60		
RI0050	171	Primary sclerosing cholangitis	502	40	60	1:2	
RN0030		Cerebellar agenesis	28	39	61		
RCG010	112	Bartter syndrome	74	39	61		
RI0030	2070	Eosinophilic gastroenteritis	187	39	61		
RN0940	2322	Kabuki make-up syndrome	152	39	61		
RCG070	31150	Tangier disease	13	38	62		
RN1380	110	Bardet-Biedl syndrome	65	38	62		
RFG050	83418	Spinal muscular atrophy type 2	13	38	62		
RG0060	375	Goodpasture syndrome	29	38	62		
RF0070	36899	Myoclonus-dystonia syndrome	16	38	63		
RN0870	235	Dubowitz syndrome	16	38	63		
RF0300	104	Leber hereditary optic neuropathy	427	37	63		
RG0040	2331	Kawasaki disease	1088	37	63		
RCG070	31154	Familial hypobetalipoproteinemia	48	37	63		
RP0050	70590	Infantile apnea	166	37	63		
RFG010	58	Alexander disease	11	36	64		
RF0280	156071	Keratoconus	11022	36	64		
RN0280	950	Acrodysostosis	14	36	64		
RCG050	247525	Argininosuccinate synthase deficiency	14	36	64		
RFG010	702	Pelizaeus-Merzbacher disease	17	35	65	Predominant in M	

RN0410	2311	Jarcho-Levin syndrome	17	35	65		
RFG070	98909	Desmin-related myofibrillar myopathy	11	35	65		
RN1590	884	Pallister-Killian syndrome	26	35	65		
RN1040	710	Pfeiffer syndrome	32	34	66		
RNG060		Fairbank disease	6	33	67		
RDG020	328	Factor X deficiency	9	33	67	Autosomal recessive	
RFG010	487	Krabbe disease	24	33	67		
RCG040		Tyrosinemia	12	33	67		
RJ0020	49041	Retroperitoneal fibrosis	343	32	67		
RF0180	2932	Chronic inflammatory demyelinating pol	1749	32	68		
RFG110		Vitreoretinal degeneration	30	30	70		
RP0060	415286	Bilirubin encephalopathy	10	30	70		
RFG090		Becker Disease	10	30	70		
RN1520	98818	Landau-Kleffner syndrome	27	30	70	1:2	
RN1100	808	Seckel syndrome	17	29	71		
RDG010		Favism	768	29	71		
RCG070	309015	Familial lipoprotein lipase deficiency	14	29	71		
RNG060	1522	Craniometaphyseal dysplasia	14	29	71		
RFG080	97242	Congenital muscular dystrophy	14	29	71		
RA0010	548	Hansen disease (Leprosy)	21	29	71		
RCG060	61	Mannosidosis	7	29	71		
RN1430	220	Denys-Drash syndrome	7	29	71		
RN1170	744	Proteus syndrome	21	29	71		
RF0200	891	Familial exudative vitreoretinopathy	127	28	71	One form predominant in M	
RNG040		Primary craniosynostosis	447	27	73		
RN0400	1540	Jackson-Weiss syndrome	15	27	73		
RN0200	388	Hirschsprung disease	323	26	74		
RA0020	3452	Whipple disease	62	26	74	--	(6)
RCG040		Glutaric aciduria	4	25	75		
RCG050	90	Arginase deficiency	4	25	75		
RFG040		Congenital ataxia	4	25	75		
RN0800	83	Antley-Bixler syndrome	4	25	75		
RN1610	2905	POEMS syndrome	32	25	75	1:2.5	
RFG050	83420	Spinal muscular atrophy type 4	4	25	75		
RCG100		Hereditary hemochromatosis	1418	24	76		
RN1330	908	Fragile X syndrome	479	24	76	X-linked dominant	(7)
RDG030	849	Glanzmann thrombasthenia	13	23	77		
RCG070		Familial hypertriglyceridemia	35	23	77		
RF0120		Adrenoleukodystrophy	189	23	77		
RN1400	191	Cockayne syndrome	9	22	78		
RCG160	183669	Agammaglobulinemia	111	22	78		
RFG050	65684	Hirayama disease	5	20	80	1:20	
RN0490	3447	Weaver syndrome	10	20	80		
RFG080	98895	Becker muscular dystrophy	221	19	81	Predominant in M	
RD0050	379	Chronic granulomatous disease	87	18	82		
RQ0010	221117	Gerstmann syndrome	6	17	83		
RN1070	97360	Robinow syndrome	6	17	83		
RC0020	478	Kallmann syndrome	509	16	84	1:5	
RCG110	101330	Porphyria cutanea tarda	53	15	85	Predominant in M	
RN1020		Opitz syndrome	20	15	85		
RN1640	1466	COFS syndrome	7	14	86		
RN1120		Simpson-Golabi-Behmel syndrome	7	14	86		
RN0620	2796	Pachydermoperiostosis	15	13	87	1:7	
RFG050	481	Kennedy disease	51	12	88	X-linked recessive	
RDG020	98879	Hemophilia B	271	11	89	Predominant in M	
RJ0010	223	Nephrogenic diabetes insipidus	66	11	89		
RN0790	915	Aarskog-Scott syndrome	38	8	92	Predominant in M	
RDG020	98878	Hemophilia A	1577	7	93	Predominant in M	
RFG080	98896	Duchenne muscular dystrophy	258	5	95	Predominant in M	
RNG070	461	Recessive X-linked ichthyosis	65	3	97	Almost exclusively in M	
RN0690		Klinefelter syndrome	2071	0	100		
RDG020	330	Factor XII deficiency	4	0	100		
RCG070	425	Familial hypoalphalipoproteinemia	4	0	100		
RCG120	510	Lesch-Nyhan disease	12	0	100	Severe form predominant in M	

RCG040	534	Lowe syndrome	5	0	100	Almost exclusively in M
RN1580	649	Norrie disease	5	0	100	Almost exclusively in M
RCG140	580	Mucopolysaccharidosis type 2	27	0	100	Almost exclusively in M
RN1000	245	Nager syndrome	4	0	100	

Comments

(1) The cases reported to RNMR show an age at onset spanning from birth to 82 years

(2) In a cohort of 529 patients, a mild phenotype was more frequent in females, while a severe phenotype was more frequent in males [27]. The difference between our and literature data reported by Orphanet may depend on population selection biases.

(3) Higher prevalence in males is observed after onset age 37 months [28]. Our 13 cases show an onset age less than 6 months.

(4) Autosomal, usually dominant. A child has 50% risk of inherit the disease with this mode of inheritance [29].

(5) Males are more severely affected [30]. A study could not exclude X-linked dominant inheritance [31]. These studies can justify the higher frequency of registered males with the disease.

(6) F:M 1:4, increasing in F with time [26]

(7) A French study reported a ratio of about 1:3 (F:M) in the molecular diagnosis of the disease, in line with our findings [32].

References

[26] Dutly F., Altwegg M. Whipple's Disease and "Tropheryma whippelii". *Clin Microbiol Rev* **2001**, *14,* 561–583.

[27] Pedrini E., Jennes I., Tremosini M., Milanesi A., Mordenti M., Parra A., Sgariglia F., Zuntini M., Campanacci L., Fabbri N., Pignotti E., Wuyts W., Sangiorgi L. Genotype-phenotype correlation study in 529 patients with multiple hereditary

[28] Hausmanowa-Petrusewicz I., Zaremba J., Borkowska J., Szirkowiec W. Chronic proximal spinal muscular atrophy of childhood and adolescence: sex influence. *J Med Genet* **1984,** *21* (6), 447-50.

[29] NIH-NCATS Best vitelliform macular dystrophy. Available online: https://rarediseases.info.nih.gov/diseases/182/best-vitelliform-macular-dystrophy (Accessed on November 2017)

[30] OMIM Entry #162500 NEUROPATHY, HEREDITARY, WITH LIABILITY TO PRESSURE PALSIES; HNPP Available online at: https://www.omim.org/entry/162500#clinicalFeatures (Accessed on 5 November 2017)

[31] Roos D., Thygesen P. Familial recurrent polyneuropathy: a family and a survey. *Brain* **1972**, *95,* 235-248.

[32] Biancalana V., Beldjord C., Taillandier A., Szpiro-Tapia S., Cusin V., Gerson F., Philippe C., Mandel J.-L., French National Working Group on Fragile X Syndrome. Five years of molecular diagnosis of fragile X syndrome (1997-2001): a collaborative study reporting 95% of the activity in France. *Am J Med Genet* ***2004**,* *129A* **,** 218-224.
