# Supplementary material for: The Occurrence of 275 Rare Diseases and 47 Rare Disease Groups in Italy. Results from the National Registry of Rare Diseases
Source: Int J Environ Res Public Health. 2018 Jul 12;15(7):1470. doi: 10.3390/ijerph15071470 (PMC6068991; doi:10.3390/ijerph15071470)
Supplement: Supplementary file 1 [file ijerph-15-01470-s001.zip › Table S7.pdf]

Article submitted to International Journal of Environmental Research and Public Health
The Occurrence of 275 Rare Diseases and 47 Rare Disease Groups in Italy. Results from the National Registry of Rare Diseases.
Domenica Taruscio*, Luciano Vittozzi, Adele Rocchetti, Paola Torreri, Luca Ferrari

Table S7. Incidence of rare diseases notified to RNMR during 2012-2014

Record source: Subset 1; **Notifying regions**: AD and CD Regions; **Record selection**: Records with date of diagnosis ascertainment or certification in the period 2012-2014; **Total records**: 63162.

Note: Records are grouped by exemption code, i.e.: diseases identified with group codes are collectively reported with the group denomination.

ad : value based on AD notifications
cd : value based on CD notifications
m : value based on both notifications

		Average population in the period 2012-2014 (million)	60.421	4.412	0.128	9.924	0.515	0.435	4.912	1.226	1.580	4.425
RNMR Code	ORPHA Code	Region<br>Disease or Disease group	Italy	Piemonte	Valle d'Aosta	Lombardia	PA Bolzano	PA Trento	Veneto	Friuli-Venezia Giulia	Liguria	Emilia-Romagna
RA0010	548	Hansen disease (Leprosy)	0.03cd	0	0	0	0	0	0.07cd	0	0.63cd	0
RA0020	3452	Whipple disease	0.09m	0.23ad	0	0.07ad	0.65cd	0.77cd	0.14cd	0	0	0.15cd
RA0030	91546	Lyme disease	0.33m		0		0	0.77cd	0.2cd	8.43cd	0.42cd	0.15cd
RB0010	654	Wilms tumor	0.66m	0.15ad	0	0.03ad	0	1.53cd	1.02cd	0.27cd	0	0.08cd
RB0020	790	Retinoblastoma	0.83m	0.53ad	2.6ad	0.67ad	0	1.53ad				0.68ad
RB0040	79665	Gardner syndrome	0.03m	0	0	0	0	0	0	0	0	0
RB0050	733	Familial adenomatous polyposis	1.9m	0.91ad	0	0.2cd	4.53cd	0.77cd	1.7cd	5.44cd	2.95cd	3.24cd
RB0060	538	Lymphangioleiomyomatosis	0.34m	0.38ad	0		0.65cd		0.27cd	0.27cd	0.63cd	
RC0010	199296	Congenital isolated ACTH deficiency	0.28m		0		0	0.77cd	0.27cd	0.27cd	0	0.9cd
RC0020	478	Kallmann syndrome	0.94m	1.13ad	0		3.24cd	2.3cd			0.42cd	1.51cd
RC0030		Reifenstein syndrome	0.04m	0	0	0	0	0	0	0	0	0
RC0040	169615	Idiopathic central precocious puberty	7.33m	3.25ad	2.6ad		6.48cd	15.34cd	11.6cd	5.71cd		19.51cd

RC0050	508	Leprechaunism	0.01ad	0	0	0	0	0	0	0	0	0
RC0060	902	Werner syndrome	0.02m	0	0	0	0	0	0.14cd	0	0.21cd	0
RC0080		Total lipodystrophy	0.1m	0.08ad	0	0.07cd	0	0	0.07cd	0	0	0.3cd
RC0090	36397	Dercum disease	0.09m	0ad	2.6ad	0	0	0	0	0		0.23cd
RC0100	333	Farber Disease	0.01m	0	0	0.03ad	0	0	0	0	0	0
RC0110		Mixed cryoglobulinemia	1.75m	0.83ad	0	1.91ad	0	0	1.49cd	11.42cd		1.96cd
RC0120	48818	Aceruloplasminemia	0.01ad	0	0	0.03ad	0	0	0	0	0	0
RC0150	905	Wilson disease	0.7m	0.6ad	2.6ad		1.3cd	1.53cd	0.88cd	0.54cd	0.63cd	0.6cd
RC0160	436	Hypophosphatasia	0.02m	0.08ad	0	0	0	0	0	0	0	0
RC0170		Vitamin D-resistent hypophosphatem	0.45m	0.38ad	0		0	0		0	0	0.45cd
RC0180	205	Crigler-Najjar syndrome	0.08m	0	0	0.07ad	0.65ad	0	0	0	0.21cd	
RC0190	91378	Hereditary angioedema	0.81m	0.6ad	0	0	5.18cd	0	1.7cd		1.48cd	0.15cd
RC0200	60	Congenital alpha-1-antitrypsin deficie	0.61m	0.38ad	0	0.47ad	17.49cd	2.3cd	1.36cd			
RC0210	117	Behçet disease	4.57m	4.16ad	0			9.21cd	6.45cd	13.59cd		
RD0010		Hemolytic-uremic syndrome	0.53m	0.45ad	0	0.87ad	0	0	1.02cd	0.54cd	0.21ad	
RD0020	447	Paroxysmal nocturnal hemoglobinuri	0.24m	0.38ad	0	0.24ad	0	0		0	0	0.53cd
RD0030	761	Henoch-Schönlein purpura	1.35m	1.51ad	0		0	0.77cd	1.02cd	1.63cd	0.42cd	1.58cd
RD0040	2686	Cyclic neutropenia	0.13m		0	0.03cd	0	0.77cd	0.14cd	0	0.42cd	0.08cd
RD0050	379	Chronic granulomatous disease	0.19m	0	0	0.1ad	0	0	0.07ad	0.27cd	0	0
RF0010	726	Alpers syndrome	0.04m	0.08ad	0	0.03ad	0	0	0	0.27cd	0	0.23cd
RF0020	480	Kearns-Sayre syndrome	0.31m	0.08ad	0		0	0		0		0.68cd
RF0030	506	Leigh syndrome	0.25m	0.08ad	0	0.44ad	0	0	0.2cd	0.54cd		0.68cd
RF0040	778	Rett syndrome	0.53m	0.3ad	0	0.64ad	1.3cd			0	0.42cd	
RF0050	101	Dentatorubral pallidoluysian atrophy	0.01ad	0	0	0	0	0	0	0	0	0
RF0060	98261	Progressive myoclonic epilepsy	0.21m	0.38ad	0	0	0	0	0.07cd	0.27cd	1.69cd	0.08ad
RF0070	36899	Myoclonus-dystonia syndrome	0.02m	0.23ad	0	0	0	0	0	0	0.21cd	0
RF0080	399	Huntington disease	3.38m	2.64ad	5.2ad	1.48ad	5.18cd	4.6cd	2.44cd	4.08cd		3.16cd
RF0090		Idiopathic torsion dystonia	1.46m	0.98ad	0	0.4ad	0.65cd	0	2.44cd	0.82cd	1.05cd	1.96cd
RF0100	803	Amyotrophic lateral sclerosis	19.15m	23.72ad	10.4ad	15.01ad	20.73cd	39.13cd	29.99cd	22.02cd	41.77cd	29.91cd
RF0110	35689	Primary lateral sclerosis	0.56m	1.28ad	0	0.44ad	0	0.77ad	0.41cd	0.54cd	0.84cd	
RF0120		Adrenoleukodystrophy	0.2m	0.15ad	0	0.2ad	0	0.77cd	0.27cd	0.27cd	0	0.15ad
RF0130	2382	Lennox-Gastaut syndrome	0.59m		2.6cd		1.3cd	0.77cd	0.48cd	0	1.27cd	3.01cd
RF0140	3451	West syndrome	0.7m	0	0	0.3ad	0	0	0.41cd	1.36cd	1.69cd	0.75cd
RF0150	2073	Narcolepsy	0.97m		0		1.3cd	0.77cd	0.95cd	1.36cd		2.49cd
RF0160	2483	Melkersson-Rosenthal syndrome	0.05m	0	0	0	0.65cd	0	0	0	0.42cd	0.08cd
RF0170	240071	Steele-Richardson-Olszewski disease	0.63m	0.68ad	0	0.74ad	0.65cd	0	0.95cd	0	0.84cd	0.23cd
RF0180	2932	Chronic inflammatory demyelinating	3.77m	5.29ad	2.6cd	3.29ad	3.24cd	2.3cd	6.65cd	1.09cd	7.17cd	11cd

RF0190	43393	Lambert-Eaton syndrome	0.13m	0	0		0	0	0.14cd	0		0.38cd
RF0200	891	Familial exudative vitreoretinopathy	0.31m		0		0.65cd			0.54cd	0	
RF0210	40923	Eales disease	0.03m	0	0	0	0	0	0.07cd	0	0	0
RF0230	263479	Fuchs heterochromic iridocyclitis	0.34m	0.08ad	0	0.1ad	0	0.77cd	0.75cd	0.54cd	0	0.23cd
RF0240	98981	Essential iris atrophy	0.01m	0	0	0.03ad	0	0	0	0	0	0
RF0250	215	Congenital stationary night blindness	0.01cd	0	0	0	0	0	0	0	0	0
RF0270	1467	Cogan syndrome	0.16m	0.08ad	0	0.1cd	0.65cd	0.77cd	0.07cd	0.54cd	0.21cd	0.68cd
RF0280	156071	Keratoconus	25.53m	11.86ad		8.13ad	174.89cd	58.3cd	61.62cd	14.68cd	17.09cd	36.99cd
RF0290	97231	Ligneous conjunctivitis	0.01ad	0	0	0	0	0	0	0	0	0
RF0300	104	Leber hereditary optic neuropathy	0.79m		0		0	0	1.09cd	1.63cd	0.21cd	
RG0010		Rheumatic endocarditis	1.36m	1.13ad	0	0.27ad	0	4.6cd	0.68cd	0.27cd	1.05cd	2.86cd
RG0020	727	Microscopic polyangiitis	1.14m	2.8ad	0	1.18ad	0.65cd	0.77cd	0.41cd	3.26cd	3.59cd	2.03cd
RG0030	767	Polyarteritis nodosa	0.41m	0.45ad	0		0.65cd	0	0.54cd	1.36cd	1.27cd	0.9cd
RG0040	2331	Kawasaki disease	2.08m	1.44ad	0	1.55ad	1.3cd	1.53cd	3.73cd	2.17cd	1.05cd	2.94cd
RG0050	183	Churg-Strauss syndrome	1.88m	1.89ad	0	1.48ad	5.83cd	1.53cd	2.92cd	3.26cd		3.24cd
RG0060	375	Goodpasture syndrome	0.04m	0.15ad	0	0.03ad	0	0	0	0	0	0
RG0070	900	Wegener granulomatosis	1.77m		0	0.97ad	9.07cd	9.97cd	2.92cd	7.61cd		3.62cd
RG0080	397	Giant cell arteritis	3.73m	7.63ad	0	4.4ad	7.77cd	9.21cd	3.53cd	17.4cd		7.61cd
RG0090	3287	Takayasu arteritis	0.63m	0.98ad	0		1.3cd	0.77cd	0.75cd	0.54cd	0.84cd	1.81cd
RG0100	774	Hereditary hemorrhagic telangiectasi	2.36m	1.06ad	0		1.94cd					
RG0110	131	Budd-Chiari syndrome	0.13m	0	0	0.03ad	0	0.77ad	0.27cd	0	0	0.23cd
RI0010	930	Idiopathic achalasia	4.63m	1.81ad	0			3.07cd	6.72cd	2.72cd		7.01cd
RI0030	2070	Eosinophilic gastroenteritis	0.55m	1.36ad	0	0.71ad	0	1.53cd	0.48cd	2.45cd	0.63cd	1.21cd
RI0040		Chronic intestinal pseudoobstruction	0.23m	0.53ad	0		0	0	0.54cd	0.54cd	0	0.3cd
RI0050	171	Primary sclerosing cholangitis	1.03m	1.44ad	2.6ad		3.89cd	0.77cd	1.97cd	2.99cd		2.56cd
RI0070	2290	Microvillus inclusion disease (MVID)	0.02m	0.08ad	0	0	0	0	0	0	0	0.08cd
RI0080	36204	Intestinal lymphangiectasia	0.07m	0.15ad	0	0	0.65cd	0.77cd	0.07cd	0	0	0.15cd
RJ0010	223	Nephrogenic diabetes insipidus	0.07m	0	0	0	0	0	0.34cd	0	0	0.08cd
RJ0020	49041	Retroperitoneal fibrosis	0.72m	1.74ad	0		2.59cd	2.3cd	0.68cd	1.9cd	1.05cd	0.9cd
RJ0030	37202	Interstitial cystitis	3.19m	1.36ad	0	1.44ad	1.94cd		2.99cd			
RL0020	1656	Dermatitis herpetiformis	0.66m	0.91ad	0	0.4ad	1.3cd	5.37cd	1.36cd	2.45cd	1.48cd	1.28cd
RL0030		Pemphigus vulgaris	3.75m	4.46ad	2.6ad	2.45ad	2.59cd	4.6cd	4cd	7.07cd	7.38cd	6.03cd
RL0040		Bullous pemphigoid	4.88m	6.88ad	0	4.53ad	8.42cd	3.84cd	7.46cd	14.68cd	4.64cd	7.53cd
RL0050	46486	Mucous membrane pemphigoid	0.68m		0		0	1.53cd		4.08cd	2.74cd	0.9cd
RL0060		Lichen sclerosus	5.92m	9.52ad	2.6cd		5.18cd	30.69cd	4.34cd	5.71cd	14.56cd	18.76cd
RM0010	221	Dermatomyositis	1.87m	1.44ad	0		5.83cd	6.14cd	2.92cd	3.81cd	2.32cd	4.14cd
RM0020	732	Polymyositis	1.95m	1.59ad	0		2.59cd	3.07cd	2.99cd	7.07cd	2.74cd	3.47cd

RM0030	809	Mixed connective tissue disease	1.8m	2.04ad	2.6ad	1.34ad	4.53cd	0	2.04cd	2.72cd	4.43cd	2.34cd
RM0040	3165	Eosinophilic fasciitis	0.2m	0.45ad	0	0.07ad	0	0.77cd	0.34cd	0.27cd		0.08cd
RM0050		Diffuse fasciitis	0.03m	0	0	0.03ad	0	0	0	0	0	0
RM0060	728	Relapsing polychondritis	0.18m	0.08ad	0		0.65cd	0.77cd	0.41cd	0.27cd	0.42cd	0
RN0010		Arnold-Chiari malformation	4.19m	7.48ad	0	2.86ad	5.83cd	8.44cd	8.96cd	4.62cd	8.65cd	4.97cd
RN0020	199642	Microcephaly	0.35m	0.08cd	0	0.07ad	1.3cd	0.77cd	0.14cd	0	0	0.38cd
RN0030		Cerebellar agenesis	0.04m	0	0	0.03ad	0	0	0.07cd	0.27cd	0	0.08cd
RN0040	475	Joubert syndrome	0.15m	0.08ad	0	0.17ad	0.65cd	0	0.34cd	0.27cd	0.21cd	0
RN0050		Lissencephaly	0.18m		0	0.1ad	0.65cd	0	0.2cd	0	0	
RN0060	2162	Holoprosencephaly	0.08m	0.15ad	0	0	0	1.53cd	0	0	0	0
RN0080	1764	Familial dysautonomia	0.03m	0	0	0	0.65cd	0	0	0	0	0
RN0090		Axenfeld-Rieger anomaly	0.04m	0.08ad	0	0.07ad	0	0	0.2cd	0	0	0
RN0100	708	Peters anomaly	0.02m	0	0	0	0	0	0.14cd	0	0	0.08cd
RN0110	77	Aniridia	0.11m	0.08ad	0	0.03ad	0	0	0.14cd	0.27cd	0	0.3cd
RN0120		Congenital colobomatous optic disc	0.31m	0.15ad	0		1.3cd	2.3cd		0	0.84cd	
RN0130	35737	Morning glory syndrome	0.02m	0	0	0.03ad	0	0	0	0	0	0
RN0140		Pupillar membrane persistence	0.01cd	0	0	0	0	0	0	0	0	0.08cd
RN0150	1059	Blue rubber bleb nevus	0.02m	0	0	0.03ad	0	0	0	0	0	0
RN0160		Esophageal atresia and/or Isolated tra	0.66m	1.21ad	2.6ad		0	0	1.63cd	1.63cd	1.9cd	0.9cd
RN0170	1201	Atresia of small intestine	0.18m	0.68ad	0	0.03ad	0	0.77cd	0.27cd	0.54cd	0.84cd	0.15cd
RN0180	1203	Duodenal atresia	0.16m	0.83ad	0	0.13ad	0	0	0.14cd	0	0.42cd	0.45cd
RN0190	96346	Anorectal malformation	0.93m	1.28ad	2.6ad		2.59cd	5.37cd	2.58cd	1.36cd	3.8cd	1.73cd
RN0200	388	Hirschsprung disease	0.88m		2.6cd		1.3cd	3.07cd	1.36cd	1.63cd	2.74cd	1.43cd
RN0210		Biliary atresia	0.44m	0.15ad	0	0.24ad	0	0	0.68cd	1.63cd	1.05cd	
RN0220	53035	Caroli disease	0.13m	0.15ad	0	0.1ad	0	0	0.34cd	0	0.42cd	0.08cd
RN0230	2924	Polycystic liver disease	0.42m	2.12ad	0	0.3ad	3.24cd	0.77cd	1.22cd	0	0	0.23cd
RN0240	2138	True hermaphroditism	0.01ad	0.08ad	0	0	0	0	0	0	0	0
RN0250	1309	Medullary sponge kidney	0.3m	1.06ad	0	0.34ad	0	0.77cd	0.54cd		0.21cd	0.53cd
RN0260	294975	Phocomelia	0.06cd	0.15cd	0	0.03cd	0	0	0	0	0.21cd	0
RN0270	3181	Sprengel deformity	0.02cd	0	0	0	0	0	0.14cd	0	0	0
RN0280	950	Acrodysostosis	0.01ad	0	0	0	0	0	0	0	0	0
RN0290		Camptodactyly	0.02m	0	0	0	0	0	0.07cd	0	0	0.08cd
RN0300	3027	Caudal regression sequence	0.09m	0.15cd	0	0.07cd	0	0	0.07cd	0	0.42cd	0.08cd
RN0310	2345	Isolated Klippel-Feil syndrome	0.17m	0.53ad	0		0.65cd	0.77cd	0.14cd	0.54cd	0	0.3cd
RN0320	2368	Gastroschisis	0.13m	0	0		0	0	0.2cd	0.27cd	0.42cd	0.3cd
RN0330	98249	Ehlers-Danlos syndrome	2.43m	0.76ad	0	4.03ad	3.24cd	0.77ad			1.27cd	
RN0340	974	Adams-Oliver syndrome	0.03m	0	0	0.03ad	0	0	0.07cd	0	0	0

RN0350	192	Coffin-Lowry syndrome	0.02m	0	0	0.03ad	0.65cd	0	0	0	0	0
RN0360	1465	Coffin-Siris syndrome	0.06m	0	0	0	0	0	0	0	0	0
RN0370	239	Dyggve-Melchior-Clausen disease	0.01m	0	0	0.03ad	0	0	0	0	0	0
RN0380	3255	Filippi syndrome	0.01ad	0	0	0	0	0	0	0	0	0
RN0390	380	Greig cephalopolysyndactyly syndrom	0.03m	0	0	0	0	0	0.14cd	0	0.21cd	0
RN0400	1540	Jackson-Weiss syndrome	0.02m	0	0	0	0	0	0.07ad	0	0	0
RN0410	2311	Jarcho-Levin syndrome	0.03m	0.08cd	0	0	0	0	0	0	0	0
RN0430	2911	Poland syndrome	1.14m		0		0	0.77cd	1.09cd	1.9cd	3.38cd	1.51cd
RN0470	669	Otopalatodigital syndrome	0.02m	0	0	0	0	0	0	0	0	0
RN0480	3377	Trismus-pseudocamptodactyly	0.01cd	0	0	0	0	0	0	0.27cd	0	0
RN0500		Cutis laxa	0.01m	0	0	0	0	0	0.07cd	0	0	0
RN0510	464	Incontinentia pigmenti	0.12m	0.23ad	0	0	0	0	0.14cd	0	0	0.68cd
RN0520	910	Xeroderma pigmentosum	0.04m	0	0	0.03ad	0	0	0.07cd	0	0	0.08cd
RN0530		Keratosis follicularis spinulosa decalva	0.01ad	0	0	0	0	0	0	0	0	0
RN0540	1556	Cutis marmorata telangiectatica cong	0.06m	0.08ad	0	0	0.65cd	0	0.2cd	0	0	
RN0550	218	Darier disease	0.63m	0.98ad	0		0.65cd	3.84cd	0.95cd	1.36cd	0.42cd	1.51cd
RN0560	1775	Dyskeratosis congenita	0.02ad	0.08ad	0	0	0	0	0	0	0	0
RN0570		Epidermolysis bullosa	0.55m	0.3ad	0	0.03ad	0.65cd	1.53cd	0.48cd	1.36cd	0.63cd	0.6cd
RN0580	316	Progressive symmetric erythrokerato	0.01m	0	0	0	0	0	0	0.27cd	0	0
RN0590	317	Erythrokeratodermia variabilis	0.02m	0.08ad	0	0	0	0	0	0	0	0.15cd
RN0600	312	Epidermolytic ichthyosis	0.06m	0	0	0	0	0	0.07cd	0	0	0.08cd
RN0610	2092	Focal dermal hypoplasia	0.03m	0	0	0	0	0	0	0	0.42cd	0.08cd
RN0620	2796	Pachydermoperiostosis	0.02cd	0	0	0	0	0	0	0.27cd	0	0
RN0630	758	Pseudoxanthoma elasticum	0.36m	0.15ad	0	0.03ad	1.94cd	2.3cd	0.75cd	0		0.6cd
RN0640	1114	Aplasia cutis congenita	0.02m	0	0	0	0	0	0	0	0	0.08cd
RN0650	1214	Parry-Romberg syndrome	0.13m	0.15ad	0	0.07cd	0	0	0.61cd	0.27cd	0	0
RN0660	870	Down syndrome	7.76m	4.23ad	0		24.61cd	6.9cd	6.58cd	2.17cd	3.8cd	1.88cd
RN0670	281	Cri du chat syndrome	0.15m	0	0	0.07ad	0	0	0.27cd	0	0.42cd	0.15cd
RN0680	881	Turner syndrome	2.53m	1.89ad	2.6ad		2.59cd	3.07cd	1.97cd	1.9cd	5.91cd	1.88cd
RN0690		Klinefelter syndrome	3.75m	2.87ad	0		7.77cd	6.9cd	6.38cd	4.08cd	3.8cd	8.66cd
RN0700	280	Wolf-Hirschhorn syndrome	0.13m	0	0		0	0.77cd	0	0	0.21cd	0.15cd
RN0710	550	MELAS	0.73m	0.83ad	0	0.57ad	0	0.77cd		0.54cd		
RN0720	551	MERRF	0.12m	0.08ad	0	0.3ad	0	0	0	0	0	
RN0740		Ivemark syndrome	0.03cd	0	0	0	0	0	0.27cd	0	0	0
RN0750	805	Tuberous sclerosis	1.18m	0.83ad	0	0.4ad	1.3cd	1.53cd	1.22cd	1.36cd	1.9cd	1.58cd
RN0760	2869	Peutz-Jeghers syndrome	0.2m	0.08ad	0	0.07ad	1.3cd	0	0.61cd	0.27cd	0.63cd	0
RN0770	3205	Sturge-Weber syndrome	0.16m		0	0	0	0	0.27cd	0.54cd	0.42cd	0.23cd

RN0780	892	Von Hippel-Lindau disease	0.22m	0.15ad	0		0.65cd	0.77cd	0.14cd	0	0.42cd	0.15cd
RN0790	915	Aarskog-Scott syndrome	0.09m	0.08ad	0	0.03ad	0	0	0.07cd	0.27cd	0	0.08cd
RN0800	83	Antley-Bixler syndrome	0.01ad	0	0	0	0	0	0	0	0	0
RN0810	1225	Baller-Gerold syndrome	0.01ad	0	0	0	0	0	0	0	0	0
RN0820	116?	Beckwith-Wiedemann syndrome	0.76m	0.91ad	0		1.3cd	0.77cd	0.88cd	0.82cd	0.21cd	0.45cd
RN0830	125	Bloom syndrome	0.01cd	0	0	0	0	0	0	0	0	0
RN0850	138	CHARGE syndrome	0.35m	0.15ad	0		0.65cd	0.77cd	0.54cd		0.42cd	0.6cd
RN0860	3157	De Morsier syndrome	0.13m	0.53ad	0	0.03ad	1.3cd	0	0.14cd	0	0.42cd	0.08cd
RN0870	235	Dubowitz syndrome	0.03cd	0	0	0	0	0	0.07cd	0	0	0
RN0880	1896	EEC syndrome	0.37m	0.53ad	2.6ad		1.3cd	2.3cd	0.75cd	0	0	0.68cd
RN0890	2053	Freeman-Sheldon syndrome	0.03m	0	0	0	0	0	0.2cd	0	0	0.08cd
RN0900	2059	Fryns syndrome	0.01cd	0	0	0	0	0	0.07cd	0	0	0
RN0910	374	Goldenhar syndrome	0.62m		0		1.94cd	0.77cd	0.54cd	0.82cd	1.69cd	0.75cd
RN0930	392	Holt-Oram syndrome	0.06m	0	0	0	0	0	0	0.27cd	0.21cd	0.08cd
RN0940	2322	Kabuki make-up syndrome	0.31m	0.23ad	2.6ad	0.1ad	0	0	0.14cd	0	0.63cd	0.08cd
RN0950	98861	Primary ciliary dyskinesia, Kartagener	0.56m	0.91ad		0.54ad	0.65cd			0	0.84cd	
RN0960	163634	Maffucci syndrome	0.05m	0	0	0.03ad	0	0	0.2cd	0.27cd	0	0.08cd
RN0970	560	Marshall syndrome	0.01ad	0	0	0	0	0	0	0	0	0
RN0980	564	Meckel Syndrome	0.01cd	0	0	0	0	0	0	0	0	0
RN0990	570	Moebius syndrome	0.13m	0	0	0	0	0	0.2cd	0	0	0.08cd
RN1000	245	Nager syndrome	0.01cd	0	0	0	0	0	0.07cd	0	0	0
RN1010	648	Noonan syndrome	1.3m	1.28ad	0		3.24cd	0	1.43cd	2.45cd		2.11cd
RN1020		Opitz syndrome	0.04m	0	0	0	0	0	0	0	0	0.08ad
RN1040	710	Pfeiffer syndrome	0.03m	0.15ad	0	0	0	0	0	0	0	0.08cd
RN1070	97360	Robinow syndrome	0.01cd	0	0	0	0	0	0.07cd	0	0	0
RN1080	813	Silver-Russell syndrome	0.29m	0.15ad	0	0	0	0	0.41cd	0	0	0.3cd
RN1100	808	Seckel syndrome	0.02m	0	0	0.03ad	0	0	0.07cd	0	0	0
RN1120		Simpson-Golabi-Behmel syndrome	0.02m	0.08ad	0	0	0	0	0	0	0	0
RN1130	1297	Branchio-oculo-facial syndrome	0.02m	0	0	0	0	0	0	0	0	0.08cd
RN1140	107	Branchio-oto-renal syndrome	0.09m	0	0	0.03ad	0.65cd	0.77cd	0	0.27cd	0	0.3cd
RN1150	1340	Cardiofaciocutaneous syndrome	0.13m	0	0	0.1ad	0	0.77cd	0.14cd	0	0	0
RN1170	744	Proteus syndrome	0.08m	0.08ad	0	0.03ad	0	0	0.07cd	0	0.21cd	0
RN1180	324764	Trichorhinophalangeal syndrome	0.12m	0	0	0	0.65cd	0	0	0	0	0.15cd
RN1190	2614	Nail-patella syndrome	0.14m	0.08ad	0	0.1ad	0	0	0.27cd	0	0.21cd	0.23cd
RN1200		Smith-Lemli-Opitz syndrome type 1	0.04m	0.08ad	0	0	0	0	0	0	0.21cd	0
RN1210	819	Smith-Magenis syndrome	0.14m	0	0	0	0	0	0.27cd	0.54cd	0.21cd	
RN1220	828	Stickler syndrome	0.13m	0.45ad	0	0.03ad	0	0	0.14cd	0	0	0

RN1240	857	Townes-Brocks syndrome	0.01m	0	0	0	0	0.77cd	0	0	0	0
RN1250	887	VACTERL/VATER association	0.23m	0.15ad	0	0.17ad	0.65cd	0.77cd	0.2cd	0	0.63cd	0.3cd
RN1270	904	Williams syndrome	0.98m	0.45ad	0		0.65cd	0.77cd	0.81cd	2.17cd		1.28cd
RN1280	3460	Winchester syndrome	0.01ad	0	0	0	0	0	0.07ad	0	0	0
RN1290	3463	Wolfram syndrome	0.06m	0	0	0.07cd	0	0	0	0	0	0.08cd
RN1300	72	Angelman syndrome	0.38m	0.15ad	2.6cd	0.2ad	1.3cd	2.3cd	0.41cd	0.27cd	0	
RN1310	739	Prader-Willi syndrome	0.78m		2.6ad		1.3cd	3.07cd	1.02cd		1.27cd	0.6cd
RN1320	558	Marfan syndrome	1.79m	1.13ad	0	0.71ad	3.24cd	0.77ad		1.9cd	0.42cd	2.11cd
RN1330	908	Fragile X syndrome	1.15m	0.91ad	0	0.13ad	3.24cd	1.53cd	1.29cd	0.54cd	1.69cd	
RN1350		Alagille syndrome	0.18m	0.3ad	0	0.1ad	0.65cd	1.53cd	0.2cd	1.09cd	0.21cd	0.15cd
RN1360	63	Alport syndrome	0.61m	0.45ad	0	0.3ad	0	0.77cd		1.63cd		
RN1370	64	Alström syndrome	0.03m	0	0	0	0	0	0	0.54cd	0	0
RN1380	110	Bardet-Biedl syndrome	0.13m	0.15ad	0	0.03cd	0.65ad	0	0.2cd	0	0	0.08cd
RN1410	199	Cornelia de Lange syndrome	0.2m	0	0	0.17ad	1.3cd	0	0.14cd	0.27cd	0.21cd	0.23cd
RN1430	220	Denys-Drash syndrome	0.02cd	0	0	0	0	0.77cd	0	0	0	0
RN1440	2710	Oculodentodigital dysplasia	0.01cd	0	0	0	0	0	0	0	0	0
RN1450	94068	Congenital spondyloepiphyseal dyspla	0.08m	0.08ad	0	0.03cd	0	0	0.14cd	0	0	0.08cd
RN1460	2052	Fraser syndrome	0.01ad	0	0	0.03ad	0	0	0	0	0	0
RN1480	435	Ito hypomelanosis	0.1m	0.08ad	0	0.17ad	0	0	0	0	0	0.08cd
RN1490		Isaacs syndrome	0.06m	0.15ad	0	0	0.65cd	0	0	0	0	
RN1500	477	KID Syndrome	0.01ad	0	0	0	0	0	0	0	0	0
RN1510	90308	Klippel-Trénaunay syndrome	0.49m	0.38ad	0		0.65cd	0	0.68cd	0.82cd	0.42cd	0.98cd
RN1520	98818	Landau-Kleffner syndrome	0.02m	0.08ad	0	0	0	0	0.14cd	0	0	0
RN1530	500	LEOPARD syndrome	0.08m	0.08ad	0	0.1ad	0.65cd	0	0.07cd	0	0	0.08cd
RN1570	263440	Neuroacanthocytosis	0.02m	0	0	0	0	0	0.14cd	0	0	0.08cd
RN1580	649	Norrie disease	0.01ad	0	0	0	0	0	0	0	0	0
RN1590	884	Pallister-Killian syndrome	0.04m	0.08ad	0	0	0	0	0	0.27cd	0	
RN1600	699	Pearson syndrome	0.01cd	0	0	0	0	0	0	0	0.21cd	0
RN1610	2905	POEMS syndrome	0.04m	0.15ad	0	0.07ad	0	0		0	0	0
RN1620	783	Rubinstein-Taybi syndrome	0.16m	0.08ad	0	0.03ad	0.65cd	0	0.14cd	0.27cd	0.21cd	0.15cd
RN1640	1466	COFS syndrome	0.01cd	0	0	0	0	0	0	0	0	0
RN1650	404560	Familial dysplastic nevus syndrome	0.33m	0	0	0.13ad	4.53cd	0	0.07cd	0.27cd	1.48cd	0.15cd
RN1660	35125	Epidermal nevus syndrome	0.04m	0	0	0	0	0.77cd	0.07cd	0	0.21cd	0
RN1670		Multiple pterygium syndrome	0.02m	0	0	0	0	0	0	0	0	0
RN1680	3352	Tricho-dento-osseous syndrome	0.01cd	0	0	0	0	0	0	0	0	0
RN1690		Radio-ulnar synostosis-amegakaryocy	0.01cd	0	0	0	0	0	0	0	0	0
RN1700	816	Sjögren-Larsson syndrome	0.26m	0	0	0	0	0	0.07cd	2.99cd	0	0

RN1720	3437	Vogt-Koyanagi-Harada disease	0.2m	0.3ad	0	0.07cd	1.3cd	0.77cd	0.27cd	0	0.42cd	
RN1750	3449	Weill-Marchesani syndrome	0.02m	0	0	0	0	0	0	0	0	0
RN1760	912	Zellweger syndrome	0.04m	0	0	0.03cd	0	0	0	0	0	0
RP0010	290	Congenital rubella syndrome	0.17cd	0	0	0	0	0	0	0	0	0
RP0040	1915	Fetal alcohol syndrome	0.25m	0.15ad	0	0.03cd	0	0	0.07cd	0.82cd	0.42cd	
RP0050	70590	Infantile apnea	0.78m	0	0	0	0	0	0	0	0.21cd	0
RP0060	415286	Bilirubin encephalopathy	0.01cd	0	0	0	0	0	0	0	0	0
RP0070		Congenital hepatic fibrosis	0.06m	0.08ad	0	0.1ad	0	0	0.2cd	0.54cd	0	0
RN0460	1988	Femoral-facial syndrome	0.01cd	0	0	0.03cd	0	0	0	0	0	0
RN0730	3163	SHORT syndrome	0.01ad	0	0	0	0	0	0	0	0	0
RN1090	798	Schinzel-Giedion syndrome	0.01cd	0	0	0.03cd	0	0	0	0	0	0
RN1110		Fetal hypokinesia syndrome	0.01cd	0	0	0	0	0	0.07cd	0	0	0
RN1160	1647	Oculocerebrocutaneous syndrome	0.01cd	0	0	0	0	0	0	0	0	0
RN1630	36	Acrocallosal syndrome	0.01cd	0	0	0	0	0	0	0	0	0
RP0020	1906	Fetal valproate syndrome	0.01cd	0	0	0	0	0	0	0	0	0
RP0030	1912	Fetal hydantoin syndrome	0.01cd	0	0	0	0	0	0	0	0	0
RBG010		Neurofibromatoses	10.08m	9.97ad	2.6cd		19.43cd	9.97cd	13.03cd	20.93cd	7.17cd	8.14cd
RCG010		Primary hyperaldosteronisms	1.35m	1.74ad	0	0.5ad	3.24cd	1.53cd	2.44cd	1.63cd		2.94cd
RCG020		Congenital adrenogenital syndromes	2.17m	0.91ad	0		3.89cd	7.67cd	1.22cd	3.26cd	1.69cd	3.84cd
RCG030		Autoimmune polyendocrinopathies	2.58m	2.49ad	0	2.55ad	7.13cd	1.53cd	1.43cd	1.63cd	2.53cd	6.03cd
RCG040		Disturbances of aminoacid transport a	7.92m	4.08ad	5.2ad	3.09ad	5.83cd		5.09cd	7.88cd	16.88cd	6.48cd
RCG050		Urea cycle disturbances	0.38m	0.23ad	5.2ad		0	0	0.41cd	0.82cd	0.84cd	0.08ad
RCG060		Disturbances of carbohydrate transpo	1.69m	1.21ad	0		1.3cd	0.77cd	3.26cd			1.13cd
RCG070		Congenital alterations of lipoprotein r	1.37m	1.21ad	0	0.91ad		3.84cd	1.36cd	3.81cd	3.16cd	2.56cd
RCG080		Lipid storage disturbances	1.77m	1.44ad	0		0.65cd		1.49cd	1.36cd	1.69cd	3.77cd
RCG090		Mucolipidoses	0.04m	0	0	0.03cd	0	0	0.07cd	0	0	0
RCG100		Congenital alterations of iron metabo	8.04m	18.21ad	0	1.98ad	25.26cd	16.88cd	22.12cd	4.62cd	13.29cd	17.1cd
RCG110		Porphyrias	0.73m		2.6ad			0.77ad			0.21cd	
RCG120		Disorders of purine and pyrimidine m	0.04m	0	0	0.03ad	0	0	0	0	0.21cd	0
RCG130		Primary and familiary amyloidoses	1.81m	4.38ad	0	1.48ad	0.65cd					
RCG140		Mucopolysaccharidoses	0.38m	0.38ad	0		0.65cd	1.53cd	0.34cd	0.54cd	0.63cd	0.3cd
RCG150		Chronic histiocytoses	1.27m	1.44ad	0	0.3ad	0.65cd	0	1.56cd	0.27cd	1.48cd	2.49cd
RCG160		Primary immunodeficiencies	6.36m	4.16ad	2.6ad		7.77cd		9.84cd	15.5cd	11.18cd	7.16cd
RDG010		Hereditary anemias	15.04m	11.56ad	2.6ad		18.78cd		24.97cd	3.26cd	52.32cd	26.59cd
RDG020		Hereditary coagulation defects	25.16m	31.81ad		11.42ad	20.08cd	23.01cd	20.97cd	3.53cd	33.33cd	24.18cd
RDG030		Hereditary thrombocytopathies	0.49m	0.45ad	0	0.1ad	0.65cd	0.77cd	0.54cd	0.54cd	1.27cd	0.53cd
RDG040		Primary hereditary thrombocytopenia	0.44m	0.3ad	0	0.57ad	0	0	0	1.09ad	0	

RFG010	Leucodystrophies	0.61m	0.6ad	0		0.65cd	3.07cd	1.36cd	1.36cd	0.21cd	0.45cd
RFG020	Ceroid lipofuscinoses	0.1m	0	0	0	0.65cd	0.77cd		0.27cd	0.21ad	0
RFG030	Gangliosidoses	0.1m	0.08ad	0	0.03ad	0	0	0.14cd	0.27cd	0	0.08cd
RFG040	Spinocerebellar diseases	6.52m	3.85ad	2.6ad	3.59ad	2.59cd					
RFG050	Spinal muscular atrophies	1.59m	1.06ad	2.6ad	0.77ad	2.59cd	3.07cd	2.44cd	2.72cd		1.36cd
RFG060	Hereditary neuropathies	6.61m	6.95ad	0			15.34cd	8.89cd	3.26cd	12.02cd	7.01cd
RFG070	Hereditary congenital miopathies	1.31m	0.98ad	0	0.4ad	1.94cd		0.88cd	0.27cd		
RFG080	Muscular dystrophies	8.12m	2.87ad	2.6ad		3.89cd	6.9cd	7.87cd	11.96cd	6.12cd	5.65cd
RFG090	Miotonic dystrophies	4.44m	5.36ad	0	1.34ad	3.89cd	4.6cd		5.71cd		4.52cd
RFG100	Normo-, hypo- and hyperkalemic para	0.15m	0.15ad	0	0.1ad	0	0	0.07cd	0		0.23cd
RFG110	Hereditary retinic dystrophies	17.6m	10.58ad	0							
RFG120	Hereditary choroid dystrophies	0.41m		0	0.07ad	0	0	0.27cd	0.27cd	0	0.45cd
RFG130	Corneal degenerations	0.18m	0.08ad	2.6cd	0.03cd	3.24cd	0	0.34cd	0.27cd	0	0.08cd
RFG140	Hereditary corneal dystrophies	1.1m		0		1.94cd	2.3cd	3.8cd	1.09cd	0.84cd	2.03cd
RGG010	Thrombotic microangiopathies	1.16m	0.53ad	0		1.3cd	2.3cd	1.22cd	0	7.81cd	
RMG010	Undifferentiated connective tissue sy	17.75m	12.09ad	0	17.57ad	10.36cd	16.88cd	23.07cd	54.92cd	40.71cd	28.78cd
RNG010	Pseudohermaphroditisms	0.47m	0.23ad	0	0.2ad	0.65cd	0		0		0.23cd
RNG020	Multiple congenital arthrogryposes	0.16m	0.15ad	0	0	0.65cd	0	0.14cd	0.27cd	0	0.23cd
RNG030	Acrocephalosyndactyly	0.04m	0	0	0	0.65cd	0	0.07cd	0	0	0.08cd
RNG040	Congenital craniofacial anomalies	3.81m		0		9.07cd	14.58cd	15.27cd	2.45cd	4.64cd	7.31cd
RNG050	Congenital chondrodystrophies	2.56m		0		1.94cd	1.53cd	3.39cd	2.17cd	5.48cd	3.31cd
RNG060	Congenital osteodystrophies	3.37m	5.59ad	0		4.53cd	4.6cd	3.87cd	3.26cd	2.95cd	3.47cd
RNG070	Congenital ichthyoses	1.74m	2.27ad	2.6ad	0.07ad	1.3cd		1.7cd	0	1.48cd	1.58cd
RNG080	Chromosomal aneuploidy syndromes	1.27m		0	0.74ad	3.24cd	0	0.75cd	0		
RNG090	Chromosomal duplication/deficiency	7.82m	3.48ad	0	3.33ad	20.73cd	13.81cd	11.33cd	3.53cd		7.46cd
RNG100	Other congenital anomalies with intel	3.18m	1.36ad	0	0.97ad	0	0	0.81cd			

Supplementary File 4 - Table S4.1

RNMR Code	ORPHA Code	Toscana	Umbria	Marche	Lazio	Abruzzo	Molise	Campania	Puglia	Basilicata	Calabria	Sicilia	Sardegna
**Average population in the period 2012-2014 (million)**		3.732	0.893	1.550	5.773	1.326	0.314	5.834	4.077	0.577	1.972	5.062	1.656
RA0010	548	0	0	0	0	0	0	0	0.08cd	0	0	0	0
RA0020	3452	0	0	0	0.06ad	0	0	0	0.08cd	0	0	0.13ad	0.2ad
RA0030	91546	0.09ad	0	0.43ad	0.46ad	0	0	0	0.08cd	0	0	0.07ad	0
RB0010	654	0.98ad		0.22cd	0.98ad	2.01cd	3.19cd	1.2cd		0.58cd	0.17ad	0.07ad	0
RB0020	790	0.09ad	0.37ad	0	0.92ad	7.04ad	0			0.58ad		0.72ad	0.6ad
RB0040	79665	0	0.75cd	0	0.12ad	0	0	0	0.16cd	0	0	0	0
RB0050	733	0.36ad	1.49ad	1.51ad		1.51ad	1.06cd	2.34cd	8.75cd	4.04cd	0.17ad		1.61ad
RB0060	538	0.54ad	0.37ad	1.51ad		0	0			0	0.17cd		0
RC0010	199296	0	1.12cd	0	0.06ad	0.25ad	0	0.4cd	0.25cd	0			
RC0020	478	0.45ad	1.12cd	0.65ad		0.25ad	0	2.51cd		0	0.51ad		0
RC0030		0.09ad	0	0	0.12ad	0	0	0.17cd	0.08cd	0	0	0	0
RC0040	169615	8.57ad	23.15cd	8.6ad	5.77ad		22.31ad	6.17cd	11.28cd			3.75ad	4.03ad

RC0050	508	0	0	0.22ad	0	0	0	0	0	0	0	0	0
RC0060	902	0	0	0	0.06ad	0	0	0	0	0	0	0	0
RC0080		0.09ad	0.37ad	0	0.06ad	0.25ad	0	0	0.16cd	0	0	0.33ad	0
RC0090	36397	0.09ad	0	0	0.12ad	0	1.06ad	0.06cd	0	0	0	0	0.2ad
RC0100	333	0	0	0	0	0	0	0	0	0.58cd	0	0	0
RC0110		3.66ad		0.65ad	3ad	0.25ad	1.06ad		1.39cd	0.58ad			0.2cd
RC0120	48818	0	0	0	0	0	0	0	0	0	0	0	0
RC0150	905			0				1.89cd		0		0.46ad	0.2ad
RC0160	436	0.09ad	0	0	0	0	0	0	0.08cd	0	0	0	0
RC0170		0.54ad	0.75cd	0.43ad	0.12ad	0	0			0.58ad	0.34ad		0
RC0180	205	0.36ad	0	0	0.06ad	0	0	0	0	0	0	0	0
RC0190	91378		0.37cd	1.08ad	0.4ad	0.75ad	0	0	2.78cd	1.73cd	0.17cd	0.99ad	0.2ad
RC0200	60	0.71ad		0	0.06ad	0.25ad	0	0	0.65cd	0	0	0.07ad	0
RC0210	117	7.59ad			3.18ad				5.8cd	0.58ad	3.38ad		1.81ad
RD0010		1.43ad	0.75cd		0.29ad	0	0	0.51cd	0.16cd	0.58ad	0.17cd	0.07ad	0
RD0020	447	0.18ad	0.37cd	0.22ad	0.46ad	0	0	0	0.41cd	0	0	0	0.4ad
RD0030	761		2.24cd	0.86ad	5.54ad	1.01ad	1.06cd			1.16ad		0.66ad	0.2ad
RD0040	2686	0.09ad	0.75cd	0	0.06ad	0	0	0.11cd	0.57cd	0.58cd	0	0	0
RD0050	379	0.09ad	2.99cd	0		0	0	0.46cd	0.57cd	0	0.34ad	0	0.2ad
RF0010	726	0	0	0	0.06ad	0	0	0	0	0	0	0	0
RF0020	480	0.09ad	0.37cd	0.43ad	0.69ad	0.25ad	1.06ad			0.58ad	0.17ad	0	0
RF0030	506	0	0	0.43ad	0.12ad	0	0		0	0	0.17cd	0	0.2ad
RF0040	778	0.45ad		0.43ad	0.4ad	0.5ad	0			0	0.34ad		0
RF0050	101	0	0	0	0.06ad	0	0	0	0	0	0	0	0
RF0060	98261	0.09ad	0.37cd	0.43ad	0.17ad	0	0			0	0.34cd	0.26cd	0.2ad
RF0070	36899	0	0	0	0	0	0	0	0	0	0	0	0
RF0080	399	1.52ad		1.08ad		1.01ad	1.06ad	10.74cd	10.47cd				0.6ad
RF0090				1.51ad	4.79ad	2.01ad	1.06ad				0.34ad	0.66ad	1.21ad
RF0100	803	17.68ad		23.23ad	18.13ad	6.79ad		13.77cd	17.58cd			10.27ad	10.67ad
RF0110	35689	0.54ad	0.75ad	0.22ad	0.69ad	1.01ad	0			0.58ad	0.51ad		0
RF0120		0	1.12ad	0.43ad	0.23ad	0	1.06ad			0	0	0	0.2ad
RF0130	2382		0.75ad		0.06ad		0		0.57cd	0	0.17cd	0.13cd	0.4ad
RF0140	3451	0.27ad	1.49ad	0.65ad	1.5ad	1.26ad	0			0			0.2cd
RF0150	2073		1.87cd			0.25cd	1.06cd	0.4cd	0.98cd	0.58cd	0.17cd		0
RF0160	2483	0.18ad	0	0	0.06ad	0	0	0.06ad	0.08cd	0	0	0	0
RF0170	240071	1.07ad	0	0.65ad	1.1ad	0	0	1.2cd	0.57cd	0	0	0	0
RF0180	2932	1.43ad		4.09ad	3ad	1.01ad	1.06cd	2.69cd	4.5cd	0.58cd	0.51ad	0.4ad	

RF0190	43393	0.36ad	0	0	0.12ad	0.25ad	0	0.06ad	0.08cd	0	0.17ad	0.07ad	0
RF0200	891		1.12cd	0.22ad	0.06cd	0.75ad	0			0.58ad	0.17ad	0.13ad	0.2ad
RF0210	40923	0	0	0.22ad	0	0	0	0	0.16cd	0	0	0	0.2ad
RF0230	263479	0.09ad	0.37ad	0	1.27ad	0.25ad	0			0		0.07cd	0
RF0240	98981	0	0.37cd	0	0	0	0	0	0	0	0	0	0
RF0250	215	0	0	0	0	0	0	0	0.08cd	0	0	0	0
RF0270	1467	0.36ad	0.37cd	0	0.06ad	0	0	0.11cd	0	0.58cd	0	0.07cd	0
RF0280	156071		142.28cd	16.13ad	21.65ad				62.46cd				9.06ad
RF0290	97231	0	0	0	0	0	0	0	0	1.16ad	0	0	0
RF0300	104			0.43ad			1.06cd	0.69cd		0.58cd			0
RG0010		0.09ad	4.11cd	1.51ad	3.41ad		2.12ad	3.6cd		0	0	0.2ad	0
RG0020	727	0.98ad	3.36cd	0		1.26ad		0.11cd	1.23cd				0
RG0030	767		0.37cd	0.43ad	0.17ad	0.25ad	0	0.23cd	0.08cd	0.58ad		0.2ad	0.2cd
RG0040	2331	0.36ad	10.08cd	2.15ad	4.21ad	2.51ad	0		2.37cd	0.58ad	1.69ad	1.12ad	0
RG0050	183	3.48ad		1.94ad	1.67ad	1.01ad	1.06ad					0.79ad	0.2ad
RG0060	375	0.18ad	0.75cd	0	0	0	0	0	0.08cd	0	0	0	0
RG0070	900	2.05ad	2.61cd	0.86ad	1.04ad	2.26ad	0		1.96cd	1.16ad	0.17cd		0.2ad
RG0080	397	5.54ad		2.37ad	2.54ad					0.58ad	0.68ad		0.4cd
RG0090	3287	0.89ad	0.75cd		0.12ad		0		0.98cd	0	0.17ad	0.13cd	
RG0100	774					3.27ad	10.62cd	2.63cd	7.6cd				
RG0110	131	0		0	0	0.25ad	0	0.06cd	0.41cd	0	0.17ad	0.07ad	0.2ad
RI0010	930				6.58ad								6.04ad
RI0030	2070		0	0.65ad	0	0.25ad	0	0	0.82cd	0	0.17cd	0	0.2cd
RI0040		0.09cd	0	0.22ad	0	0	0	0.06cd	0.33cd	0	0.34cd		0
RI0050	171	0.71ad	2.99cd			0.75ad	0		2.37cd	0.58cd	0.17cd	0.07ad	0.2ad
RI0070	2290	0	0	0.22ad	0	0	0	0	0	0	0	0	0
RI0080	36204	0.09ad	0		0	0	0	0.06cd	0.08cd	0	0	0	0
RJ0010	223	0.09ad	0	0	0	0	0	0	0.25cd	0	0.17ad		0
RJ0020	49041	0.98ad	1.12cd		0.23cd		0				0.51ad	0.2cd	0.2ad
RJ0030	37202	3.3ad		1.08ad	7.79ad	2.01ad	3.19ad			1.16ad		1.25ad	3.02ad
RL0020	1656	1.43ad	0.37cd	0.43ad	0.35ad	0.25ad	0	0	0.41cd	0	0.17ad	0.07ad	0
RL0030		4.29ad	6.35cd	2.58ad	2.77ad	1.26ad	5.31cd			1.73ad	2.54ad	4.15ad	1.41ad
RL0040		7.77ad			5.43ad		3.19ad		2.7cd	1.16ad	1.35ad	2.44ad	1.41ad
RL0050	46486	1.61ad	0.37ad			0	0	0.11cd	0.16cd	0	0.17ad	0	0.2ad
RL0060			6.72cd		9.87ad		1.06cd			0.58cd			
RM0010	221	1.7ad			1.96ad		2.12cd		2.37cd		0.17ad	0.79ad	
RM0020	732	3.48ad		2.15ad	2.48ad	0.25ad	0			0			0.2ad

RM0030	809	1.16ad	3.36cd	0.86ad	0.87ad	1.51ad	1.06ad		5.56cd	0.58ad			0.6ad
RM0040	3165	0.36ad	0.75ad	0	0.35ad	0	0	0	0.08ad	0	0.34ad		0
RM0050		0	0	0	0	0	0	0.17cd	0	0.58cd	0	0	0
RM0060	728	0.45ad	0	0	0.12ad	0.25ad	0	0.11cd	0.16cd	0	0	0.2ad	0
RN0010		1.43ad		3.66ad	1.04ad	4.27ad	5.31ad			5.2ad			
RN0020	199642	0		0.86ad	0.29ad	0	0		1.31cd	0	0.51ad	1.05ad	0
RN0030		0	0	0	0	0	0	0.11cd	0.08cd	0	0	0	0
RN0040	475	0.18ad	0.37ad	0	0	0	0	0.06cd	0.57cd	0	0	0.13ad	0
RN0050		0	0	0.22ad	0.12ad	0.25ad	0		0.57cd	1.73ad	0.17ad	0.07ad	0
RN0060	2162	0.09ad	0.37ad	0	0.12ad	0	0	0.06cd		0	0.17ad	0.07cd	0.2ad
RN0080	1764	0	0	0.22ad	0.06ad	0	0	0.11cd	0	0	0	0	0
RN0090		0	0	0	0.06ad	0	0	0	0	0	0	0	0
RN0100	708	0.09ad	0	0	0	0	0	0	0	0	0	0	0
RN0110	77		0	0	0.17ad	0	0	0.23cd	0.08cd	0	0	0.07ad	0
RN0120		0.18ad	0.37ad	0.43ad		0	0	0.17cd	0.33cd	0	0.17ad	0.13ad	0
RN0130	35737	0	0	0	0	0	0	0.11cd	0	0	0	0.07ad	0
RN0140		0	0	0	0	0	0	0	0	0	0	0	0
RN0150	1059	0	0	0	0	0	0	0.06cd	0	0	0	0.07ad	0
RN0160			0.75cd	0.43ad	0.69ad	1.51ad	1.06ad	0.34cd	0.33cd	0	0	0.13cd	0
RN0170	1201	0	0	0.43ad	0.12ad	0	0	0.06cd	0.33cd	0	0	0.07ad	0
RN0180	1203	0	0.37cd	0	0.06ad	0	0	0	0.08cd	0	0	0	0.2ad
RN0190	96346		1.12cd	0.43ad	0.23ad		0	0.11cd	0.9cd	0.58cd	0.34cd	0.66cd	0.2ad
RN0200	388		1.49cd			0.5cd	0		1.31cd	0	0.17cd	0.66cd	0
RN0210		0.45ad	0	0.43ad	0.17ad	0	0		0.9cd		0.17cd	0.66ad	0
RN0220	53035	0.18ad	0	0	0.06ad	0.25ad	0	0	0.33cd	0	0.17ad		0
RN0230	2924	0.18ad	0	0.22ad	0.17ad	0	0	0.06cd	0.25cd	0	0	0.07ad	0.2ad
RN0240	2138	0	0	0	0	0	0	0	0	0	0	0	0
RN0250	1309	0.18ad	0	0.65ad	0.06ad	0	0	0.06ad	0.16cd	0	0	0	0.2ad
RN0260	294975	0	0	0	0.06cd	0	0	0.06cd	0.33cd	0	0	0.07cd	0
RN0270	3181	0	0.37cd	0	0	0	0	0	0.08cd	0	0	0	0
RN0280	950	0	0	0	0.06ad	0	0	0	0	0	0	0	0
RN0290		0.09cd	0	0	0	0.25ad	0	0	0	0	0	0	0
RN0300	3027	0	0	0	0	0	0	0.06cd	0.08cd	0	0		0.2cd
RN0310	2345	0.09ad	0		0.06ad	0.25ad	0	0.11cd	0.25cd	0	0		0
RN0320	2368	0.18ad	0	0.43cd	0.29ad	0	0	0	0	0	0	0	0
RN0330	98249	1.43ad		1.08ad	5.54ad	1.76ad	2.12ad			1.16ad	0.17ad	1.25ad	0.4ad
RN0340	974	0	0	0	0.06ad	0	0	0.11cd	0	0	0	0	0

RN0350	192	0	0	0	0	0	0	0.11cd	0	0	0	0	0
RN0360	1465	0	0	0	0.17ad	0	0			0	0	0	0
RN0370	239	0	0	0	0	0	0	0	0	0.58cd	0	0	0
RN0380	3255	0	0	0	0.06ad	0	0	0	0	0	0	0	0.2ad
RN0390	380	0.09ad	0	0	0	0.25ad	0	0	0	0	0	0.07ad	0
RN0400	1540	0	0.75cd	0	0	0	0	0	0	0	0.17ad	0	0
RN0410	2311	0	0	0	0.12ad	0	0	0	0.16cd	0	0	0	0.2cd
RN0430	2911		0.75cd	0.22ad			0	1.83cd	1.39cd	0.58cd			
RN0470	669	0	0	0	0.06ad	0	0	0.17cd	0	0	0	0	0
RN0480	3377	0	0	0	0	0	0	0.06cd	0	0	0	0	0
RN0500		0	0	0	0.06ad	0	0	0	0	0	0	0	0
RN0510	464	0.09ad	0	0		0	0	0.11cd	0.08cd	0	0	0.07ad	0
RN0520	910	0	0	0	0	0	0		0	0	0	0.07ad	0
RN0530		0.09ad	0	0	0.06ad	0	0	0	0	0	0	0	0
RN0540	1556	0	0	0	0	0	0	0.06cd	0	0	0.17ad	0	0
RN0550	218	1.34ad	0.37cd	0.43ad	0.23ad	0.5ad	0	0.23cd	0.9cd	0	0.34ad	0.4ad	0.4ad
RN0560	1775	0	0	0.22ad	0	0	0	0	0	0	0	0.07ad	0
RN0570		0.27ad		0	0.35ad	0.5ad	1.06ad		2.21cd	1.16cd		0.72ad	0
RN0580	316	0	0	0	0	0	0	0	0	0	0	0	0.2ad
RN0590	317	0.09ad	0	0	0	0	0	0	0	0	0	0	0
RN0600	312	0	0.37cd	0	0.12ad	0	0	0.11cd	0.16cd	0	0	0.07cd	0
RN0610	2092	0	0	0.22ad	0.06ad	0	0	0	0	0	0	0	0
RN0620	2796	0	0	0	0	0	0	0.06cd	0.16cd	0	0	0	0
RN0630	758	0.54ad	0.75cd	0.65ad	0.52ad	0.25ad	1.06ad			0.58ad	0	0.2ad	0.4ad
RN0640	1114	0.09ad	0	0	0.12ad	0	0	0	0	0	0	0	0
RN0650	1214	0.18ad	0	0	0		0	0.17cd	0	0	0.17cd	0.07cd	0
RN0660	870	3.39ad	17.18cd	6.45ad		3.52ad	2.12cd	42.85cd	13.24cd	4.62cd		4.35ad	2.42ad
RN0670	281	0.27ad	0	0	0.12ad	0.25ad	0	0.34cd	0.33cd	0	0	0.07ad	0
RN0680	881		5.98cd		1.39ad	1.01ad	0	8.91cd	5.31cd			0.86ad	
RN0690		3.66ad	8.22cd		2.77ad			5.49cd	4.09cd	1.73ad			
RN0700	280	0.09ad	0	0	0.06ad	0	0	0.51cd	0.33cd	0	0.17ad	0	0
RN0710	550	2.05ad	0.37ad	0.86ad	0.87ad	0.25ad	0			0		0.33ad	0.4ad
RN0720	551	0.09ad	0	0	0.23ad	0	0	0	0.08cd	0	0	0	0.2ad
RN0740		0	0	0	0.12cd	0	0	0	0	0	0	0	0
RN0750	805	1.16ad		2.15ad	0.23ad	1.26ad	2.12ad	2.46cd	1.88cd	0			
RN0760	2869	0.09ad	1.12cd	0	0.4ad	0	0	0.11cd	0.25cd	0	0.17ad	0.07ad	0
RN0770	3205		0	0	0.06ad	0.25ad	0		0.16cd	0	0.17cd		0

RN0780	892		1.12cd	0.22ad	0.23ad	0.25ad	0		0.25cd	0	0	0	0
RN0790	915	0.27ad	0.37cd	0	0	0.25ad	0		0	0	0	0.07ad	0
RN0800	83	0.09ad	0	0	0	0	0	0	0	0	0	0	0
RN0810	1225	0	0	0	0	0	0	0	0.08ad	0	0	0	0
RN0820	116?	0.36ad	1.87cd	0.65ad	0.35ad	0.25ad	1.06ad	3.14cd	0.65cd	0	0	0.26ad	0.2ad
RN0830	125	0	0	0	0	0	0	0.06cd	0	0	0	0	0
RN0850	138		0	0	0.06ad	0.5ad	0	1.37cd	0.25ad	0	0	0.07ad	0
RN0860	3157	0.09ad	0	0	0.12ad	0	0	0	0.41cd	0	0.17cd	0	0
RN0870	235	0	0	0	0	0	0	0.29cd	0	0	0	0	0
RN0880	1896	0.18ad	0	0	0.35ad	0.25cd	0			0.58ad	0.17ad	0.07ad	0.2ad
RN0890	2053	0	0	0	0	0	0	0.06ad	0.08cd	0	0	0	0
RN0900	2059	0	0	0	0	0	0	0	0	0	0	0	0
RN0910	374		0.37cd	0.22cd		0.5ad	0	1.09cd	0.41cd	0			
RN0930	392	0	0	0.22ad	0	0	0	0.23cd	0.08cd	0	0	0.13ad	0
RN0940	2322	0.18ad	0.37cd	0.22ad	0.23ad	0	1.06ad	1.37cd	0.25cd	0.58cd	0.17cd		0
RN0950	98861	0.09ad		0	0.17ad	0.25ad	0		1.06cd	0	0.85ad	0.26ad	
RN0960	163634	0.09ad	0	0.22cd	0	0	0	0.06cd	0	0	0	0	0
RN0970	560	0.09ad	0	0	0	0	0	0	0	0	0	0	0
RN0980	564	0	0	0	0	0	0	0	0.08cd	0	0	0	0
RN0990	570	0	0	0.22cd	0.17ad	0	1.06ad	0.4cd	0.41cd	0	0	0.13ad	0
RN1000	245	0	0	0	0	0	0	0	0	0	0	0	0
RN1010	648	0.36ad	1.12cd		0.98ad	0.75ad	0	3.09cd				0.66ad	0.2ad
RN1020		0	0	0	0.12ad	0.25ad	0		0	0	0	0	0.2ad
RN1040	710	0	0	0	0	0	0	0.17cd	0	0	0	0	0
RN1070	97360	0	0	0	0	0	0	0	0	0	0	0	0
RN1080	813	0.18ad	0.75cd	0		0.25ad	0	1.03cd	0.49cd	0	0.17cd		0
RN1100	808	0	0	0	0	0	0	0.11cd	0	0	0	0	0
RN1120		0	0	0	0	0	0	0.06cd	0.08cd	0	0	0	0
RN1130	1297	0	0	0	0.06ad	0	0	0.06cd	0	0	0.17ad	0	0
RN1140	107	0	0	0.22ad	0.12ad	0	0	0.17cd		0	0	0.07ad	0
RN1150	1340	0.09ad	0	0	0.06ad	0	0	0.46cd		0	0.17ad		0.2ad
RN1170	744	0	0	0	0.12ad	0	0	0.34cd	0.16cd	0	0	0	0
RN1180	324764	0.27cd	0	0.43ad	0.17ad	0	0	0.46cd	0.16cd	0	0	0.07ad	0
RN1190	2614	0.18ad	1.49cd	0	0	0	0	0	0.33cd	0	0		0
RN1200		0	0	0	0	0.25ad	0	0	0.33cd	0	0	0	0
RN1210	819	0	0.75cd	0.22ad	0.17ad	0	0		0.08cd	0	0.17ad	0.13ad	0.2ad
RN1220	828	0	0.75cd	0.43ad	0.12ad	0.25ad	0	0.11cd	0.33cd	0	0.17ad	0	0

RN1240	857	0	0	0	0.06ad	0	0	0	0	0	0	0	0
RN1250	887	0.09cd	0	0		0	0	0.34cd	0.41cd	0	0.17ad	0.13ad	0
RN1270	904	0.63ad	0	0.22ad	0.23ad	0.25ad	0	3.94cd	1.31cd	0.58ad		0.59ad	0
RN1280	3460	0	0	0	0	0	0	0	0	0	0	0	0
RN1290	3463	0.09ad	0	0	0.06cd	0	0	0.23cd	0.08cd	0	0	0	0.2cd
RN1300	72	0.54ad	0	0.22ad	0.23ad	0	1.06ad	0.69cd	0.9cd	0.58cd	0		0.2ad
RN1310	739	0.18ad		0	0.35ad		0		1.64cd	0.58cd			0.81ad
RN1320	558	3.48ad	1.87cd	0.86ad	2.94ad		0			1.73ad		1.32ad	0.4ad
RN1330	908	2.14ad	1.12ad		0.46ad	1.26ad	1.06ad		1.31cd	0.58ad	0.51ad	1.05ad	0.81ad
RN1350		0.09ad	0.37ad	0	0	0.25ad	1.06cd	0.29cd	0	0	0	0.2ad	0.2ad
RN1360	63	0.63ad			0.29ad	0.75ad	0		1.06cd	0	0.68ad	0.26ad	0.4ad
RN1370	64	0.09ad	0	0	0	0	0	0.11cd	0	0	0	0	0
RN1380	110	0.18ad	0.37ad	0	0.12ad	0	0	0.17cd		0	0	0.07ad	0
RN1410	199	0.18ad	0	0.22ad	0.12ad	0.75ad	1.06ad	0.57cd	0.16cd	0	0.17ad	0.07ad	0
RN1430	220	0	0	0	0	0	0	0	0.16cd	0	0	0	0
RN1440	2710	0	0	0	0	0	0	0	0.08cd	0	0	0	0
RN1450	94068	0	0	0.22ad	0.12ad	0	0	0.17cd	0.16cd	0.58cd	0.17ad	0	0
RN1460	2052	0	0	0	0	0	0	0	0	0	0	0	0
RN1480	435	0.27ad	0	0	0.06ad	0	0	0.29cd	0.16cd	0	0	0.07ad	0
RN1490		0	0	0	0.06ad	0	1.06ad	0.06ad	0.08ad	0	0	0	0
RN1500	477	0	0	0	0	0.25ad	0	0	0	0	0	0	0
RN1510	90308		0	0.43ad	0.17ad	0.25ad	0	1.66cd	0.41cd	0	0	0.07ad	0.2cd
RN1520	98818	0	0	0	0	0	0	0.06cd	0	0	0	0	0
RN1530	500	0	0	0	0	0	0	0.17cd	0	0	0.17ad	0.2ad	0
RN1570	263440	0	0	0	0.06ad	0	0	0	0	0	0	0	0
RN1580	649	0	0	0	0.06ad	0	0	0	0	0	0	0	0
RN1590	884	0	0	0	0	0	0	0.17cd	0.08cd	0	0	0	0
RN1600	699	0	0	0	0	0	0	0	0	0	0	0	0
RN1610	2905	0	0	0	0	0	0	0	0.08ad	0	0	0	0
RN1620	783	0	0	0.22ad	0.12ad	0	0	0.57cd	0.57cd	0	0	0	0
RN1640	1466	0	0	0	0	0	0	0.06cd	0	0	0	0	0
RN1650	404560	2.86ad	0	0	0.29ad	0.25ad	0	0	0	0	0	0	0
RN1660	35125	0.09ad	0	0	0.06ad	0	0	0	0.08ad	0	0.17ad	0	0
RN1670		0	0.37cd	0	0.06ad	0	0	0	0	0	0	0.07ad	0
RN1680	3352	0	0	0	0	0	0	0.06cd	0	0	0	0	0
RN1690		0	0	0	0	0	0	0	0	0	0.17cd	0	0
RN1700	816	0.09ad	0	0	0	1.01ad	0	0.06cd	1.8cd	0	0.51ad	0.2ad	0

RN1720	3437	0.54ad	0	0.22ad	0.23ad	0.25ad	0	0.06cd	0.08ad	0	0.34ad	0	0.2ad
RN1750	3449	0	0	0	0	0.25ad	0	0.06cd	0.08cd	0	0	0	0
RN1760	912	0	0	0	0.06ad	0	0	0.17cd	0	0	0		0
RP0010	290	0	0	0	0	0	0	1.71cd	0.08cd	0	0	0	0
RP0040	1915	0	1.49ad	0	0.87ad	0.25ad	1.06ad			0	0	0.07ad	0
RP0050	70590	0.09ad	0	0	0.06ad	0	1.06cd	0	10.79cd	1.16cd	0.17cd	0	0
RP0060	415286	0	0	0	0	0	0	0.06cd	0	0	0	0	0
RP0070		0	0	0	0	0	0	0	0	0	0	0.07ad	0
RN0460	1988	0	0	0	0	0	0	0	0.08cd	0	0	0	0
RN0730	3163	0.09ad	0	0	0	0	0	0	0	0	0	0	0
RN1090	798	0	0	0	0	0	0	0.06cd	0	0	0	0	0
RN1110		0	0	0	0	0	0	0	0	0	0	0	0
RN1160	1647	0	0	0	0	0	0	0	0.08cd	0	0	0	0
RN1630	36	0	0	0	0	0	0	0.06cd	0	0	0	0	0
RP0020	1906	0	0	0	0	0	0	0	0.08cd	0	0	0	0
RP0030	1912	0	0	0	0	0	0	0	0.08cd	0	0	0	0
RBG010		5.45ad	11.95cd		4.97ad			30.17cd	15.94cd			5ad	5.44ad
RCG010		2.23ad				0.25ad	2.12cd	0.57cd	3.11cd	1.16ad	0.68ad		0
RCG020			10.46cd		2.19ad		1.06ad	2.51cd	4.82cd				1.01ad
RCG030			15.31cd	0.43ad			1.06cd	1.94cd	6.79cd		1.18cd	0.33ad	
RCG040					3.75ad	5.03ad		16.8cd	29.27cd				0.2cd
RCG050			1.87ad	0	0.4ad	0	0	0.17cd	0.74cd	0.58cd	0.17cd	0.07cd	
RCG060			0.37cd		1.56ad	1.26ad	0		3.19cd	1.16ad		0.99ad	
RCG070		1.07ad			0.81ad		1.06ad				0.34ad	0.66ad	
RCG080		0.98ad		1.94ad	1.62ad	1.76ad	2.12cd	3.43cd			1.69ad		0
RCG090		0.09ad	0	0	0	0	0	0.11cd	0.08cd	0	0	0.13ad	0
RCG100		5.09ad	10.46cd	9.46ad	7.74ad	7.54ad	6.37ad		8.83cd	6.35ad	0		
RCG110			0.37cd		0.98ad	0	0	0.17ad		0	0		0.2ad
RCG120		0.09cd	0	0	0	0	0	0.06cd	0.25cd	0	0	0	0
RCG130		3.66ad		1.94ad	1.56ad		0			0	0.85ad	0.92ad	0.6ad
RCG140		0	0	0		0	0	0.97cd		0		0.13ad	0
RCG150		2.14ad		1.94ad			2.12ad		0.98cd	2.89ad		0.99ad	
RCG160		15.63ad						8.23cd	8.09cd				
RDG010			49.3cd	6.88ad	11.03ad	7.54ad		14.46cd	41.37cd				
RDG020		6.61ad	107.93cd	27.32ad	36.32ad	26.65ad		9.83cd	50.44cd		3.55ad	49.32ad	1.21ad
RDG030		0.09ad	4.48cd	0	0.12ad	0.25ad	0	1.71cd	0.41cd	0.58ad	0.17cd	0.07ad	0
RDG040		0	3.36cd	0.22ad	0	0.25cd	0	0	2.78cd	0.58ad	0.17ad	0	0

RFG010	0.36ad	0.37ad	0.65ad		0	1.06ad	0.63cd		0	0.34ad	0.26ad	
RFG020	0.09ad	0.37ad		0.23ad	0	1.06ad	0	0	0	0		0
RFG030	0	0	0.22ad	0.12ad	0	0	0.17cd	0.41cd	0	0	0.07cd	0
RFG040			5.16ad				24.97cd					
RFG050	1.07ad		1.08ad	2.37ad	1.76ad	1.06ad			0.58ad			
RFG060	7.59ad					5.31cd	13.14cd	8.5cd				
RFG070	2.59ad		1.29ad	1.04ad		1.06ad			0.58cd			2.21ad
RFG080	6.88ad	4.48ad		5.6ad			33.54cd					1.61ad
RFG090	3.84ad		8.6ad	3.75ad	2.51ad	1.06ad	13.71cd	4.91cd	2.31cd		0.66ad	1.81ad
RFG100	0.36ad	0.37ad	0		0.5ad	0	0.17cd	0	0.58cd	0.17ad	0.07ad	0.4ad
RFG110	15.99ad											
RFG120	0.54ad		0	0.29cd		1.06cd	1.37cd	0.25cd		0.34ad		0.2cd
RFG130	0	0.75cd	0.22ad	0.29ad	0	0	0.11ad	0.65cd	0	0	0	0
RFG140	0.98ad		0	1.15ad	0.5ad	0		1.88cd	0	0	0.13ad	0.2ad
RGG010	0.71ad	3.73cd	0.65ad	0.92ad		1.06ad	0	2.86cd	2.31ad	0.34ad	0.4ad	0.6ad
RMG010	13.22ad	30.25cd	7.74ad	16.74ad				22.89cd			12.58ad	1.81cd
RNG010	0.45ad	0.37cd	0.65cd	0.64ad	1.26ad	0			0	0.17cd	0.07ad	0.2ad
RNG020	0.09ad	0.75cd	0.43ad		0.5ad	0	0	0.16cd	0	0	0.07cd	
RNG030	0.09ad	0	0	0.06ad	0	0	0.17cd	0	0	0	0	0
RNG040				2.71ad		3.19ad			0			2.82ad
RNG050		4.85cd				2.12cd	3.89cd	2.37cd	2.31cd			
RNG060			0						1.73ad			0.6cd
RNG070		1.12cd		0.92ad	0.5ad	2.12cd		6.62cd			1.58ad	0.4ad
RNG080	0.98ad	1.12cd	0		0.25ad	1.06ad	6.34cd	1.64cd	0.58cd	0	0.46ad	0.2ad
RNG090	5.89ad		4.52ad	4.1ad	3.52ad		19.48cd	11.45cd		3.72ad	8.96ad	2.42ad
RNG100	6.61ad		1.94ad	5.31ad	3.02ad	3.19ad				1.86ad	4.35ad	
