# Supplementary material for: The Occurrence of 275 Rare Diseases and 47 Rare Disease Groups in Italy. Results from the National Registry of Rare Diseases
Source: Int J Environ Res Public Health. 2018 Jul 12;15(7):1470. doi: 10.3390/ijerph15071470 (PMC6068991; doi:10.3390/ijerph15071470)
Supplement: Supplementary file 1 [file ijerph-15-01470-s001.zip › Table S8.pdf]

Article submitted to International Journal of Environmental Research and Public Health
The Occurrence of 275 Rare Diseases and 47 Rare Disease Groups in Italy. Results from the National Registry of Rare Diseases.
Domenica Taruscio*, Luciano Vittozzi, Adele Rocchetti, Paola Torreri, Luca Ferrari

Table S8. Birth Prevalence of Rare Diseases notified to RNMR (live births 2012-2013)

Record source: Subset 1; Notifying regions: AD and CD regions; Record selection: records with birth date between 01/01/2012 and 31/12/2013 and with date of diagnosis within one year from birth. Total records: 2632.
Note: Records are grouped by exemption code, i.e.: diseases identified with group codes are collectively reported with the group denomination.
ad: value based on AD notifications;
cd: value based on CD notifications;
m: value based on both notification types

		**Live births in the period 2012-2013**	1020027	71605	2166	176423	10695	10265	85794	18763	22133	76247
**RNMR Code**	**ORPHA Code**	**Disease or Disease group** / **Region**	**Italy**	**Piemonte**	**Valle d'Aosta**	**Lombardia**	**PA Bolzano**	**PA Trento**	**Veneto**	**Friuli-Venezia Giulia**	**Liguria**	**Emilia-Romagna**
RB0010	654	Wilms tumor	0.78m	0	0	0	0	0	1.17cd	0	0	0
RB0020	790	Retinoblastoma	3.33m	0	0	3.4ad	0	0		0	4.52ad	2.62ad
RB0050	733	Familial adenomatous polyposis	0.2m	0	0	0	0	0	0	0	0	0
RC0010	199296	Congenital isolated ACTH deficiency	0.2cd	0	0	0	0	0	0	0	0	1.31cd
RC0050	508	Leprechaunism	0.1ad	0	0	0	0	0	0	0	0	0
RC0170		Vitamin D-resistent hypophosphatemic rickets	0.1ad	0	0	0.57ad	0	0	0	0	0	0
RC0180	205	Crigler-Najjar syndrome	0.59ad	0	0	0.57ad	9.35ad	0	0	0	0	2.62ad
RD0010		Hemolytic-uremic syndrome	0.1ad	0	0	0	0	0	0	0	0	0

RD0040	2686	Cyclic neutropenia	0.1ad	0	0	0	0	0	0	0	0	0
RD0050	379	Chronic granulomatous disease	0.29m	0	0	0.57ad	0	0	0	0	0	0
RF0010	726	Alpers syndrome	0.2cd	0	0	0	0	0	0	0	0	2.62cd
RF0030	506	Leigh syndrome	0.2ad	0	0	0.57ad	0	0	0	0	0	0
RF0040	778	Rett syndrome	0.1ad	0	0	0.57ad	0	0	0	0	0	0
RF0060	98261	Progressive myoclonic epilepsy	0.2cd	0	0	0	0	0	0	0	0	0
RF0100	803	Amyotrophic lateral sclerosis	0.2m	0	0	0	0	0	0	0	0	0
RF0130	2382	Lennox-Gastaut syndrome	0.1cd	0	0	0	0	0	0	0	0	1.31cd
RF0140	3451	West syndrome	5.69m	0	0		0	0	4.66cd	21.32cd	9.04cd	6.56cd
RF0200	891	Familial exudative vitreoretinopathy	0.1cd	1.4cd	0	0	0	0	0	0	0	0
RF0280	156071	Keratoconus	0.1cd	0	0	0	0	0	0	0	0	0
RG0040	2331	Kawasaki disease	4.31m	4.19ad	0	3.97ad	0	0	4.66cd	5.33cd	4.52cd	
RG0100	774	Hereditary hemorrhagic telangiectasia	0.1cd	0	0	0	0	0	0	0	0	0
RI0030	2070	Eosinophilic gastroenteritis	0.29cd	0	0	0	0	0	0	5.33cd	0	0
RI0040		Chronic intestinal pseudoobstruction	0.1cd	0	0	0.57cd	0	0	0	0	0	0
RI0070	2290	Microvillus inclusion disease (MVID)	0.1ad	1.4ad	0	0	0	0	0	0	0	0
RI0080	36204	Intestinal lymphangiectasia	0.2cd	0	0	0	9.35cd	0	1.17cd	0	0	0
RL0040		Bullous pemphigoid	0.1ad	0	0	0	0	0	0	0	0	0
RN0010		Arnold-Chiari malformation	1.86m	4.19ad	0	0.57ad	0	0	2.33cd	0	0	1.31cd
RN0020	199642	Microcephaly	0.29m	0	0	0	0	0	0	0	0	1.31cd
RN0040	475	Joubert syndrome	0.2m	0	0	0	0	0	1.17cd	0	0	0
RN0050		Lissencephaly	1.08m	0	0	1.13ad	0	0	1.17cd	0	0	1.31cd
RN0060	2162	Holoprosencephaly	0.59m	1.4ad	0	0	0	0	0	0	0	0
RN0090		Axenfeld-Rieger anomaly	0.1ad	0	0	0	0	0	0	0	0	0
RN0100	708	Peters anomaly	0.1cd	0	0	0	0	0	1.17cd	0	0	0
RN0110	77	Aniridia	0.49m	0	0	0	0	0	0	0	0	2.62cd
RN0120		Congenital colobomatous optic disc	0.69m	0	0	0	9.35cd	9.74cd		0	0	1.31cd
RN0140		Pupillar membrane persistence	0.1cd	0	0	0	0	0	0	0	0	1.31cd
RN0160		Esophageal atresia and/or Isolated tracheo-esophageal fistula	6.67m	20.95ad	46.17ad	3.4ad	0	0	12.82cd	15.99cd	4.52cd	7.87cd
RN0170	1201	Atresia of small intestine	1.67m	8.38ad	0	0.57ad	0	0	1.17cd	5.33cd	13.55cd	0
RN0180	1203	Duodenal atresia	1.86m	12.57ad	0	2.27ad	0	0	2.33cd	0	4.52cd	2.62cd
RN0190	96346	Anorectal malformation	6.37m	15.36ad	46.17ad	1.7ad	9.35cd	9.74cd	18.65cd	10.66cd	27.11cd	13.12cd

RN0200	388	Hirschsprung disease	4.22m	8.38ad	0		0	29.23cd	9.32cd	15.99cd	9.04cd	7.87cd
RN0210		Biliary atresia	3.14m	1.4ad	0	1.7ad	0	0		5.33cd	9.04cd	2.62ad
RN0310	2345	Isolated Klippel-Feil syndrome	0.2cd	0	0	0	0	9.74cd	1.17cd	0	0	0
RN0320	2368	Gastroschisis	1.18m	0	0		0	0	1.17cd	0	9.04cd	2.62cd
RN0330	98249	Ehlers-Danlos syndrome	0.1ad	1.4ad	0	0	0	0	0	0	0	0
RN0340	974	Adams-Oliver syndrome	0.2m	0	0	0	0	0	1.17cd	0	0	0
RN0380	3255	Filippi syndrome	0.1ad	0	0	0	0	0	0	0	0	0
RN0390	380	Greig cephalopolysyndactyly syndrome	0.2ad	0	0	0	0	0	0	0	0	0
RN0400	1540	Jackson-Weiss syndrome	0.1ad	0	0	0	0	0	1.17ad	0	0	0
RN0410	2311	Jarcho-Levin syndrome	0.1cd	1.4cd	0	0	0	0	0	0	0	0
RN0430	2911	Poland syndrome	1.27m		0	1.13cd	0	0	0	5.33cd	0	0
RN0470	669	Otopalatodigital syndrome	0.1ad	0	0	0	0	0	0	0	0	0
RN0510	464	Incontinentia pigmenti	0.78m	2.79ad	0	0	0	0	0	0	0	3.93cd
RN0540	1556	Cutis marmorata telangiectatica congenita	0.1cd	0	0	0	0	0	0	0	0	1.31cd
RN0570		Epidermolysis bullosa	1.37m	0	0	0	0	0	1.17cd	5.33cd	0	1.31cd
RN0640	1114	Aplasia cutis congenita	0.1ad	0	0	0	0	0	0	0	0	0
RN0660	870	Down syndrome	35m	60.05ad	0		168.3cd	19.48cd	60.61cd	10.66cd	40.66cd	17.05cd
RN0670	281	Cri du chat syndrome	0.29m	0	0	0.57ad	0	0	0	0	0	0
RN0680	881	Turner syndrome	1.96m	0	0	0.57ad	0	9.74cd	2.33cd	0	4.52cd	1.31cd
RN0690		Klinefelter syndrome	2.65m	5.59ad	0	0.57ad	9.35cd	0	5.83cd	5.33cd	4.52cd	6.56cd
RN0700	280	Wolf-Hirschhorn syndrome	0.2m	0	0	0	0	0	0	0	0	0
RN0710	550	MELAS	0.59ad	0	0	0	0	0	0	0	0	1.31ad
RN0740		Ivemark syndrome	0.1cd	0	0	0	0	0	1.17cd	0	0	0
RN0750	805	Tuberous sclerosis	2.84m	5.59ad	0	1.7ad	0	9.74cd		0	9.04cd	6.56cd
RN0770	3205	Sturge-Weber syndrome	0.69m	1.4ad	0	0	0	0	2.33cd	0	0	1.31cd
RN0790	915	Aarskog-Scott syndrome	0.1ad	0	0	0	0	0	0	0	0	0
RN0810	1225	Baller-Gerold syndrome	0.1ad	0	0	0	0	0	0	0	0	0
RN0820	116?	Beckwith-Wiedemann syndrome	2.65m	6.98ad	0	0	9.35cd	0	4.66cd	0	0	2.62cd
RN0850	138	CHARGE syndrome	1.18m	1.4ad	0	1.13ad	0	0	1.17cd	5.33ad	9.04cd	1.31cd
RN0860	3157	De Morsier syndrome	0.2ad	1.4ad	0	0	0	0	0	0	0	0
RN0880	1896	EEC syndrome	0.59m	2.79ad	0	0	0	0	0	0	0	0
RN0890	2053	Freeman-Sheldon syndrome	0.1cd	0	0	0	0	0	1.17cd	0	0	0
RN0900	2059	Fryns syndrome	0.1cd	0	0	0	0	0	1.17cd	0	0	0

RN0910	374	Goldenhar syndrome	1.67m	0	0	0	0	9.74cd	1.17cd	0	4.52cd	1.31cd
RN0940	2322	Kabuki make-up syndrome	0.1ad	0	0	0	0	0	0	0	0	0
RN0950	98861	Primary ciliary dyskinesia, Kartagener type	1.08m	1.4ad	0	1.13ad	0	9.74ad	2.33cd	0	4.52cd	
RN0990	570	Moebius syndrome	0.29m	0	0	0	0	0	1.17cd	0	0	0
RN1000	245	Nager syndrome	0.1cd	0	0	0	0	0	1.17cd	0	0	0
RN1010	648	Noonan syndrome	2.45m	1.4ad	0	0	0	0	2.33cd	0	0	5.25cd
RN1040	710	Pfeiffer syndrome	0.1ad	1.4ad	0	0	0	0	0	0	0	0
RN1080	813	Silver-Russell syndrome	0.2m	0	0	0	0	0	0	0	0	1.31cd
RN1130	1297	Branchio-oculo-facial syndrome	0.1cd	0	0	0	0	0	0	0	0	1.31cd
RN1140	107	Branchio-oto-renal syndrome	0.1cd	0	0	0	0	0	0	0	0	1.31cd
RN1170	744	Proteus syndrome	0.1ad	0	0	0	0	0	0	0	0	0
RN1180	324764	Trichorhinophalangeal syndrome	0.1cd	0	0	0	0	0	0	0	0	0
RN1210	819	Smith-Magenis syndrome	0.1ad	0	0	0	0	0	0	0	0	0
RN1220	828	Stickler syndrome	0.1ad	0	0	0	0	0	0	0	0	0
RN1240	857	Townes-Brocks syndrome	0.1ad	0	0	0	0	0	0	0	0	0
RN1250	887	VACTERL/VATER association	0.78m	1.4ad	0	1.13ad	0	0	0	0	0	0
RN1270	904	Williams syndrome	1.47m	0	0	0	0	0	0	15.99cd	4.52ad	3.93cd
RN1300	72	Angelman syndrome	0.2ad	1.4ad	0	0	0	0	0	0	0	0
RN1310	739	Prader-Willi syndrome	3.63m	4.19ad	46.17ad	1.7ad	0	0	8.16cd	5.33ad	0	2.62cd
RN1320	558	Marfan syndrome	0.29ad	2.79ad	0	0	0	0	1.17ad	0	0	0
RN1330	908	Fragile X syndrome	0.39m	1.4ad	0	0	0	0	0	5.33cd	0	0
RN1350		Alagille syndrome	0.69m	0	0	0.57ad	9.35cd	0	0	0	0	1.31cd
RN1410	199	Cornelia de Lange syndrome	0.39m	0	0	1.13ad	0	0	1.17cd	0	0	0
RN1460	2052	Fraser syndrome	0.1ad	0	0	0.57ad	0	0	0	0	0	0
RN1480	435	Ito hypomelanosis	0.2ad	0	0	1.13ad	0	0	0	0	0	0
RN1510	90308	Klippel-Trénaunay syndrome	0.39m	0	0	0.57ad	0	0	0	0	0	3.93cd
RN1530	500	LEOPARD syndrome	0.1ad	0	0	0	0	0	0	0	0	0
RN1590	884	Pallister-Killian syndrome	0.2m	0	0	0	0	0	0	0	0	
RN1620	783	Rubinstein-Taybi syndrome	0.29cd	0	0	0	0	0	0	0	0	2.62cd
RN1760	912	Zellweger syndrome	0.29m	0	0	0	0	0	0	0	0	0
RP0010	290	Congenital rubella syndrome	1.47cd	0	0	0	0	0	0	0	0	0
RP0040	1915	Fetal alcohol syndrome	0.39m	0	0	0	0	0	0	0	0	1.31cd
RP0050	70590	Infantile apnea	0.49m	0	0	0	0	0	0	0	4.52cd	0

RBG010	Neurofibromatoses	3.92m	8.38ad	0	3.4ad	0	0	3.5cd	10.66cd	4.52cd	7.87cd
RCG010	Primary hyperaldosteronisms	0.2m	0	0	0	0	0	0	0	4.52cd	0
RCG020	Congenital adrenogenital syndromes	3.14m		0	0.57ad	0	0	4.66cd	5.33cd	0	2.62cd
RCG040	Disturbances of aminoacid transport and metabolism	30.1m	27.93ad	0	31.74ad	18.7cd	0	13.99cd	58.63cd		20.98cd
RCG050	Urea cycle disturbances	1.18m	0	0	0	0	0	3.5cd	0	0	1.31ad
RCG060	Disturbances of carbohydrate transport and metabolism, excluded diabetes mellitus	5.39m	6.98ad	0	4.53ad	0	0		0	0	6.56cd
RCG070	Congenital alterations of lipoprotein metabolism, excluded: heterozygous familial hypercholesterolhaemia type IIa and IIb; polygenic primary hypercholesterolhaemia; combined familial hypercholesterolhaemia; Hyperlipoproteinhaemia type III	3.82m	2.79ad	0	2.27ad	9.35cd	0	0	5.33cd	0	15.74cd
RCG080	Lipid storage disturbances	0.98m	1.4ad	0	1.13ad	0	0	0	5.33cd	0	0
RCG090	Mucolipidoses	0.1ad	0	0	0	0	0	0	0	0	0
RCG100	Congenital alterations of iron metabolism	0.1cd	0	0	0	0	0	0	0	0	0
RCG140	Mucopolysaccharidoses	0.2m	0	0	0	0	0	1.17cd	0	0	0
RCG150	Chronic histiocytoses	0.59m	2.79ad	0	0	0	0	0	0	0	0
RCG160	Primary immunodeficiencies	4.9m	4.19ad	0	3.97ad	9.35cd	0		5.33cd	0	2.62cd
RDG010	Hereditary anemias	20.69m	30.72ad	0	2.27ad	28.05cd	19.48cd	103.74cc	5.33cd	27.11cd	38.03cd
RDG020	Hereditary coagulation defects	5.49m	11.17ad	0	3.97ad	9.35cd	0	5.83cd	0	27.11cd	6.56cd
RDG030	Hereditary thrombocytopathies	0.1ad	1.4ad	0	0	0	0	0	0	0	0
RDG040	Primary hereditary thrombocytopenias	0.2m	1.4ad	0	0.57cd	0	0	0	0	0	0
RFG010	Leucodystrophies	0.39m	0	0	0	0	0	1.17cd	0	0	0
RFG030	Gangliosidoses	0.78m	0	0	0.57ad	0	0	1.17cd	0	0	1.31cd
RFG040	Spinocerebellar diseases	0.29ad	0	0	0	0	0	0	0	0	0
RFG050	Spinal muscular atrophies	3.24m	1.4ad	0	1.13ad	0	0	5.83cd	0	4.52cd	2.62cd
RFG060	Hereditary neuropathies	0.59m	1.4ad	0	0	0	0	0	0	0	1.31cd
RFG070	Hereditary congenital miopathies	0.49m	0	0	0	0	0	1.17cd	0	0	0
RFG080	Muscular dystrophies	1.57m	0	0	0.57ad	0	0	0	5.33cd	4.52cd	1.31ad

RFG090	Miotonic dystrophies	0.69m	0	0	0.57ad	0	0	1.17cd	0	4.52cd	1.31cd
RFG110	Hereditary retinic dystrophies	1.08m	0	0	0	0	0	2.33cd	0	0	
RMG010	Undifferentiated connective tissue syndromes	0.1ad	0	0	0	0	0	0	0	0	0
RNG010	Pseudohermaphroditisms	1.08m	4.19ad	0	0.57ad	0	0	1.17cd	0	0	1.31cd
RNG020	Multiple congenital arthrogryposes	0.69m	0	0	0	0	0	0	0	0	1.31cd
RNG030	Acrocephalosyndactyly	0.1ad	0	0	0	0	0	0	0	0	0
RNG040	Congenital craniofacial anomalies	21.57m	19.55ad	0	13.6ad	74.8cd		72.27cd	10.66cd		41.97cd
RNG050	Congenital chondrodystrophies	3.53m	12.57ad	0	2.27ad	9.35cd	0	2.33cd	0	4.52cd	1.31cd
RNG060	Congenital osteodystrophies	2.84m	5.59ad	0	0	9.35cd	0	0	0	0	3.93cd
RNG070	Congenital ichthyoses	2.94m	11.17ad	0	0	0	9.74ad	3.5cd	0	9.04cd	1.31cd
RNG080	Chromosomal aneuploidy syndromes	2.55m	4.19ad	0	2.27ad	9.35cd	0	1.17cd	0	0	0
RNG090	Chromosomal duplication/deficiency syndromes	8.73m	6.98ad	0	3.4ad	18.7cd	9.74cd	13.99cd	5.33ad	18.07cd	18.36cd
RNG100	Other congenital anomalies with intellectual disability	2.84m	4.19ad	0	1.13ad	0	0	1.17cd	0	0	0

Supplementary File 4 - Table S4.2

RNMR Code	ORPHA Code	Toscana	Umbria	Marche	Lazio	Abruzzo	Molise	Campania	Puglia	Basilicata	Calabria	Sicilia	Sardegna
**Live births in the period 2012-2013**		59832	14947	24934	101456	19550	4187	105169	65128	8389	33514	85376	23454
RB0010	654	3.34ad	0	0	1.97ad	0	0	0.95ad	1.54ad	0	2.98ad	0	0
RB0020	790	0	6.69ad	0	2.96ad	30.69ad	0	2.85ad	6.14ad	0	5.97ad	0	0
RB0050	733	0	0	0	0	0	0	0.95ad	1.54cd	0	0	0	0
RC0010	199296	0	0	0	0	0	0	0	0	0	0	1.17cd	0
RC0050	508	0	0	4.01ad	0	0	0	0	0	0	0	0	0
RC0170		0	0	0	0	0	0	0	0	0	0	0	0
RC0180	205	3.34ad	0	0	0	0	0	0	0	0	0	0	0
RD0010		0	0	0	0	0	0	0	0	11.92ad	0	0	0

RD0040	2686	0	0	0	0.99ad	0	0	0	0	0	0	0	0
RD0050	379	0	0	0	0	0	0	0	1.54cd	0	2.98ad	0	0
RF0010	726	0	0	0	0	0	0	0	0	0	0	0	0
RF0030	506	0	0	0	0.99ad	0	0	0	0	0	0	0	0
RF0040	778	0	0	0	0	0	0	0	0	0	0	0	0
RF0060	98261	0	0	0	0	0	0	0	0	0	0	2.34cd	0
RF0100	803	0	0	0	0.99ad	0	0	0	1.54cd	0	0	0	0
RF0130	2382	0	0	0	0	0	0	0	0	0	0	0	0
RF0140	3451	3.34ad	26.76ad	4.01ad	12.81ad	15.35ad	0	0.95cd	1.54ad	0	14.92ad		4.26cd
RF0200	891	0	0	0	0	0	0	0	0	0	0	0	0
RF0280	156071	0	0	0	0	0	0	0	1.54cd	0	0	0	0
RG0040	2331	3.34ad	6.69cd	8.02ad	13.8ad	5.12ad	0	0	0	0	2.98ad	3.51ad	0
RG0100	774	0	0	0	0	0	0	0	1.54cd	0	0	0	0
RI0030	2070	0	0	0	0	0	0	0	1.54cd	0	2.98cd	0	0
RI0040		0	0	0	0	0	0	0	0	0	0	0	0
RI0070	2290	0	0	0	0	0	0	0	0	0	0	0	0
RI0080	36204	0	0	0	0	0	0	0	0	0	0	0	0
RL0040		0	0	0	0.99ad	0	0	0	0	0	0	0	0
RN0010		0	6.69ad	0	1.97ad	5.12ad	23.88ad	0.95ad	1.54ad	23.84ad	0	1.17cd	8.53ad
RN0020	199642	0	0	0	0.99ad	0	0	0	0	0	2.98ad	0	0
RN0040	475	0	0	0	0	0	0	0	0	0	0	1.17ad	0
RN0050		0	0	0	0.99ad	0	0	0	1.54cd	35.76ad	2.98ad	1.17ad	0
RN0060	2162	1.67ad	0	0	0	0	0	0		0	0	1.17cd	4.26ad
RN0090		0	0	0	0.99ad	0	0	0	0	0	0	0	0
RN0100	708	0	0	0	0	0	0	0	0	0	0	0	0
RN0110	77	0	0	0	0.99ad	0	0	0.95cd	0	0	0	1.17ad	0
RN0120		0	0	4.01ad	0	0	0	0	0	0	0	0	0
RN0140		0	0	0	0	0	0	0	0	0	0	0	0
RN0160		10.03ad	0	4.01ad	8.87ad	25.58ad	23.88ad	0.95cd	1.54cd	0	0	1.17cd	0
RN0170	1201	0	0	8.02ad	0.99ad	0	0	0	3.07cd	0	0	0	0
RN0180	1203	0	0	0	0.99ad	0	0	0	0	0	0	0	0
RN0190	96346	6.69ad	0	8.02ad	0.99ad	10.23ad	0	0	6.14cd	0	2.98cd	0	0

RN0200	388	1.67ad	0	0	4.93ad	0	0	0	4.61cd	0	0	0	0
RN0210		8.36ad	0	4.01ad	0	0	0		1.54ad	11.92ad	0	8.2ad	0
RN0310	2345	0	0	0	0	0	0	0	0	0	0	0	0
RN0320	2368	3.34ad	0	0	0.99ad	0	0	0	0	0	0	0	0
RN0330	98249	0	0	0	0	0	0	0	0	0	0	0	0
RN0340	974	0	0	0	0.99ad	0	0	0	0	0	0	0	0
RN0380	3255	0	0	0	0	0	0	0	0	0	0	0	4.26ad
RN0390	380	1.67ad	0	0	0	5.12ad	0	0	0	0	0	0	0
RN0400	1540	0	0	0	0	0	0	0	0	0	0	0	0
RN0410	2311	0	0	0	0	0	0	0	0	0	0	0	0
RN0430	2911	1.67ad	0	0	0.99ad		0	0.95cd	1.54cd	0	2.98ad	1.17ad	0
RN0470	669	0	0	0	0.99ad	0	0	0	0	0	0	0	0
RN0510	464	1.67ad	0	0	0.99ad	0	0	0	0	0	0	1.17ad	0
RN0540	1556	0	0	0	0	0	0	0	0	0	0	0	0
RN0570		0	0	0	1.97ad	5.12ad	0	1.9ad	1.54cd	0	2.98ad	4.69ad	0
RN0640	1114	0	0	0	0.99ad	0	0	0	0	0	0	0	0
RN0660	870	35.1ad	33.45cd	96.25ad	0.99cd	61.38ad	0	77.97cd	18.43cd	11.92cd		60.91ad	4.26ad
RN0670	281	0	0	0	0.99ad	0	0	0.95cd	0	0	0	0	0
RN0680	881	3.34ad	0	0	3.94ad	5.12ad	0		0	0	0	1.17ad	4.26ad
RN0690		3.34ad	0	0		0	0	1.9cd	0	11.92ad	0	0	0
RN0700	280	0	0	0	0.99ad	0	0	0	1.54cd	0	0	0	0
RN0710	550	1.67ad	0	0	3.94ad	0	0	0	0	0	0	0	0
RN0740		0	0	0	0	0	0	0	0	0	0	0	0
RN0750	805	3.34ad	0	0	0	5.12ad	23.88ad		0	0	0	3.51ad	4.26ad
RN0770	3205	0	0	0	0.99ad	5.12ad	0	0	0	0	0	1.17ad	0
RN0790	915	0	0	0	0	0	0	0	0	0	0	1.17ad	0
RN0810	1225	0	0	0	0	0	0	0	1.54ad	0	0	0	0
RN0820	116?	5.01ad	0	8.02ad	3.94ad	0	0		0	0	0	1.17ad	0
RN0850	138	1.67ad	0	0	0	10.23ad	0	0.95cd	0	0	0	0	0
RN0860	3157	1.67ad	0	0	0	0	0	0	0	0	0	0	0
RN0880	1896	1.67ad	0	0	0.99ad	5.12cd	0	0	0	11.92ad	0	0	0
RN0890	2053	0	0	0	0	0	0	0	0	0	0	0	0
RN0900	2059	0	0	0	0	0	0	0	0	0	0	0	0

RN0910	374	6.69ad	0	0	1.97ad	0	0	0	3.07cd	0	2.98ad	3.51ad	4.26cd
RN0940	2322	1.67ad	0	0	0	0	0	0	0	0	0	0	0
RN0950	98861	0	6.69ad	0	0	0	0	0	0	0	0	1.17ad	0
RN0990	570	0	0	0	0.99ad	0	0	0	1.54cd	0	0	0	0
RN1000	245	0	0	0	0	0	0	0	0	0	0	0	0
RN1010	648	1.67ad	0	8.02ad	7.89ad	0	0	0.95cd	0	23.84ad	2.98ad	3.51ad	0
RN1040	710	0	0	0	0	0	0	0	0	0	0	0	0
RN1080	813	0	0	0	0.99ad	0	0	0	0	0	0	0	0
RN1130	1297	0	0	0	0	0	0	0	0	0	0	0	0
RN1140	107	0	0	0	0	0	0	0	0	0	0	0	0
RN1170	744	0	0	0	0.99ad	0	0	0	0	0	0	0	0
RN1180	324764	0	0	0	0	0	0	0.95cd	0	0	0	0	0
RN1210	819	0	0	0	0	0	0	0	0	0	0	1.17ad	0
RN1220	828	0	0	0	0.99ad	0	0	0	0	0	0	0	0
RN1240	857	0	0	0	0.99ad	0	0	0	0	0	0	0	0
RN1250	887	0	0	0		0	0	0	1.54cd	0	0	0	0
RN1270	904	3.34ad	0	0	1.97ad	5.12ad	0	0.95ad	0	0	2.98ad	1.17ad	0
RN1300	72	0	0	4.01ad	0	0	0	0	0	0	0	0	0
RN1310	739	0	6.69ad	0	1.97ad	5.12ad	0		3.07cd	0	0	3.51ad	12.79ad
RN1320	558	0	0	0	0	0	0	0	0	0	0	0	0
RN1330	908	1.67ad	0	0	0	0	0	0.95cd	0	0	0	0	0
RN1350		0	6.69ad	0	0	0	0	0	0	0	0	3.51ad	0
RN1410	199	0	0	0	0	5.12ad	0	0	0	0	0	0	0
RN1460	2052	0	0	0	0	0	0	0	0	0	0	0	0
RN1480	435	0	0	0	0	0	0	0	0	0	0	0	0
RN1510	90308	0	0	0	0	0	0	0	0	0	0	0	0
RN1530	500	0	0	0	0	0	0	0	0	0	0	1.17ad	0
RN1590	884	0	0	0	0	0	0	0	0	0	0	0	0
RN1620	783	0	0	0	0	0	0	0	1.54cd	0	0	0	0
RN1760	912	0	0	0	0.99ad	0	0	0.95cd	0	0	0	1.17ad	0
RP0010	290	0	0	0	0	0	0	14.26cd	0	0	0	0	0
RP0040	1915	0	0	0	2.96ad	0	0	0	0	0	0	0	0
RP0050	70590	0	0	0	0.99ad	0	0	0		0	0	0	0

RBG010	8.36ad	0	0	2.96ad	10.23ad	0	0.95ad	1.54cd	0		2.34ad	0
RCG010	0	0	4.01ad	0	0	0	0	0	0	0	0	0
RCG020	0	0	0		10.23ad	0	1.9ad	1.54cd	11.92ad	2.98ad	3.51ad	4.26ad
RCG040	31.76ad			26.61ad	25.58ad	0		73.7cd	47.68cd	26.85ad	26.94ad	0
RCG050	1.67ad	6.69ad	0	3.94ad	0	0	0	0	0	0	0	8.53ad
RCG060	3.34ad	0	0	13.8ad	5.12ad	0	1.9cd		0	0	4.69ad	8.53ad
RCG070		20.07ad	0	4.93ad	0	23.88ad	1.9cd		0	2.98ad	1.17ad	0
RCG080	3.34ad	0	0	0	0	0	0.95ad	0	11.92ad	2.98ad	1.17ad	0
RCG090	0	0	0	0	0	0	0	0	0	0	1.17ad	0
RCG100	0	0	0	0	0	0	0	1.54cd	0	0	0	0
RCG140	0	0	0	0	0	0	0	1.54ad	0	0	0	0
RCG150	0	13.38cd	0	1.97ad	0	0	0	0	0	0	0	0
RCG160	10.03ad	6.69ad	4.01ad	2.96ad	15.35ad	0	3.8cd		0		2.34ad	4.26cd
RDG010	20.06ad	26.76cd	4.01ad	20.7ad	5.12ad	0	0	15.35cd	0	2.98ad	5.86ad	0
RDG020	3.34ad	0		5.91ad	0	0	0	10.75cd	11.92ad	0	2.34ad	0
RDG030	0	0	0	0	0	0	0	0	0	0	0	0
RDG040	0	0	0	0	0	0	0	0	0	0	0	0
RFG010	0	0	0	0.99ad	0	0	0	0	0	0	1.17ad	4.26cd
RFG030	0	0	4.01ad	1.97ad	0	0	0.95cd	1.54cd	0	0	0	0
RFG040	1.67ad	0	0	0	0	0	0.95ad	0	0	0	1.17ad	0
RFG050	0		4.01ad	9.86ad	5.12ad	0	1.9cd	3.07cd	0	5.97ad		0
RFG060	3.34ad	0	0	0.99ad	0	0	0	1.54cd	0	0	0	0
RFG070	0	0	4.01ad	0.99ad	0	0	0.95ad	1.54ad	0	0	0	0
RFG080	1.67ad	0	0	2.96ad	10.23ad	0			11.92ad	2.98ad	0	0

RFG090	0	0	0	0	0	0	0.95cd	1.54cd	0	0	0	4.26ad
RFG110	3.34ad	0	0	3.94ad	0	0	0.95ad	0	0	0	0	0
RMG010	0	0	0	0	0	0	0	0	0	2.98ad	0	0
RNG010	0	0	0	4.93ad	0	0	0	0	0	0	0	0
RNG020	0	0	4.01ad	2.96ad	5.12ad	0	0	1.54cd	0	0	0	0
RNG030	0	0	0	0.99ad	0	0	0	0	0	0	0	0
RNG040	6.69ad	6.69ad		24.64ad		0			0	8.95ad	4.69ad	4.26ad
RNG050		0	4.01ad		5.12ad	0	2.85cd	3.07cd	0	0	3.51ad	0
RNG060	3.34ad	0	0	9.86ad	10.23ad	0	0	3.07cd	0	8.95ad	2.34ad	0
RNG070	1.67ad	0	8.02ad	2.96ad	5.12ad	0		1.54cd	11.92ad	0	4.69ad	0
RNG080	1.67ad	0	0		5.12ad	23.88ad	3.8cd	4.61cd	0	0	1.17ad	0
RNG090	8.36ad	13.38ad		8.87ad	5.12ad	0		4.61cd	0	8.95ad	12.88ad	0
RNG100	8.36ad	0	12.03ad	5.91ad	0	0	1.9ad		11.92ad	0	2.34ad	4.26ad
