# Supplementary material for: The Occurrence of 275 Rare Diseases and 47 Rare Disease Groups in Italy. Results from the National Registry of Rare Diseases
Source: Int J Environ Res Public Health. 2018 Jul 12;15(7):1470. doi: 10.3390/ijerph15071470 (PMC6068991; doi:10.3390/ijerph15071470)
Supplement: Supplementary file 1 [file ijerph-15-01470-s001.zip › Table S9.pdf]

Article submitted to International Journal of Environmental Research and Public Health
The Occurrence of 275 Rare Diseases and 47 Rare Disease Groups in Italy. Results from the National Registry of Rare Diseases.
Domenica Taruscio*, Luciano Vittozzi, Adele Rocchetti, Paola Torreri, Luca Ferrari

Table S9. Regional incidence data

Note: This table compares the medians of the distributions of regional incidences obtained by two notification practices active in the RNMR. The number of regions making up the distributions is also indicated.

RNMR Code	ORPHA Code	Disease or Disease group	Number of Regions with incidence based on CD notifications	Median of regional incidences based on CD notifications	Number of Regions with incidence based on AD notifications	Median of regional incidences based on AD notifications
RA0020	3452	Whipple disease	5	0.15	5	0.13
RA0030	91546	Lyme disease	6	0.31	4	0.26
RB0010	654	Wilms tumor	9	1.02	6	0.16
RB0040	79665	Gardner syndrome	2	0.46	1	0.12
RB0050	733	Familial adenomatous polyposis	11	2.95	7	1.49
RB0060	538	Lymphangioleiomyomatosis	5	0.27	4	0.46
RC0010	199296	Congenital isolated ACTH deficiency	7	0.40	2	0.15
RC0020	478	Kallmann syndrome	6	1.90	5	0.51
RC0030		Reifenstein syndrome	2	0.13	2	0.10
RC0040	169615	Idiopathic central precocious puberty	8	11.44	8	4.90
RC0060	902	Werner syndrome	2	0.17	1	0.06
RC0080		Total lipodystrophy	4	0.12	6	0.17
RC0090	36397	Dercum disease	2	0.14	6	0.16
RC0100	333	Farber Disease	1	0.58	1	0.03
RC0110		Mixed cryoglobulinemia	5	1.49	8	0.95
RC0150	905	Wilson disease	7	0.88	4	0.53
RC0160	436	Hypophosphatasia	1	0.08	2	0.08
RC0170		Vitamin D-resistent hypophosphatemic rickets	2	0.60	6	0.40
RC0180	205	Crigler-Najjar syndrome	1	0.21	4	0.21
RC0190	91378	Hereditary angioedema	8	1.59	6	0.68
RC0200	60	Congenital alpha-1-antitrypsin deficiency	4	1.83	6	0.31
RC0210	117	Behçet disease	4	7.83	6	3.28
RD0010		Hemolytic-uremic syndrome	6	0.53	7	0.45
RD0020	447	Paroxysmal nocturnal hemoglobinuria	3	0.41	6	0.31
RD0030	761	Henoch-Schönlein purpura	7	1.06	7	1.01
RD0040	2686	Cyclic neutropenia	9	0.42	2	0.07
RD0050	379	Chronic granulomatous disease	4	0.51	5	0.10
RF0010	726	Alpers syndrome	2	0.25	3	0.06
RF0020	480	Kearns-Sayre syndrome	2	0.53	8	0.34
RF0030	506	Leigh syndrome	4	0.37	5	0.20
RF0040	778	Rett syndrome	2	0.86	7	0.43
RF0060	98261	Progressive myoclonic epilepsy	6	0.30	6	0.19
RF0070	36899	Myoclonus-dystonia syndrome	1	0.21	1	0.23
RF0080	399	Huntington disease	7	4.60	8	1.28
RF0090		Idiopathic torsion dystonia	5	1.05	9	1.06

RF0100	803	Amyotrophic lateral sclerosis	8	25.96	9	15.01
RF0110	35689	Primary lateral sclerosis	3	0.54	10	0.64
RF0120		Adrenoleukodystrophy	3	0.27	8	0.22
RF0130	2382	Lennox-Gastaut syndrome	9	0.77	3	0.40
RF0140	3451	West syndrome	5	0.75	6	0.95
RF0160	2483	Melkersson-Rosenthal syndrome	4	0.25	3	0.06
RF0170	240071	Steele-Richardson-Olszewski disease	6	0.75	5	0.74
RF0180	2932	Chronic inflammatory demyelinating polyneuropathy	11	2.69	8	2.22
RF0190	43393	Lambert-Eaton syndrome	3	0.14	6	0.14
RF0200	891	Familial exudative vitreoretinopathy	4	0.60	6	0.21
RF0210	40923	Eales disease	2	0.12	2	0.21
RF0230	263479	Fuchs heterochromic iridocyclitis	5	0.54	6	0.18
RF0240	98981	Essential iris atrophy	1	0.37	1	0.03
RF0270	1467	Cogan syndrome	11	0.37	3	0.08
RF0280	156071	Keratoconus	8	59.96	5	11.86
RF0300	104	Leber hereditary optic neuropathy	6	0.87	1	0.43
RG0010		Rheumatic endocarditis	7	2.86	7	1.13
RG0020	727	Microscopic polyangiitis	9	1.23	4	1.22
RG0030	767	Polyarteritis nodosa	9	0.54	6	0.34
RG0040	2331	Kawasaki disease	8	2.27	9	1.55
RG0050	183	Churg-Strauss syndrome	5	3.24	9	1.48
RG0060	375	Goodpasture syndrome	2	0.41	3	0.15
RG0070	900	Wegener granulomatosis	8	3.27	7	1.04
RG0080	397	Giant cell arteritis	6	7.69	7	2.54
RG0090	3287	Takayasu arteritis	9	0.77	4	0.53
RG0100	774	Hereditary hemorrhagic telangiectasia	4	5.12	2	2.16
RG0110	131	Budd-Chiari syndrome	4	0.25	6	0.19
RI0010	930	Idiopathic achalasia	4	4.89	3	6.04
RI0030	2070	Eosinophilic gastroenteritis	8	0.73	4	0.68
RI0040		Chronic intestinal pseudoobstruction	7	0.33	2	0.37
RI0050	171	Primary sclerosing cholangitis	9	2.37	6	0.73
RI0070	2290	Microvillus inclusion disease (MVID)	1	0.08	2	0.15
RI0080	36204	Intestinal lymphangiectasia	6	0.12	2	0.12
RJ0010	223	Nephrogenic diabetes insipidus	3	0.25	2	0.13
RJ0020	49041	Retroperitoneal fibrosis	9	1.05	4	0.74
RJ0030	37202	Interstitial cystitis	2	2.46	10	1.73
RL0020	1656	Dermatitis herpetiformis	8	1.33	8	0.37
RL0030		Pemphigus vulgaris	8	5.67	11	2.58
RL0040		Bullous pemphigoid	7	7.46	9	3.19
RL0050	46486	Mucous membrane pemphigoid	6	1.22	4	0.29
RL0060		Lichen sclerosus	10	5.45	2	9.70
RM0010	221	Dermatomyositis	8	3.36	5	1.44
RM0020	732	Polymyositis	6	3.03	6	1.87
RM0030	809	Mixed connective tissue disease	7	3.36	10	1.11
RM0040	3165	Eosinophilic fasciitis	4	0.31	7	0.35
RM0050		Diffuse fasciitis	2	0.37	1	0.03
RM0060	728	Relapsing polychondritis	7	0.41	5	0.20
RN0010		Arnold-Chiari malformation	6	7.13	8	3.97
RN0020	199642	Microcephaly	6	0.57	5	0.51
RN0030		Cerebellar agenesis	5	0.08	1	0.03
RN0040	475	Joubert syndrome	6	0.31	5	0.17
RN0050		Lissencephaly	3	0.57	7	0.17

RN0060	2162	Holoprosencephaly	3	0.07	6	0.16
RN0080	1764	Familial dysautonomia	2	0.38	2	0.14
RN0090		Axenfeld-Rieger anomaly	1	0.20	3	0.07
RN0100	708	Peters anomaly	2	0.11	1	0.09
RN0110	77	Aniridia	5	0.23	4	0.07
RN0120		Congenital colobomatous optic disc	5	0.84	6	0.17
RN0130	35737	Morning glory syndrome	1	0.11	2	0.05
RN0150	1059	Blue rubber bleb nevus	1	0.06	2	0.05
RN0160		Esophageal atresia and/or Isolated tracheo-esophageal fistula	8	0.83	6	1.14
RN0170	1201	Atresia of small intestine	7	0.33	5	0.12
RN0180	1203	Duodenal atresia	5	0.37	4	0.17
RN0190	96346	Anorectal malformation	12	1.24	5	0.43
RN0210		Biliary atresia	5	0.90	6	0.33
RN0220	53035	Caroli disease	4	0.33	6	0.16
RN0230	2924	Polycystic liver disease	6	0.51	7	0.20
RN0250	1309	Medullary sponge kidney	5	0.53	7	0.20
RN0290		Camptodactyly	3	0.08	1	0.25
RN0310	2345	Isolated Klippel-Feil syndrome	7	0.30	4	0.17
RN0320	2368	Gastroschisis	5	0.30	2	0.23
RN0330	98249	Ehlers-Danlos syndrome	2	2.25	12	1.20
RN0340	974	Adams-Oliver syndrome	2	0.09	2	0.05
RN0350	192	Coffin-Lowry syndrome	2	0.38	1	0.03
RN0370	239	Dyggve-Melchior-Clausen disease	1	0.58	1	0.03
RN0390	380	Greig cephalopolysyndactyly syndrome	2	0.17	3	0.09
RN0400	1540	Jackson-Weiss syndrome	1	0.75	2	0.12
RN0410	2311	Jarcho-Levin syndrome	3	0.16	1	0.12
RN0430	2911	Poland syndrome	9	1.39	1	0.22
RN0470	669	Otopalatodigital syndrome	1	0.17	1	0.06
RN0500		Cutis laxa	1	0.07	1	0.06
RN0510	464	Incontinentia pigmenti	4	0.12	3	0.09
RN0520	910	Xeroderma pigmentosum	2	0.07	2	0.05
RN0540	1556	Cutis marmorata telangiectatica congenita	3	0.20	2	0.12
RN0550	218	Darier disease	9	0.90	8	0.42
RN0570		Epidermolysis bullosa	8	0.90	7	0.35
RN0580	316	Progressive symmetric erythrokeratodermia	1	0.27	1	0.20
RN0590	317	Erythrokeratodermia variabilis	1	0.15	2	0.08
RN0600	312	Epidermolytic ichthyosis	6	0.09	1	0.12
RN0610	2092	Focal dermal hypoplasia	2	0.25	2	0.14
RN0630	758	Pseudoxanthoma elasticum	5	0.75	10	0.46
RN0640	1114	Aplasia cutis congenita	1	0.08	2	0.10
RN0650	1214	Parry-Romberg syndrome	6	0.17	2	0.16
RN0660	870	Down syndrome	11	6.58	6	3.88
RN0670	281	Cri du chat syndrome	5	0.33	5	0.12
RN0680	881	Turner syndrome	9	3.07	5	1.39
RN0690		Klinefelter syndrome	9	6.38	4	2.82
RN0700	280	Wolf-Hirschhorn syndrome	5	0.33	3	0.09
RN0710	550	MELAS	2	0.66	9	0.57
RN0720	551	MERRF	1	0.08	5	0.20
RN0750	805	Tuberous sclerosis	8	1.56	7	1.16
RN0760	2869	Peutz-Jeghers syndrome	7	0.61	6	0.08
RN0770	3205	Sturge-Weber syndrome	6	0.25	2	0.15
RN0780	892	Von Hippel-Lindau disease	7	0.42	4	0.22

RN0790	915	Aarskog-Scott syndrome	4	0.17	5	0.08
RN0820	116?	Beckwith-Wiedemann syndrome	9	0.82	8	0.35
RN0850	138	CHARGE syndrome	6	0.63	5	0.15
RN0860	3157	De Morsier syndrome	6	0.29	4	0.10
RN0880	1896	EEC syndrome	5	0.75	8	0.27
RN0890	2053	Freeman-Sheldon syndrome	3	0.08	1	0.06
RN0910	374	Goldenhar syndrome	10	0.76	1	0.50
RN0930	392	Holt-Oram syndrome	5	0.21	2	0.17
RN0940	2322	Kabuki make-up syndrome	8	0.31	7	0.23
RN0950	98861	Primary ciliary dyskinesia, Kartagener type	3	0.84	7	0.26
RN0960	163634	Maffucci syndrome	5	0.20	2	0.06
RN0990	570	Moebius syndrome	5	0.22	3	0.17
RN1010	648	Noonan syndrome	6	2.28	6	0.71
RN1040	710	Pfeiffer syndrome	2	0.12	1	0.15
RN1080	813	Silver-Russell syndrome	6	0.45	3	0.18
RN1100	808	Seckel syndrome	2	0.09	1	0.03
RN1120		Simpson-Golabi-Behmel syndrome	2	0.07	1	0.08
RN1130	1297	Branchio-oculo-facial syndrome	2	0.07	2	0.11
RN1140	107	Branchio-oto-renal syndrome	5	0.30	4	0.09
RN1150	1340	Cardiofaciocutaneous syndrome	3	0.46	5	0.10
RN1170	744	Proteus syndrome	4	0.19	3	0.08
RN1180	324764	Trichorhinophalangeal syndrome	5	0.27	3	0.17
RN1190	2614	Nail-patella syndrome	5	0.27	3	0.10
RN1200		Smith-Lemli-Opitz syndrome type 1	2	0.27	2	0.16
RN1210	819	Smith-Magenis syndrome	5	0.27	5	0.17
RN1220	828	Stickler syndrome	4	0.23	6	0.21
RN1240	857	Townes-Brocks syndrome	1	0.77	1	0.06
RN1250	887	VACTERL/VATER association	8	0.38	4	0.16
RN1270	904	Williams syndrome	7	1.28	7	0.45
RN1290	3463	Wolfram syndrome	6	0.08	1	0.09
RN1300	72	Angelman syndrome	8	0.79	7	0.22
RN1310	739	Prader-Willi syndrome	7	1.27	4	0.58
RN1320	558	Marfan syndrome	5	1.90	9	1.13
RN1330	908	Fragile X syndrome	6	1.42	11	0.91
RN1350		Alagille syndrome	8	0.47	7	0.20
RN1360	63	Alport syndrome	3	1.06	8	0.43
RN1370	64	Alström syndrome	2	0.33	1	0.09
RN1380	110	Bardet-Biedl syndrome	4	0.12	6	0.16
RN1410	199	Cornelia de Lange syndrome	7	0.23	8	0.17
RN1450	94068	Congenital spondyloepiphyseal dysplasia	6	0.15	4	0.14
RN1480	435	Ito hypomelanosis	3	0.16	5	0.08
RN1490		Isaacs syndrome	1	0.65	5	0.08
RN1510	90308	Klippel-Trénaunay syndrome	8	0.66	5	0.25
RN1520	98818	Landau-Kleffner syndrome	2	0.10	1	0.08
RN1530	500	LEOPARD syndrome	4	0.12	4	0.13
RN1570	263440	Neuroacanthocytosis	2	0.11	1	0.06
RN1590	884	Pallister-Killian syndrome	3	0.17	1	0.08
RN1620	783	Rubinstein-Taybi syndrome	7	0.27	4	0.10
RN1650	404560	Familial dysplastic nevus syndrome	5	0.27	4	0.27
RN1660	35125	Epidermal nevus syndrome	3	0.21	4	0.09
RN1670		Multiple pterygium syndrome	1	0.37	2	0.06
RN1700	816	Sjögren-Larsson syndrome	4	0.93	4	0.35
RN1720	3437	Vogt-Koyanagi-Harada disease	6	0.35	8	0.24
RN1750	3449	Weill-Marchesani syndrome	2	0.07	1	0.25

RN1760	912	Zellweger syndrome	2	0.10	1	0.06
RP0040	1915	Fetal alcohol syndrome	4	0.24	6	0.56
RP0050	70590	Infantile apnea	5	1.06	2	0.07
RP0070		Congenital hepatic fibrosis	2	0.37	3	0.08
RBG010		Neurofibromatoses	10	12.49	5	5.44
RCG010		Primary hyperaldosteronisms	8	2.28	6	0.92
RCG020		Congenital adrenogenital syndromes	9	3.84	4	1.03
RCG030		Autoimmune polyendocrinopathies	11	1.94	4	1.46
RCG040		Disturbances of aminoacid transport and metabolism	8	7.18	5	4.08
RCG050		Urea cycle disturbances	8	0.49	5	0.40
RCG060		Disturbances of carbohydrate transport and metabolism, excluded diabetes mellitus	6	1.21	5	1.21
RCG070		Congenital alterations of lipoprotein metabolism, excluded: heterozygous familial hypercholesterolhaemia type IIa and IIb; polygenic primary hypercholesterolhaemia; combined familial hypercholesterolhaemia; Hyperlipoproteinhaemia type III	5	3.16	7	0.91
RCG080		Lipid storage disturbances	7	1.69	6	1.65
RCG090		Mucolipidoses	4	0.07	2	0.11
RCG100		Congenital alterations of iron metabolism	8	15.08	8	6.96
RCG110		Porphyrias	2	0.29	5	0.77
RCG120		Disorders of purine and pyrimidine metabolism	4	0.15	1	0.03
RCG130		Primary and familiary amyloidoses	1	0.65	8	1.52
RCG140		Mucopolysaccharidoses	7	0.63	2	0.25
RCG150		Chronic histiocytoses	6	1.23	7	1.94
RCG160		Primary immunodeficiencies	7	8.23	3	4.16
RDG010		Hereditary anemias	8	25.78	5	7.54
RDG020		Hereditary coagulation defects	9	23.01	9	26.65
RDG030		Hereditary thrombocytopathies	10	0.60	7	0.12
RDG040		Primary hereditary thrombocytopenias	3	2.78	6	0.44
RFG010		Leucodystrophies	7	0.65	7	0.37
RFG020		Ceroid lipofuscinoses	3	0.65	5	0.23
RFG030		Gangliosidoses	6	0.15	4	0.10
RFG040		Spinocerebellar diseases	2	13.78	4	3.72
RFG050		Spinal muscular atrophies	5	2.59	9	1.07
RFG060		Hereditary neuropathies	8	8.70	2	7.27
RFG070		Hereditary congenital miopathies	4	0.73	7	1.06
RFG080		Muscular dystrophies	7	6.90	6	3.68
RFG090		Miotonic dystrophies	7	4.60	9	2.51
RFG100		Normo-, hypo- and hyperkalemic paralyses	4	0.20	8	0.26
RFG120		Hereditary choroid dystrophies	8	0.28	3	0.34
RFG130		Corneal degenerations	8	0.50	4	0.16
RFG140		Hereditary corneal dystrophies	7	1.94	5	0.50
RGG010		Thrombotic microangiopathies	6	2.58	9	0.65
RMG010		Undifferentiated connective tissue syndromes	9	23.07	6	12.90

RNG010	Pseudohermaphroditisms	5	0.37	7	0.23
RNG020	Multiple congenital arthrogryposes	7	0.23	4	0.29
RNG030	Acrocephalosyndactyly	4	0.12	2	0.07
RNG040	Congenital craniofacial anomalies	6	8.19	3	2.82
RNG060	Congenital osteodystrophies	7	3.47	2	3.66
RNG070	Congenital ichthyoses	7	1.58	7	0.92
RNG080	Chromosomal aneuploidy syndromes	6	1.38	6	0.60
RNG090	Chromosomal duplication/deficiency syndromes	7	11.45	9	3.72
	**Mean**		2.05		1.04
	**SD**		5.34		2.56
	**Pearson correlation coefficient**		0.79		
