# Supplementary figures and images for: The Occurrence of 275 Rare Diseases and 47 Rare Disease Groups in Italy. Results from the National Registry of Rare Diseases
Source: Int J Environ Res Public Health. 2018 Jul 12;15(7):1470. doi: 10.3390/ijerph15071470 (PMC6068991; doi:10.3390/ijerph15071470)

# Exemption codes notified (N)

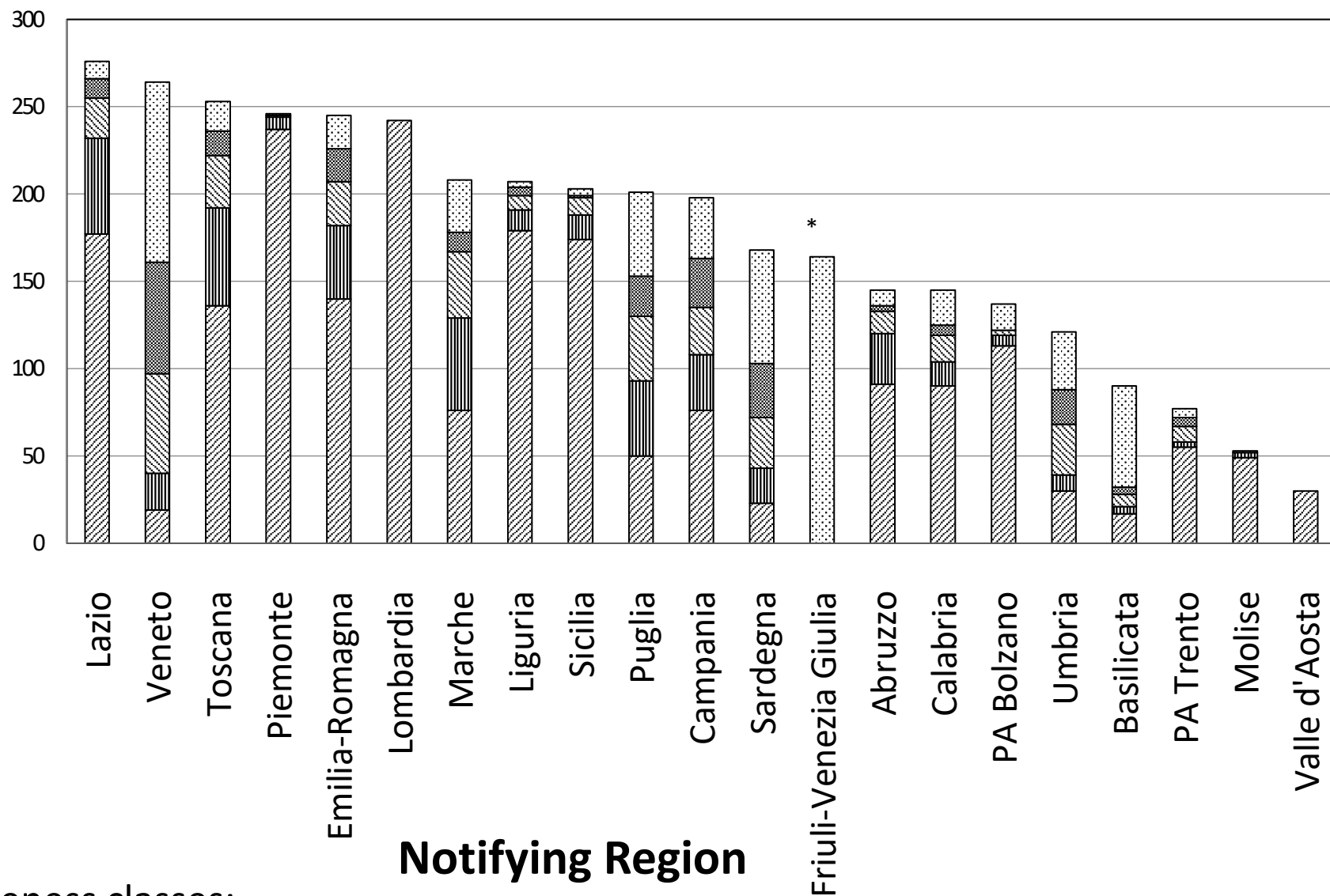

Completeness classes:

0-20%

20-40%

40-60%

60-80%

80-100%

Supplement: Supplementary file 1 [file ijerph-15-01470-s001.zip › Figure S1 of Supplementary file 1.pdf]
